# Ab Initio Thermoelectric Transport Calculations of Alloyed Half‐Heuslers

**DOI:** 10.1002/advs.77630

**Published:** 2026-09-09

**Authors:** Ankit Kumar, Bhawna Sahni, Neophytos Neophytou

**Affiliations:** ^1^ University of Warwick Coventry United Kingdom

**Keywords:** alloy, half‐heuslers, thermoelectrics, transport

## Abstract

We examine the effect of alloying in the electronic transport of half‐Heusler thermoelectric materials from fully ab initio transport considerations. For this, we develop a novel method that computes appropriate supercells of alloys and couples them with Boltzmann transport considering the full energy/momentum dependence of all relevant scattering processes, described using first‐principles extracted parameters. In alloys, such as ${\mathrm{Ti}}_{1-x}{\mathrm{Zr}}_{x}\mathrm{NiSn}$, ${\mathrm{Zr}}_{1-x}{\mathrm{Hf}}_{x}\mathrm{NiSn}$, and ${\mathrm{Hf}}_{1-x}{\mathrm{Ti}}_{x}\mathrm{NiSn}$, only minor electronic structure changes are observed with x. This translates into minor TE power factor changes upon alloying. For TaFeSb‐type materials, isovalent (Nb) and aliovalent (Ti/Zr/Hf) alloying/doping change the band structure significantly, making it more susceptible to band splitting, which leads to significant PF reductions by up to 50%. Thus, although zone‐end high valley degeneracy is favorable for TEs, alloying reduces part of this PF advantage. On the other hand, the additional benefit of thermal conductivity reduction upon alloying still improves the figure of merit zT, showing that in some cases the ultimate performance of these materials can reach a zT = 2 at high temperatures.

## Graphical abstract

The effect of alloying on the electronic transport of half‐Heusler thermoelectric materials is explored using a fully ab initio approach. A novel method is developed that combines calculations of alloyed supercells with Boltzmann transport, considering the full energy and momentum dependent scattering processes from first principles extracted parameters.

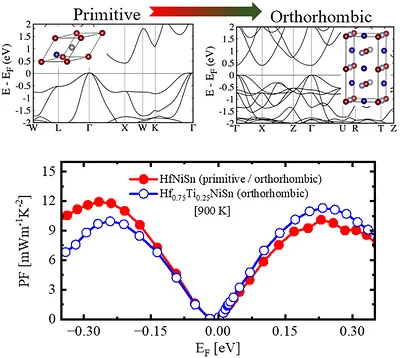

## 1 Introduction

A significant proportion of the available energy from power sources is lost to the environment in the form of waste heat. Solid‐state thermoelectric (TE) materials can convert heat directly into clean electrical energy, offering a path toward sustainability and reduction in the use of fossil fuels [1, 2, 3, 4, 5]. The heat‐to‐electricity conversion efficiency of a TE material is generally quantified by the figure‐of‐merit, zT = $S^{2}\sigma T$/κ, which depends on the Seebeck coefficient (S), electrical conductivity (σ), and thermal conductivity ($\kappa =\kappa_{L}+\kappa_{el}$), where $\kappa_{L}$ and $\kappa_{el}$ are its lattice and electronic components. T is the absolute temperature. These quantities are highly adversely‐interrelated, making it difficult to improve zT. Hence, the key to optimizing TE properties is to identify means of simultaneously enhancing the power factor (PF = $S^{2}\sigma$) and suppressing κ.

Several approaches are considered for enhancing thermoelectric properties. For example, nanostructuring [6, 7], alloying [8], grain boundary scattering and hierarchical phonon scattering [9] are among the widely used techniques to reduce the lattice thermal conductivity. Methods involving band engineering [10], creating resonant states [11], energy filtering [12, 13, 14, 15, 16], and modulation doping [17, 18, 19, 20] improve the PF. Another widely used method to reduce lattice thermal conductivity is isovalent alloying [21, 22, 23], while aliovalent substitution is used to tune carrier concentration without significant thermal conductivity changes [24]. However, any type of substitution of foreign elements in a lattice, will affect the electrical conductivity and PF, and will do so in ways that are currently not understood. Especially when it comes to various substitution types in materials of complex electronic structures, advanced theoretical and computational transport methods could provide insight, but these currently only exist for pristine materials. Within existing computational methods, ab initio calculation of electronic transport for alloyed systems is very challenging, because of two reasons: (1) the computational cost of the required supercell calculation is high, and more importantly 2) supercell calculations result in folded band structures, which introduce additional bands and result in their loss of proper k‐space placement; for the latter, current Boltzmann transport solvers cannot accurately resolve anisotropic scattering processes.

Typical methods to compute the electronic structure of alloys are the KKR‐CPA method which incorporates the disorder in the alloyed system [25, 26, 27, 28], and the virtual crystal approximation (VCA) method [29]. The latter is a much simpler and computationally less expensive approach, which considers a crystal with its the primitive periodicity, but composed of fictitious “virtual” atoms that interpolate between the behavior of the atoms in the parent compounds. This technique has seen wide use in band structure calculations, such as for Si–Ge [30] and AlAs‐GaAS [31] alloyed systems. Since the alloyed Brillouin zone is that of the primitive unit cell, transport calculations are performed as in the pristine system. However, the VCA does not capture correctly the alloying effect in the electronic states in general [32]. Considering a supercell can provide better accuracy for the electronic structure, but a transport simulation method using supercell electronic structures, including all relevant scattering mechanisms with ab initio accuracy, has not yet been developed.

This work presents a unique method to perform electronic transport calculations for alloyed structures with ab initio accuracy. The purpose is to establish a pathway toward electronic transport simulation of alloyed TE materials beyond the constant relaxation time approximation for scattering (CRTA), coupled to electronic structures extracted beyond the VCA. The band structure of alloyed materials needs to be computed on a supercell, which results in folded bands, with subsequent band splittings and broken degeneracies upon alloying, presenting significant numerical challenges when used within transport solvers (BTE). This requires careful methodological handling, which we have developed and present here. For transport we employ the Boltzmann transport code ElecTra [33], which considers the full energy/momentum/band‐dependence of the different states and of the scattering rates. We account for all relevant scattering processes, i.e. acoustic, non‐polar optical, and polar optical phonon (POP), and ionized impurity scattering (IIS).

The paper is organized as follows: In Section 2, we benchmark our new method using the well‐studied mobility trends of Si–Ge alloys, for which sufficient measurements are available. The contribution of alloy scattering and its importance from thermoelectric point of view is also discussed. We then examine the influence of alloying in two sets of half‐Heusler (HH) compound materials. In Section 3, we examine the influence of alloying on HH such as ${\mathrm{Ti}}_{1-x}{\mathrm{Zr}}_{x}\mathrm{NiSn}$, ${\mathrm{Zr}}_{1-x}{\mathrm{Hf}}_{x}\mathrm{NiSn}$, and ${\mathrm{Hf}}_{1-x}{\mathrm{Ti}}_{x}\mathrm{NiSn}$ alloys. In this case, we show that the electronic structure parameters and TE power factor (PF) changes upon alloying follow more or less a linear trend between the end compositions with only small deviations. This is a consequence of the valleys being symmetry‐protected at the Γ‐point. We show that these are cases where alloying reduces the thermal conductivity without significantly affecting the PF from its linear trend with composition. The second category we examine in Section 4, are the TaFeSb‐type HHs, with isovalent (Nb) and aliovalent (Ti/Zr/Hf) alloying/doping. These materials have zone edge valleys and the changes in the band structure upon alloying, especially valley splitting, are significant. We find that alloying in this case reduces the peak PF by up to 50%. Thus, although multi‐valley (X‐ or L‐) materials are in general considered to provide better transport properties, they are more susceptible to band splitting with alloying, which leads to significant reduction in their maximum achievable PF compared to the pristine material. These findings are a step forward in understanding the role of isovalent/aliovalent substitution (alloying) on the PF by examining how it changes the electronic structure, which would be extremely useful in leading experimental efforts toward high PF materials.

## 2 Method for Electronic Transport in Alloys

Here we describe our computational method by considering the well‐studied Si‐Ge alloy material system as an example, for which numerous experimental data exist. Alloy scattering has strong influence on the electron/hole low‐field mobility of Si–Ge at low carrier densities [34, 35, 36]. Here we also examine if this is important in the context of thermoelectric transport, for which carrier density is higher and other scattering mechanisms, such as ionized impurity scattering (IIS) and polar optical phonon (POP) scattering in polar materials (POP), are particularly strong [37, 38].

**FIGURE 1:**
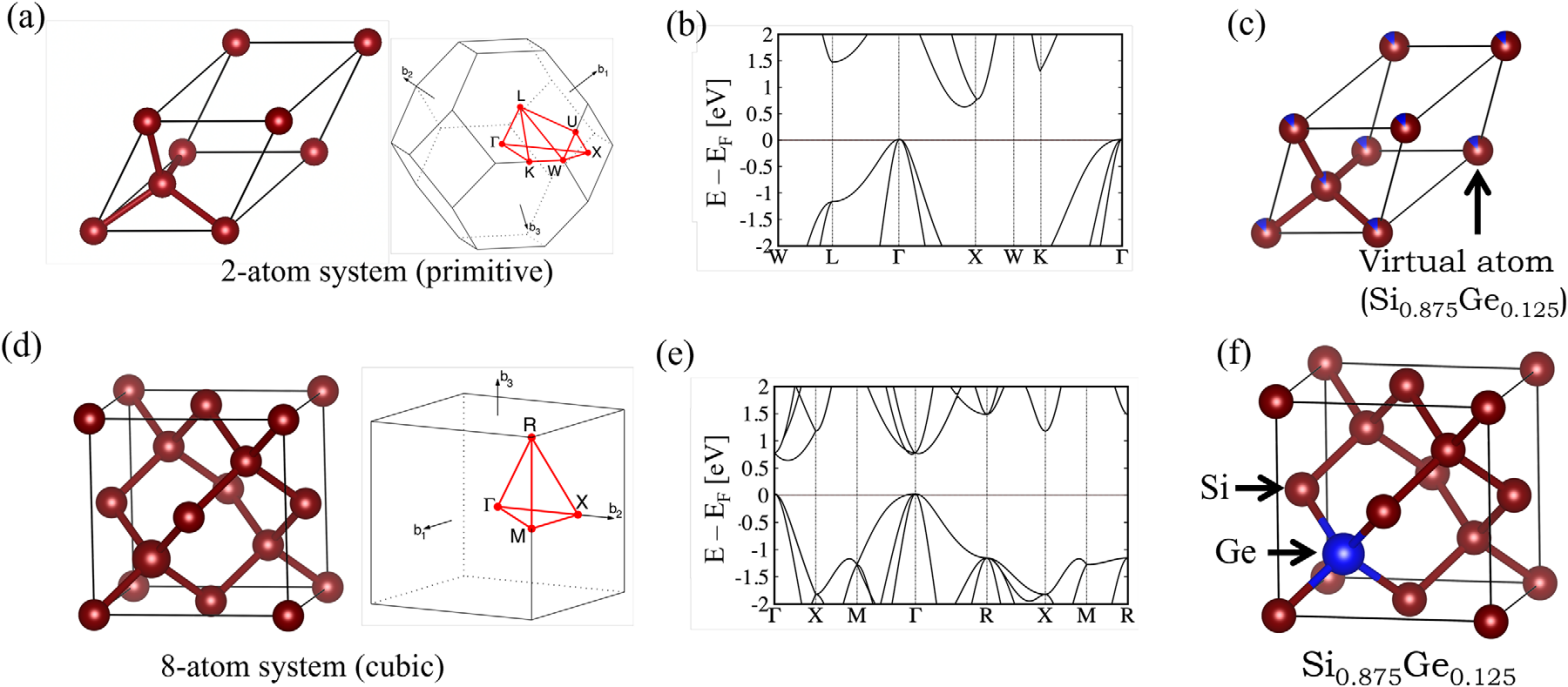
(a) Primitive structure of Si and its Brillouin zone (BZ). (b) Band structure of Si in the primitive BZ. (c) Ge alloyed virtual crystal structure, where each lattice position has both Si and Ge character. (d) Cubic structure (or conventional fcc) and its BZ. (e) Band structure in cubic symmetry. (f) Ge alloyed structure, where one Si is replaced by Ge to create the 12.5% Ge alloyed structure (${\mathrm{Si}}_{0.875}{\mathrm{Ge}}_{0.125}$).

Figure 1a,b shows the primitive crystal structure of Si and its band structure. We first incorporate Ge into the matrix using the VCA (as a reference), where each atom of Si is replaced by a virtual atom according to the desired composition, as shown in Figure 1c. Afterwards we consider the more accurate physical alloying of Ge atoms into the Si matrix using a supercell. We use a conventional fcc structure having eight‐atoms, with reduced symmetry to simple cubic, as shown in Figure 1d. The band structure under this symmetry is shown in Figure 1e. The supercell structure has eight Si atoms, and Ge can be alloyed as shown in Figure 1f. The valence band (VB) maxima at the Γ‐point of the primitive cell is symmetry‐invariant, therefore it remains largely unaffected by the change in structure symmetry. This makes transport straight‐forward for reasons that have to do with retaining the k‐space distance between bands, which we discuss later on, thus in this Section we restrict ourselves to VB calculations. On the other hand, the CB minima at the X‐point of the primitive cell, fold at the Γ‐point of the supercell, which complicates transport treatment; we consider this in a different form at a later Section.

Before considering transport in alloyed systems, we first confirm that the electronic structure and transport properties of the pristine supercell agree with those of the primitive cell. Indeed the density of states and band velocities of the two electronic structures in the VB of pristine Si are the same, as shown in Figure S1a,b. With respect to transport, the transport distribution function (TDF) as expressed in Equation (12), is the basic Kernel of the BTE, which then dictates the electronic transport and the PF (see methods). A comparison of the TDF and PF of the primitive and supercell electronic structures is shown in Figure S1c,d, and again it is almost identical. This validates our transport approach, and we proceed by atomic Ge substitution in the conventional cell (supercell).

**FIGURE 2:**
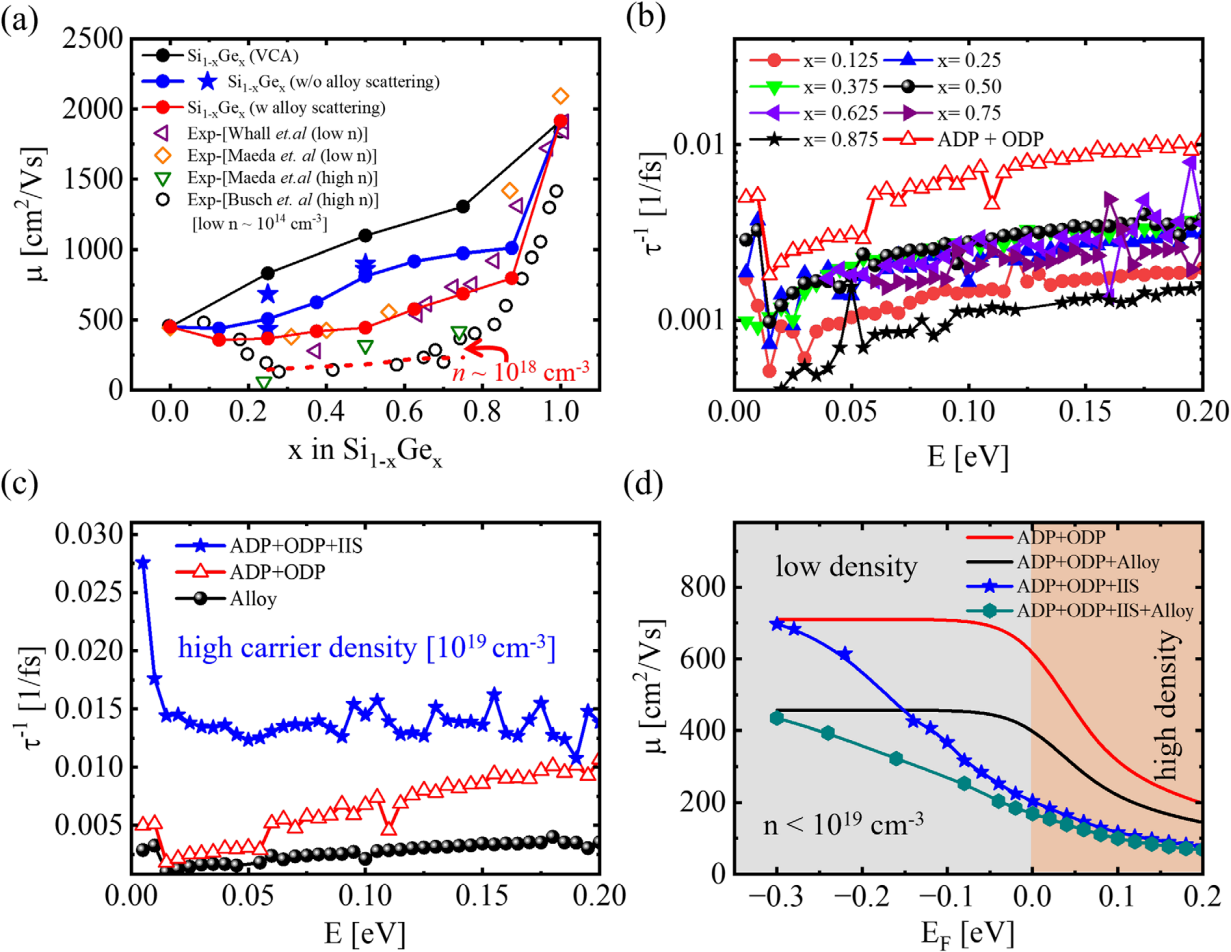
(a) Low density calculated mobility of the virtual crystal (black line‐circle), of the alloyed structure without considering the alloy scattering rate equation (blue line‐circle and scatter star), and of alloyed structure with alloy scattering (red line‐circle). Calculated mobility at high carrier density of 10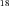 ${\mathrm{cm}}^{-3}$ (red‐dashed line). Reported experimental mobility values for ${\mathrm{Si}}_{1-x}{\mathrm{Ge}}_{x}$ for low and hidh densities as noted [35, 39, 47]. (b) Alloy scattering rates for *x* = 0.125‐0.875 along with the ADP+ODP scattering rate for *x* = 0.5. (c) Scattering rate comparison between ADP+ODP+IIS, ADP+ODP, and alloy‐limited for *x* = 0.5 at high densities. (d) Mobility as a function of Fermi energy for *x* = 0.5 considering ADP+ODP (red), ADP+ODP+IIS (blue) without alloy scattering, and ADP+ODP+Alloy (black) and ADP+ODP+IIS+Alloy (green) scattering.

To better understand the effect of alloying and the different computational methods employed, we first examine the low‐field, low‐density mobility computed using the VCA approach of ${\mathrm{Si}}_{1-x}{\mathrm{Ge}}_{x}$ for *x* = 0.25, 0.5, and 0.75. The parameters used in the transport calculation are listed in Table S1. An acoustic deformation potential (ADP) of 5.0 eV and optical deformation potential (ODP) of 7.63 eV/Å for holes are used to map the experimental mobility of Si (450 ${\mathrm{cm}}^{-2}$ $V^{-1}$ $s^{-1}$ [39]). These deformation potential values are similar to previously reported values [40, 41, 42, 43, 44, 45]. For pristine Ge, we consider deformation potentials of 3.5 eV and 7.45 eV/Å  for ADP and ODP, respectively, parameters that result in a calculated mobility, which matches the experimental mobility (≈ 2000 ${\mathrm{cm}}^{-2}$ $V^{-1}$ $s^{-1}$). In case of alloyed systems ${\mathrm{Si}}_{1-x}{\mathrm{Ge}}_{x}$ we consider a linear variation of the transport parameters (TP) of Si and Ge according to composition, i.e., as (1‐x)${\mathrm{TP}}_{Si}$+${\mathrm{xTP}}_{Ge}$. The VCA calculated mobility shows a linear increase from Si to Ge (Figure 2a, black line), which is expected by VCA construction. This of course deviates significantly from the experimental measurements (stand alone symbols), which follow the typical U‐shape trend.

We next perform the actual supercell alloying calculations, with the intention to examine how much of a closer match to experiment can be achieved by accounting for the actual changes that the band structure undergoes. We considered many compositions of ${\mathrm{Si}}_{1-x}{\mathrm{Ge}}_{x}$ for *x* = 0.125, 0.25, 0.375, 0.5, 0.625, 0.75, and 0.875. The supercell band structures for a few compositions are shown in Figure S2a–c. The change in the hole density of states effective mass (m_dos_) and conductivity effective mass (m_cond_) as a function of Ge alloying for the structures considered, are also shown in Figure S2d, computed using the EFMA code  [46]. Even though the degeneracy remains intact for all Si‐Ge compositions at the Γ‐point, the m_dos_ reduces from 1.5 for Si to 1.0 for Ge. A similar reduction in the m_cond_ is observed, reducing from 0.34 for Si to 0.3 for Ge. This indicates the first order effect of alloying being captured in the electronic states. The mobility in this case starts to form the characteristic U‐shape as a function of Ge fraction (x), but compared to the experimental data, the mobility is still overestimated as shown by the blue‐solid line in Figure 2a. Note that these results are for one random structure for each composition to keep the calculation tangible. In principle many Ge arrangements should be considered with even bigger supercells, especially for *x* ≤ 0.25 and *x* ≥ 0.75, where multiple possible Si–Ge configurations in the 8‐atom structure exist. To obtain a slight indication for this effect on transport, for *x* = 0.25 and 0.5 we considered two more disordered structures, with their mobility, shown in Figure 2a by the blue‐star‐points, being in the proximity of the initial solid‐blue line.

Typically, to capture the effect of alloy scattering, an additional electron‐alloy scattering rate needs to be considered, as given by Equation (7) (see methods). Thus, the change in the band structure seems to capture only partially the alloying effect, while the rest will need to be captured by the consideration of this additional scattering rate. This additional alloy scattering is what accounts empirically for the randomness in the atomic distribution of the different species in the material within a unit cell and from unit cell to unit cell, which cannot be captured by the small unit cells and limited atomic variations that simulations consider. Atomic alloying species change the local potential energy, introducing scattering centers. Typically, the strength of the scattering potential that determines the rate, $\Delta E_{g}$, is not known, and it is used as a fitting parameter to match experimental data. Values between 0.2 and 0.9 eV are commonly used  [36], but this was the case under the VCA, which largely overestimates mobility. In the supercell calculation case, where the actual alloyed structure is considered, we can use a more physical quantity to estimate $\Delta E_{g}$. We use the difference in the workfunction of the alloyed elements, in this case for Si–Ge, $\Delta E_{g}$ = 0.2 eV. Using this, the mobility shows excellent agreement with experimental values, as shown in Figure 2a (red full‐symbol line versus purple open‐triangle and orange open‐diamond symbols). Note that multiple references for Si–Ge mobility data exist, but here we used the ones for which a low carrier density was also reported. We also compare the mobility of the alloyed system for some moderately high carrier densities (here we were able to match partially this trend with densities in the model of 10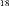 ${\mathrm{cm}}^{-3}$ by the red dotted line in Figure 2a, although experimentally these densities are not reported). Further mobility comparisons at high carrier concentrations with experimental data is shown in Figure S3. In all those cases, the high carrier density mobility is lower than the calculated ones, which is typical. The reasons could be the additional disorder created by ionized impurity doping to achieve such high carrier densities (Boron or Aluminum doping), or the polycrystallinity of the materials, which introduces additional grain boundary scattering, plus additional defects, dislocations, etc., that are often present. In the calculation we are only considering the effect of alloying in pristine Si–Ge alloy.

The alloy scattering rates computed by ElecTra for each composition value, as shown in Figure 2b, reflect the mobility trend. The compositions nearer to the pristine materials (*x* = 0.125, 0.875) have the lowest alloy scattering rates shown by red‐circle and black‐star lines. As, expected, the *x* = 0.5 alloyed system shows the highest scattering rate (black‐circle line). In the latter case the strength of alloy scattering is closer to the phonon (ADP+ODP) limited scattering rate (open red‐triangle line). The phonon‐limited rate is still stronger, but at the middle *x* = 0.5 compositions, alloy scattering is still strong, especially for lower energies and makes the difference in matching the experimental mobility.

The mobility of the alloyed system shown so far was calculated at very low carrier density. At high doping conditions, ionized impurity scattering (IIS) is typically strong, and that regime is the relevant one for TEs  [48, 49]. We next examine the relative effect of alloy scattering at high carrier densities (high doping). We considered the case where alloy scattering is the strongest, i.e., for ${\mathrm{Si}}_{0.5}{\mathrm{Ge}}_{0.5}$. The relevant scattering rates are shown in Figure 2c. Here we show the alloy scattering, phonon, and phonon+IIS rates. We consider a high carrier density of ≈10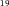 ${\mathrm{cm}}^{-3}$. In this case, phonon+IIS scattering rates overshadow the alloy scattering rates (blue vs. black lines in Figure 2c). The mobility as a function of Fermi energy is shown in Figure 2d. In the low density region (left side), if alloy scattering is ignored, the mobility resides around 700 ${\mathrm{cm}}^{2}$/Vs and drops as the Fermi level increases, dropping faster when IIS is additionally considered (red and blue lines, respectively). When alloy scattering is included in the calculation, the mobility drops to around 420 ${\mathrm{cm}}^{2}$/Vs (black/green lines), and then drops with E_F_ (black and green lines).

In the high carrier concentration region, comparing the blue and green lines, the cases where alloy scattering is neglected versus when it is included, the inclusion of IIS overshadows the alloy scattering effect. The two lines tend to merge after E_F_ = 0, corresponding to the density of p = 10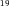 ${\mathrm{cm}}^{-3}$. In the case of polar materials, as the HHs that we examine later, the additional POP scattering that comes into effect is of similar strength to IIS, resulting in alloy scattering having even smaller relative contribution to transport. Therefore, for further calculations of alloyed systems at high densities, the alloy scattering rate formula is not considered. This is also convenient for avoiding any uncertainties in the choice of the different $\Delta E_{g}$. Even with significant uncertainty in $\Delta E_{g}$, the change in mobility or the PF is not significant at high carrier densities, as shown in Figure S4. In the upcoming sections, we discuss the transport properties of XNiSn (X = Ti, Zr, and Hf) and MFeSb (M = Nb and Ta) based alloys, which are known to have excellent thermoelectric properties.

## 3 Thermoelectric Properties of XNiSn (X = Ti/Zr/Hf) Alloys

The (Ti/Zr/Hf)NiSn material system is known for its remarkable *n*‐type thermoelectric properties. To further enhance its zT, an effective way is to alloy Hf at Ti and Zr cites, such as ${\mathrm{Ti}}_{x}{\mathrm{Hf}}_{1-x}{\mathrm{NiSn}}_{1-y}{\mathrm{Sb}}_{y}$  [50], ${\mathrm{Hf}}_{x}{\mathrm{Zr}}_{1-x}{\mathrm{NiSn}}_{0.99}{\mathrm{Sb}}_{0.01}$  [23], ${\mathrm{Ti}}_{0.5}{\mathrm{Hf}}_{0.5}{\mathrm{NiSn}}_{0.98}{\mathrm{Sb}}_{0.02}$  [22]. Using a different synthesis method where ZrNi is used as precursor, ${\mathrm{Zr}}_{0.75}{\mathrm{Hf}}_{0.25}{\mathrm{NiSn}}_{0.99}{\mathrm{Sb}}_{0.01}$ shows the highest reported PF of 5.1 mW ${\mathrm{cm}}^{-1}$ $K^{-2}\mathrm{at}$ 873 K  [51]. However, there are only a few reports on the *p*‐type properties of this class of HHs. To‐date it remains difficult to synthesize *p*‐type XNiSn, because of the presence of excess Ni at the 4d‐site  [52, 53]. For a TE device, both *n*‐ and *p*‐type materials are required, thus it is important to examine the *p*‐type material performance as well. In fact, *p*‐type HfNiSn is only possible by controlling the defects at the 4d‐site  [54], but with continuous improving synthesis techniques, it would be possible to synthesize better quality samples. The following section deals with the alloying transport calculation method and the maximum possible PF that could be achieved in ${\mathrm{Ti}}_{1-x}{\mathrm{Zr}}_{x}\mathrm{NiSn}$, ${\mathrm{Zr}}_{1-x}{\mathrm{Hf}}_{x}\mathrm{NiSn}$, and ${\mathrm{Hf}}_{1-x}{\mathrm{Ti}}_{x}\mathrm{NiSn}$ (*x* = 0.0, 0.25, 0.5, 0.75, and 1.0) in both the *p*‐ and *n*‐type regions.

### 3.1 Electronic Structure Considerations for Alloys

In this section, we use the supercell electronic structures for HH alloys, coupled to the BTE, to calculate the TE transport properties of ${\mathrm{Ti}}_{1-x}{\mathrm{Zr}}_{x}\mathrm{NiSn}$, ${\mathrm{Zr}}_{1-x}{\mathrm{Hf}}_{x}\mathrm{NiSn}$, and ${\mathrm{Hf}}_{1-x}{\mathrm{Ti}}_{x}\mathrm{NiSn}$ (with *x* = 0.0, 0.25, 0.5, 0.75, and 1.0). Our objective is to quantify the effect of alloying on the TE PF at the optimal density, around E_F_ = 0 eV.

**FIGURE 3:**
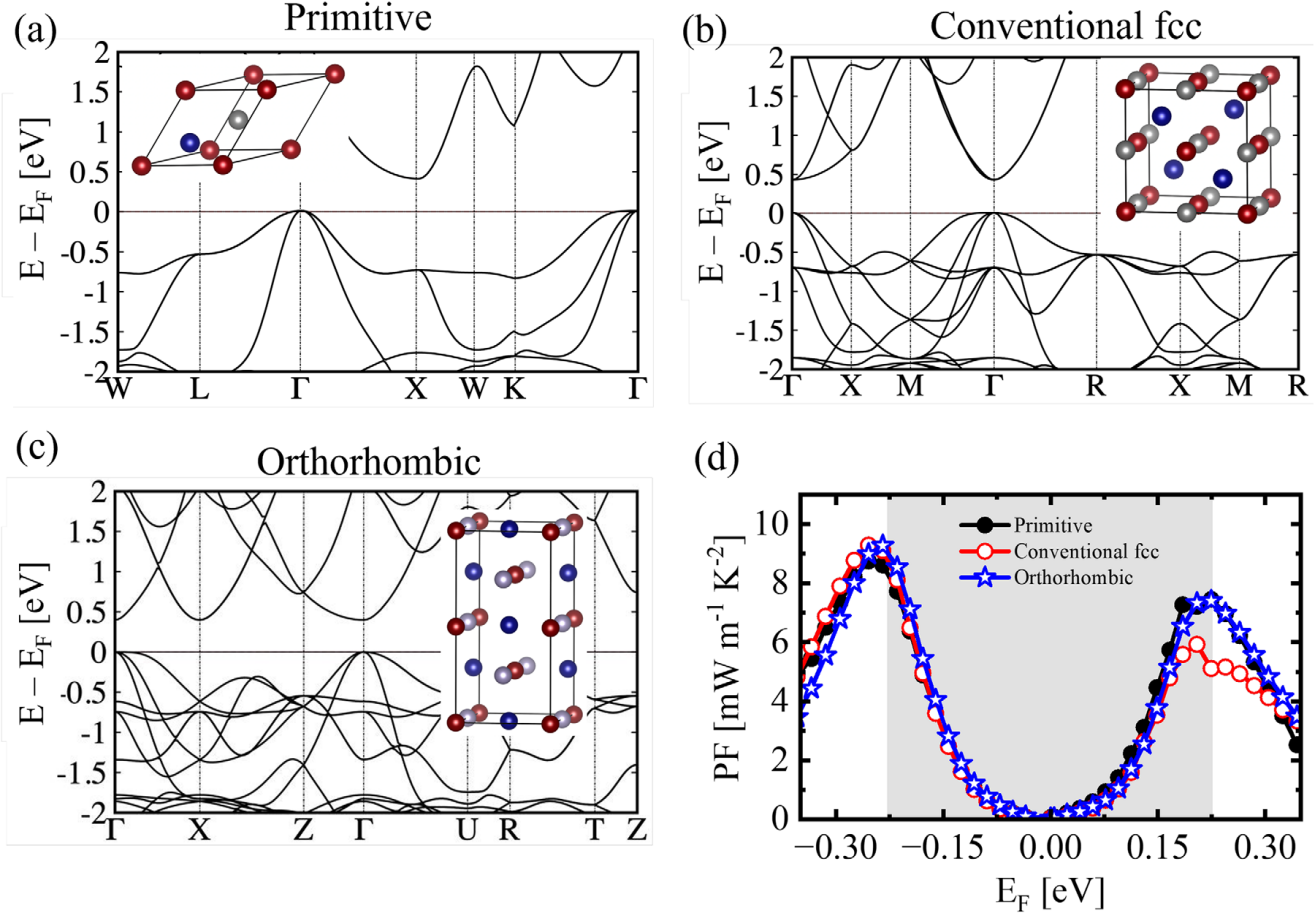
Band structure of HfNiSn for (a) primitive structure, (b) cubic structure (conventional fcc), (c) orthorhombic structure. (d) Power Factor versus Fermi energy calculated for primitive cubic, and orthorhombic structures of HfNiSn at 300 K.

Figure 3a shows the band structure of HfNiSn, calculated using the primitive structure of space group F‐43m. The VB maxima is three‐fold degenerate and Γ‐centered. The CB minima is located at the X‐point, with a three‐fold valley degeneracy. To alloy, we use supercells with a larger number of atoms in order to have flexibility in the alloy compositions. We consider two equivalent structures of reduced symmetry, in order to investigate which one is more suitable to be coupled to the BTE transport calculations. Figure 3b,c shows the band structure of HfNiSn in the conventional fcc (cubic) and orthorhombic unit cells, respectively, both with 12 atoms.

The valence band (VB) maxima at the Γ‐point is symmetry‐invariant, therefore remains unaffected by the change of symmetry from the primitive to cubic to orthorhombic. However, the conduction band (CB) minima at the X‐point, fold differently for the two supercell symmetries. An illustration in Figure S5a shows an example of band folding in the supercell. The high symmetry points of the primitive and supercell BZs are related by the transformation matrix that is used to create the supercell. In the generic illustration of Figure S5a, we show how three different BZ edge valleys (red, blue, and green) fold at one high symmetry point after the formation of a supercell. In the case of the cubic Brillouin zone, they fold at the Γ‐point. However, in the orthorhombic structure, the BZ is folded in a way that two valleys are positioned at the X‐point and one at the Γ‐point, as shown in Figure 3c.

The band folding in supercells complicates the BTE transport calculation. The complication arises from the fact that the supercell loses the correct k‐space placement information of the different band valleys. This is important for the case of the anisotropic scattering mechanisms (POP and IIS), for which the distance of the initial and final scattering states in k‐space determine their scattering strength. In the case of the VB there is no folding involved, so the calculation for *p*‐type transport remains the same as for the primitive BZ. In the case of the CB, however, the three‐fold degenerate valleys of the CB fold on top of each other in the supercell BZ. In this case, we simply consider only intra‐valley/band scattering (our ElecTra code allows for this). This is a valid approximation since inter‐valley scattering is shown to be negligible in valleys that are placed far away in the primitive BZ  [37, 38, 55].

With these considerations, we calculated the PF of all three structures as shown in Figure 3d. We consider both the VB and CB in the same calculation, including bipolar transport as in Ref.  [56]. For both supercells, the *p*‐type PF shows good agreement with the primitive PF calculation (black line). For *n*‐type transport, however, only the orthorhombic supercell transport calculation shows good agreement with the primitive cell (blue line on top of black line). In the conventional fcc BZ, upon closer observation, the folding of the CB on the Γ‐point, mixes the three bands, which eventually leads to mixed Fermi surfaces, and therefore the PF is somewhat different compared to the primitive cell calculation (see Figure 3a,b for the bands and red line in Figure 3d for the PFs). An illustration in Figure S5b shows the mixing of bands, which leads to distinct Fermi surfaces, then to different scattering rates, especially for POP and IIS, which are exchange momentum vector dependent. The advantage of the orthorhombic structure is that such band mixing does not occur. Thus, for this class of materials (XNiSn), we will be using the orthorhombic structure to alloy and compute the PF. A more detailed demonstration of band folding and associated error in the PF because of folding and band mixing is shown in Figure S6. Choosing a conventional fcc or 2×2×2 supercell will result in an estimated error of 20%. It is important to choose the supercell such that band mixing is minimal. It is also important to compare the PF obtained using the primitive structure and supercell to establish the level or error upon alloying. In case of bands with low anisotropy, mixing of bands/Fermi surfaces might not be such an issue. For example in case of the CB of TaFeSb, where the bands are less anisotropic, mixing of the bands will not result in errors and any supercell can be used, as shown in Figure S6d–f

The electronic structures of all compositions in the orthorhombic phase are shown in Figure S7. All the bands in the CB and VB remain degenerate in all alloyed systems with no band splittings. However, there is a significant change in the band curvature, which results in a change in the effective mass and transport properties. Figure S8 shows the density of states effective mass, m_dos_, and conductivity effective mass, m_cond_, for all the alloyed systems, calculated using the EMFA code  [46]. We find that the change in the effective mass is directly correlated with the size difference of the alloying components. Ti‐Zr and Hf‐Ti have large size differences. Ti‐Zr substitution results in 42% and 25% decrease in m_dos_ and m_cond_ when going from TiNiSn to ZrNiSn in the VB. In the CB, the decrease is only 17% and 9%, respectively (Figure S8a,d). Hf‐Ti substitution results in 47% and 33% increase in the m_dos_ and m_cond_ from HfNiSn to TiNiSn in the VB. In the CB the increase is 30% and 31%, respectively (Figure S8c,f). We observe small reductions (5%) for m_dos_ and m_cond_ in both the CB and VB for the Zr–Hf alloy, i.e., going from ZrNiSn to HfNiSn (Figure S8b,e). We have also verified the dynamical stability of these alloyed systems by calculating their phonon dispersions, as shown in Figure S9. The absence of imaginary modes indicates the dynamical stability of the alloyed composition  [57, 58, 59].

### 3.2 Thermoelectronic Transport Properties

**FIGURE 4:**
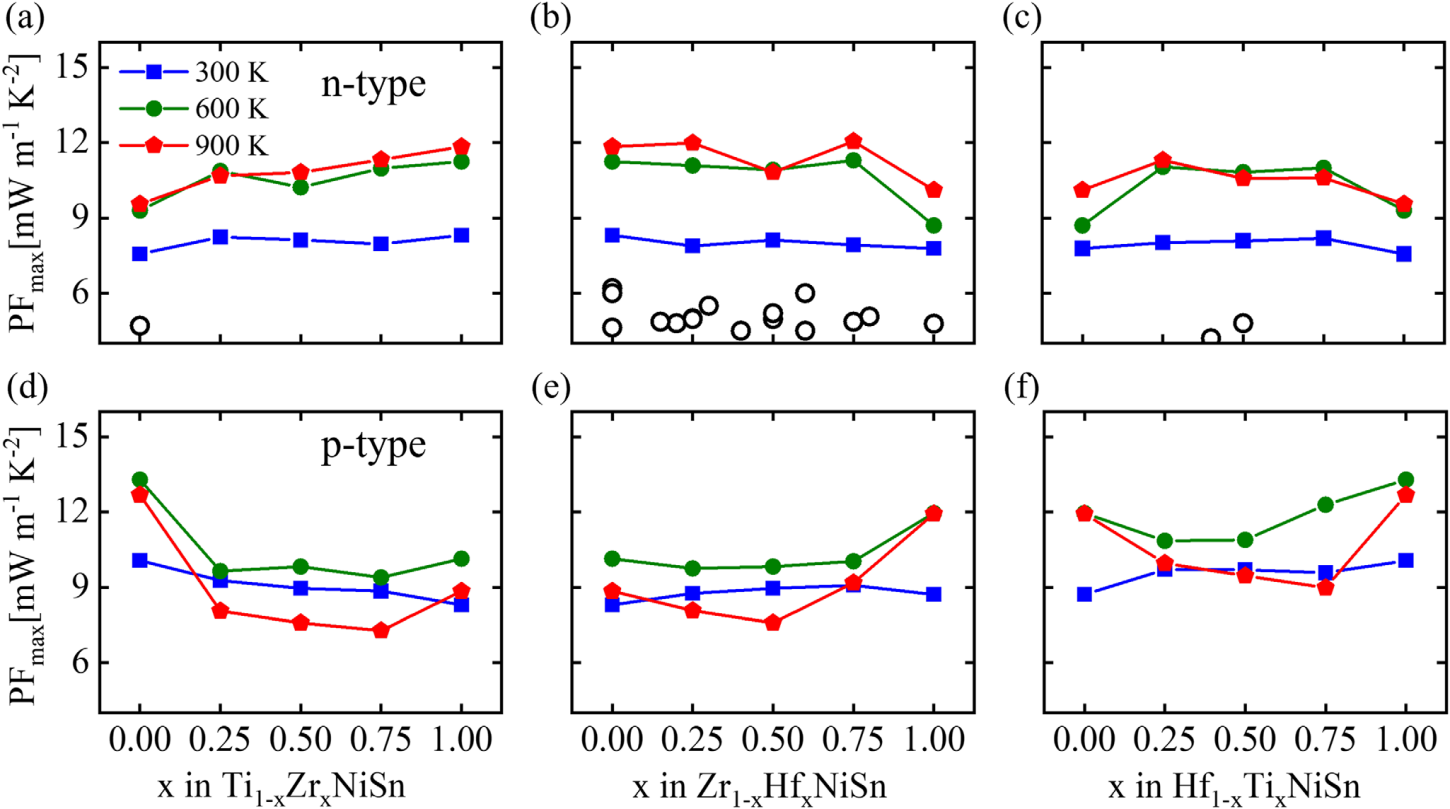
Maximum PF of ${\mathrm{Ti}}_{1-x}{\mathrm{Zr}}_{x}\mathrm{NiSn}$, ${\mathrm{Zr}}_{1-x}{\mathrm{Hf}}_{x}\mathrm{NiSn}$, and ${\mathrm{Hf}}_{1-x}{\mathrm{Ti}}_{x}\mathrm{NiSn}$ for *n*‐type (top row) and *p*‐type (bottom row), for 300 K, 600 K and 900 K. Experimental ${\mathrm{PF}}_{max}$ for a few compositions in the range of 300‐973 K are shown in open circles [22, 23, 50, 51, 60, 61, 62, 63, 64].

We now calculate the PF of these HH alloyed structures using the orthorhombic structure. The input parameters required for the transport calculation in ElecTra are given in Table S2. Ab initio deformation potential calculation is performed using our modified DFT and DFPT+Wannier method  [37, 38]. Other parameters such as sound velocity, dielectric constants, and phonon frequency are also extracted from ab initio DFT calculations. More information on the calculation methods to obtain these parameters are provided in the computational details section. For the alloyed systems, we consider a linear combination of parameters with regards to the alloying composition. We have confirmed the linear trends with calculations. Figure S10a–d shows such linear variation of D_ADP_ (calculated using the band shift method as discussed in Section S2), sound velocity, and $k_{s}$/$k_{\infty }$ for ${\mathrm{Ti}}_{1-x}{\mathrm{Zr}}_{x}\mathrm{NiSn}$ and ${\mathrm{Ta}}_{1-x}{\mathrm{Nb}}_{x}\mathrm{FeSb}$. With regards to the deformation potentials, it is computationally prohibitive to extract them from first principles for alloys, as detailed in the Supporting Information. However, since ADP and ODP are weaker than IIS and POP at high densities relevant for TEs, slight uncertainty in these parameters will not affect our PF results. As a test case we varied the D_ADP_ and D_ODP_ values by 20% from their linear interpolated values for ${\mathrm{Ti}}_{0.5}{\mathrm{Zr}}_{0.5}\mathrm{NiSn}$ and obtained the deviation in PF to be less than 5% (see Figure S11). Similarly, in the context of alloy scattering, as discussed for Si, alloy scattering is also overshadowed by IIS at high carrier concentrations relevant for TEs (10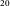 – 10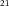 ${\mathrm{cm}}^{-3}$). A comparison of the alloy scattering rates to the IIS and POP rates in a range of high carrier concentrations is shown in Figure S12a–c to demonstrate this assumption. Our objective here is to understand the change in the maximum achievable PF of pristine materials upon alloying. Figure 4 shows the maximum PF at 300, 600, and 900 K for *n*‐type (top) and *p*‐type (bottom) transport, and column‐wise for all the compositions of ${\mathrm{Ti}}_{1-x}{\mathrm{Zr}}_{x}\mathrm{NiSn}$, ${\mathrm{Zr}}_{1-x}{\mathrm{Hf}}_{x}\mathrm{NiSn}$, and ${\mathrm{Hf}}_{1-x}{\mathrm{Ti}}_{x}\mathrm{NiSn}$, respectively. This is typically reached when the Fermi level is around the band edge. The Fermi level dependent PF at 300 K is shown in Figures S13 and S14. Note that in all our simulations we include bipolar transport, irrespective of bandgap and temperature. At high temperatures the effect or bipolarity on the PF at 600 and 900 K is shown for HfNiSn in Figure S15 can have 10% –25% reduction.

In the *n*‐type region shown in Figure 4a–c, the variation in PF is almost linear between the end pristine materials. In case of 300 K, the PFs are around 7–8 mW ${\mathrm{cm}}^{-1}$ $K^{-2}$, whereas they increase to value between 9 and 12 mW ${\mathrm{cm}}^{-1}$ $K^{-2}$ for 600 and 900 K. Overall, it appears that for most compositions the PFs reside at their highest level, with the inclusion of Ti being the factor to reduce the performance toward the lower PF values. The maximum PF reported for a few high performing XNiSn alloys is also shown in Figure 4a–c  [22, 23, 50, 51, 60, 61, 62, 63, 64]. A more systematic comparison of calculated data with experimental data for a series of ${\mathrm{Zr}}_{1-x}{\mathrm{Hf}}_{x}\mathrm{NiSn}$ alloys, but at a fixed carrier concentration is shown in Figure S16  [22]. In general, due to the presence of defects and disorder in these typically polycrystalline systems, the measured Seebeck is somewhat higher that the computed one, but the measured conductivity is around a factor of 2–3 × reduced compared to computed values. Overall the measured PF remains lower than calculated by approximately a factor of 2 × (as also observed for NbFeSb  [37]). Having better control of defects could double the PF in this class of compounds up to the ideal predicted value, as observed in NbFeSb and TiCoSb  [65, 66]. Note that the PF maxima shown are highly dependent on the electronic structure, which changes with alloying. Here, they occur at carrier concentrations that vary between 8 × 10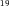 ${\mathrm{cm}}^{-3}$ and 2×10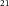 ${\mathrm{cm}}^{-3}$.

For the *p*‐type region shown in Figure 4d–f, a different trend is observed where the temperature variation of the peak PFs is less pronounced, as we also observed for *p*‐type NeFeSb in Ref [37]. The PFs peak at 600 K and drop again for 900 K. This is due to the stronger non‐parabolicity and band‐warping in the VB (compared to the CB), which reduces the conductivity for higher energies that participate in transport at higher temperatures. In contrast to the *n*‐type polarity, however, Ti‐rich compounds seem to have the largest PFs. For 600 K and 900 K the trend is non‐linear with composition, with larger reduction around the middle alloying region due to changes in the electronic structure. As as example, we show the band velocities for TiNiSn, ${\mathrm{Ti}}_{0.5}{\mathrm{Zr}}_{0.5}\mathrm{NiSn}$, and ZrNiSn in Figure S17. While the band velocity is monotonically increasing for *n*‐type going from TiNiSn to ZrNiSn, it is non‐monotonic for *p*‐type. Note also that the bandgap is also changing with alloying as shown in Figure S18. TiNiSn, ZrNiSn and HfNiSn have bandgaps of 0.45, 0.52, and 0.39 eV, respectively. A variation of 25% is d for ${\mathrm{Zr}}_{1-x}{\mathrm{Hf}}_{x}\mathrm{NiSn}$ alloys, which would induce bipolar effects at high temperatures  [56].

### 3.3 Lattice Thermal Conductivity and zT

**FIGURE 5:**
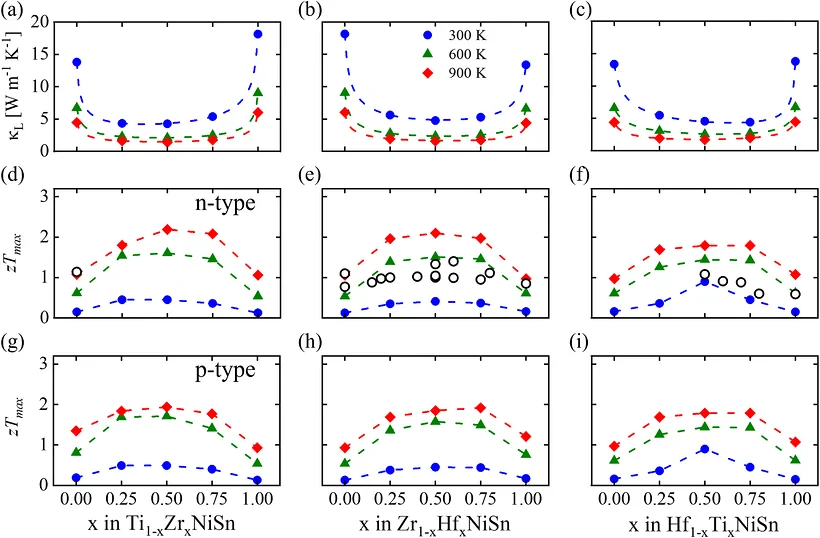
(a–c) Lattice thermal conductivity ($\kappa_{L}$) for 300–900 K, and $zT_{max}$ for: (d–f) *n*‐type, middle row, and (g–i) *p*‐type, third row, versus composition for ${\mathrm{Ti}}_{1-x}{\mathrm{Zr}}_{x}\mathrm{NiSn}$, ${\mathrm{Zr}}_{1-x}{\mathrm{Hf}}_{x}\mathrm{NiSn}$, and ${\mathrm{Hf}}_{1-x}{\mathrm{Ti}}_{x}\mathrm{NiSn}$. Experimental ${\mathrm{zT}}_{max}$ for a few compositions in the range of 300–973 K are shown in open circles.  [22, 23, 50, 51, 60, 61, 62, 63, 64]

We now examine how the alloying will affect $\kappa_{L}$, and then extract the zT. First, we calculate the temperature dependence of $\kappa_{L}$ (from 300 to 900 K) for pristine TiNiSn, ZrNiSn, and HfNiSn using the Phono3Py  [67, 68] package. This uses the finite displacement method to estimate 2^nd^ and 3^rd^ order force constants, as shown in Figure S19. The calculated values are in good agreement with previous reported calculations  [69, 70]. Afterward, we calculate $\kappa_{L}$ in the same temperature range for ${\mathrm{Ti}}_{0.5}{\mathrm{Zr}}_{0.5}\mathrm{NiSn}$, ${\mathrm{Zr}}_{0.5}{\mathrm{Hf}}_{0.5}\mathrm{NiSn}$, and ${\mathrm{Hf}}_{0.5}{\mathrm{Ti}}_{0.5}\mathrm{NiSn}$, as shown in Figure S19. The effect of alloying plays an significant role in the reduction of $\kappa_{L}$. The relaxation time and phonon mean‐free‐path as a function of phonon frequency is shown in Figure S20. The mean‐free‐paths are reduced significantly due to alloying, as it locally changes the bonding. The thermal conductivities at 300 K reduce by around 3×, from 13.8, 18.14, and 13.37 W $m^{-1}$ $K^{-1}$ for TiNiSn, ZrNiSn, and HfNiSn down to 4.25, 4.75, and 5.14 W $m^{-1}$ $K^{-1}$ for ${\mathrm{Ti}}_{0.5}{\mathrm{Zr}}_{0.5}\mathrm{NiSn}$, ${\mathrm{Zr}}_{0.5}{\mathrm{Hf}}_{0.5}\mathrm{NiSn}$, and ${\mathrm{Hf}}_{0.5}{\mathrm{Ti}}_{0.5}\mathrm{NiSn}$, respectively, as shown in Figure 5a–c. In order to estimate the $\kappa_{L}$ for intermediate compositions which are computationally challenging due to large unit cells and low symmetry, we use the Klemens model  [71, 72]. The use of this model is typical for XNiSn and other HH alloys  [18, 73, 74, 75, 76, 77]. The disorder parameter used in the model is estimated by using the accurate value of $\kappa_{L}$ calculated for *x* = 0.5 using Phono3Py. More details, and comparison with experimental values are given in Section S3 and Figure S21.

Having accurate calculations for the PF of these alloyed systems, we can combine them with $\kappa_{L}$ and extract the zT. The electronic part of the thermal conductivity, $\kappa_{e}$, is also calculated from the BTE and accounted in the extraction of zT. It is found to vary between 2–5 W $m^{-1}$ $K^{-1}$ as shown in the Figure S22. Due to the low band gap, $\kappa_{e}$ also has contribution from the bipolar part of the electronic thermal conductivity ($\kappa_{bi}$), which is fully accounted in our formalism, as our energy integration extends from the VB, through the band gap and into the CB on the same level (more details on $\kappa_{bi}$ are given in the Supporting Information). Figure 5d–f,g–i shows the maximum calculated zT for a few compositions for *n*‐type and *p*‐type materials, respectively. Results for 300, 600, and 900 K are shown. Since the band structure and PF does not change significantly with alloying, the reduction in $\kappa_{L}$ in the alloyed cases (middle points in Figure 5a,b,c) leads to a significant enhancement in the maximum achievable zT for the *x* = 0.5 compositions. Values just short of zT=2 can be achieved at 600 K and beyond. XNiSn type of HHs are intrinsically n‐type and most reports on high achieved zT values are indeed for n‐type materials, as shown in the Figure 5d,e,f, while further comparisons for a series of ${\mathrm{Zr}}_{1-x}{\mathrm{Hf}}_{x}{\mathrm{NiSn}}_{0.985}{\mathrm{Sb}}_{0.015}$ composition are is shown in Figure S16d. Note that most of these measured zT values are almost 2× lower compared to the theoretically idealized value. This is because, although the electrical conductivity and PF do not suffer from alloying, they suffer significantly in a realistic scenario from polycrystallinity, intrinsic defects, grain boundaries, strain etc. On the other hand the the thermal conductivity is already reduced from alloying, thus defects have less of an effect. The magnitude of the PF reduction depends on the type of dopant and the quality of polycrystalline sample, but our simulations here indicate the ultimate limit that can be reached under ideal synthesis conditions for defect‐free alloyed samples, somewhere at the middle of the alloyed composition. Under these conditions, the achievable zT can be as high as double compared to what is typically realized in experiment.

## 4 Transport Properties of XFeSb (X = Ta/Nb) Alloys

In the materials discussed in the previous section, the change in the band structure is not significant, with the degeneracy of the bands remaining intact after alloying, in both the valence and conduction bands. This is not always the case. Here, we consider the transport properties of TaFeSb alloyed with NbFeSb (Nb doping at Ta). In this case the conduction band changes significantly. We also consider a few aliovalent substitutions at the Ta site, which induce changes in the VB. Importantly, although being one of the best HH thermoelectric materials  [78], the transport properties of pristine TaFeSb have not yet been fully explored, which is what we provide initially here as well.

### 4.1 Thermoelectric PF Transport Properties of Pristine TaFeSb

**FIGURE 6:**
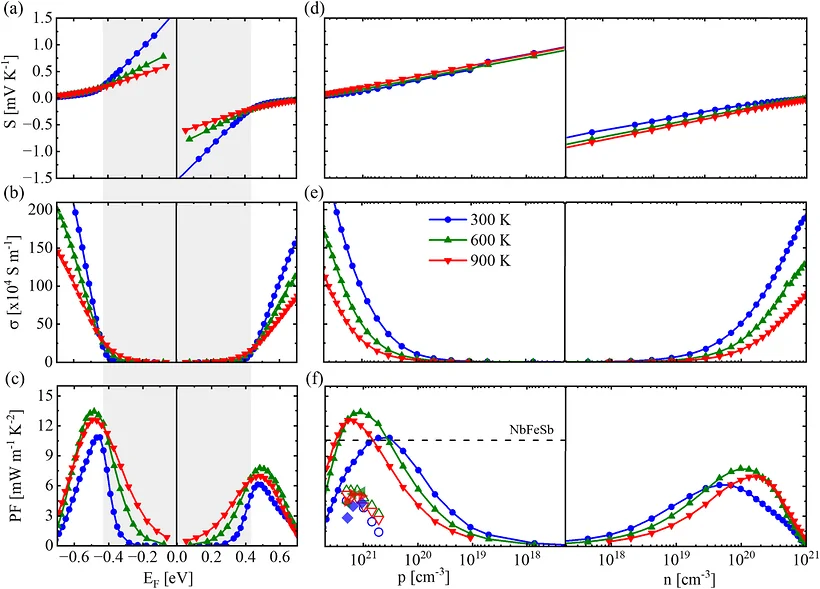
Calculated thermoelectric coefficients for pristine TaFeSb: (a, d) Seebeck coefficient, (b, e) electrical conductivity and (c, f) power factor. The left panel shows results versus the position of the Fermi level, E_F_, whereas the right panel versus doping density. Experimental values/level for the PF of TaFeSb and NbFeSb are from Refs.  [78, 79, 80].

**FIGURE 7:**
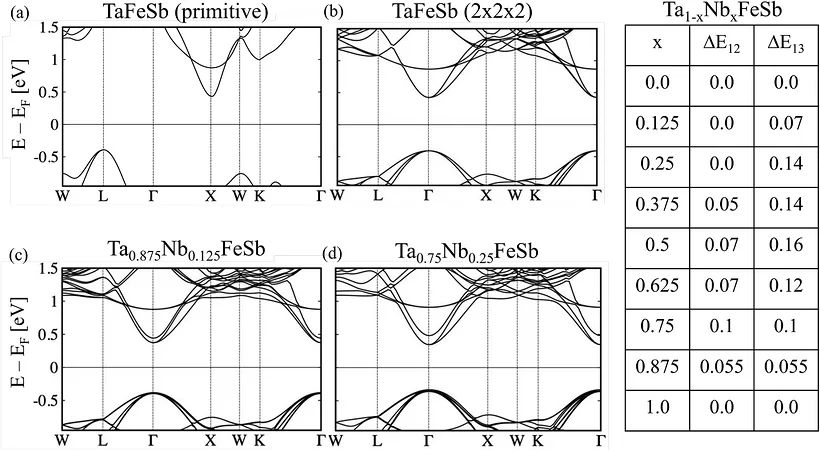
Band structure of TaFeSb of: (a) the primitive cell, (b) the 2×2×2 supercell. (c,d) Band structures of ${\mathrm{Ta}}_{0.875}{\mathrm{Nb}}_{0.125}\mathrm{FeSb}$ and ${\mathrm{Ta}}_{0.875}{\mathrm{Nb}}_{0.125}\mathrm{FeSb}$. The table in the right shows the energy difference between the three conduction bands, where $\Delta E_{12}$ and $\Delta E_{13}$ are the energy gap between first‐second and first‐third band, respectively, for all compositions considered (*x* = 0.0–1.0).

To begin with, we calculate the TE coefficients of pristine TaFeSb with respect to the Fermi level's relative position, E_F_, which correlates directly with doping density. Here, E_F_ is set to 0 eV at the mid gap. The VB maxima and CB minima are located at ‐0.43 and 0.43 eV respectively. All relevant scattering mechanisms (ADP, ODP, POP, and IIS) are considered, and all input parameters to the code, such as deformation potentials, dielectric constants, and sound velocities, are calculated ab initio. The thermoelectric transport coefficients (S, σ and PF = $S^{2}\sigma$), versus the relative position of the Fermi level E_F_ (left‐panel), as well as versus the doping density (right‐panel) for different temperatures, are shown in Figure 6. Since the bandgap is rather large at 0.86 eV, we consider unipolar transport for the VB and CB separately. The gray shaded region indicates the band gap. In the *p*‐type region (left sides) the PF peaks at 10.8 ${\mathrm{mWm}}^{-1}K^{-2}$ at 300 K (at a density of $p=5.3\;\times \;10^{20}\;{\text{cm}}^{-3}$). The PF increases to 13.43 ${\mathrm{mWm}}^{-1}K^{-2}$ at 600 K (at $p=1.12\;\times \;10^{21}\;{\text{cm}}^{-3}$), and then slightly reduces to 12.72 ${\mathrm{mWm}}^{-1}K^{-2}$ at 900 K (at $p=1.91\;\times \;10^{21}\;{\text{cm}}^{-3}$). This is a consequence of the two L‐valley bands (see band structure in Figure 7a), resulting in a significant 8‐fold degenerate valleys, in a similar manner to NbFeSb. Note that in this case of TaFeSb the calclation followed the exact same recipe and steps as we have previously presented for NbFeSb in Ref.  [37].

The *n*‐type performance is similar to most HHs, which have a single band at the X‐point with three‐fold degeneracy  [37, 38]. A PF maximum of 6.12 ${\mathrm{mWm}}^{-1}K^{-2}$ at 300 K (at n = 4.6×10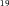 ${\mathrm{cm}}^{-3}$) is observed, which increases slightly to 7.76 ${\mathrm{mWm}}^{-1}K^{-2}$ at 600 K (n = 9.8×10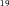 ${\mathrm{cm}}^{-3}$), and then down to 6.97 ${\mathrm{mWm}}^{-1}K^{-2}$ at 900 K (n = 1.7×10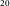 ${\mathrm{cm}}^{-3}$). The *p*‐type polarity has a higher PF in general, which appears at higher densities as well (almost an order of magnitude higher). This is due to the higher VB valley degeneracy compared to the CB (eight‐fold vs. three‐fold) and, thus, the higher density needed to place the Fermi level at its optimal position at the band edge. Note that it could be conceivable that the degenerate bands at the L‐point would result in inter‐band scattering and reduce the conductivity and PF. We have shown, however, that in the case of degenerate bands as these, the overlap integrals forbid strong inter‐valley/band scattering, and the PF remains high  [37, 38].

Experimental data for *p*‐type TaFeSb materials are also shown in Figure 6f  [78, 79, 80] (scatter plots). The PF is around half of the calculated PFs, which is standard behavior in comparisons of simulation versus experiment in TE materials  [37, 38, 55]. The mismatch typically originates from disorder and other non‐uniformities in the measured data, that are not captured/included in simulations, and/or from alloying effects in experiment that we do not consider in this particular pristine material calculation. A full comparison with experimental reported Seebeck coefficient, conductivity and PF for ${\mathrm{Ta}}_{1-x}{\mathrm{Ti}}_{x}\mathrm{FeSb}$ is shown in Figure S23a–c. As in the case of the XNiSn system above, the experimental Seebeck coefficient in this case is also higher than calculated and the experimental conductivity is significantly lower. The experimentally synthesized ${\mathrm{Ta}}_{1-x}{\mathrm{Ti}}_{x}\mathrm{FeSb}$ is reported to have Ta/Ti‐Fe antisite disorder, and along with grain boundaries would be the reason behind such disagreement  [80]. Also, Ti substitution will lead to band splitting, which reduces the conductivity. Overall, the experimental maximum PF is around half of the computational values (as expected). The zT calculation and its comparison with the highest reported zT for TaFeSb‐based HH is shown in Figures S23d,e. Because of drastic reduction in $\kappa_{L}$ in ${\mathrm{Ta}}_{1-x}{\mathrm{Ti}}_{x}\mathrm{FeSb}$ (Figure S23d), the experimental zT comes out to be even higher than the calculated one for the ideal (but pristine) TaFeSb system (Figure S23e). The reduction in the PF because of disorder and defects, reduced the PF by 2–3×, but the reduction in lattice thermal conductivity is much more (here the thermal conductivity is higher compared to the XNiSn systems, thus suffers relatively more from both alloying and defects. The change in the band structure and the PF due to doping and alloying will be addressed in the next section. Specifically, we will examine the effect of isovalent and aliovalent doping on the band structure and PF.

### 4.2 Effect of Isovalent (Nb) Alloying on the PF of TaFeSb

We first examine isovalent alloying of Nb in the place of Ta in TaFeSb. We consider a 2×2×2 supercell (24‐atom) of the pristine HH structure (3‐atom). Note that earlier for Si we used an 8 atom supercell, for the XNiSn system we used a 12 atom supercell (either cubic and orthorhombic), because we only considered three alloying concentrations for *x* = 0.25, 0.5, and 0.75. Here we use a 24 atom supercell, because we use more alloying concentrations. The Ta atoms are randomly replaced by Nb atoms to make alloyed compositions. There are many possibilities of Ta‐Nb randomly occupying the same lattice cite in a 24‐atom supercell. We use the special‐quasirandom structure (SQS) generator for each composition to obtain random structures  [81]. Figure 7a,b shows the band structure of the primitive cell and the supercell. The 3‐fold degenerate CB at the X‐points of the primitive BZ is folded to the Γ‐point of the supercell BZ. The VB maxima at the L‐points consist of 2 bands, making it eight‐fold degenerate, and all these are also folded on the Γ‐point. Figure 7c,d shows the change in the band structure after alloying 12.5% and 25% Nb. Significant changes can be observed in the CB, where the degeneracy is lifted due to alloying. The energy separation between the three bands as a function of Nb‐alloying, is shown in the table to the right of Figure 7 (from the first to the second, $\Delta E_{12}$, and from the first to the third band, $\Delta E_{13}$). The Ta atom contributes more to the CB  [82] as shown by the atomic resolved projected band structure in Figure S24, thus it is likely that replacing it with Nb will result in larger changes there. The loss in degeneracy can also be understood as the loss in the crystal symmetry. A foreign element into the matrix reduces the crystal symmetry leading to a modified irreducible Brillouin zone (BZ). The equivalent X‐points at the BZ become inequivalent and susceptible to loss in degeneracy. However, with reduced symmetry after alloying, the extent of degeneracy loss depends on the difference in the bonding of the original and alloyed element (in this case Ta and Nb) in the matrix, which would depend on various factors, such as atom size and electronegativity difference between the alloyed elements. In this case, there is no size effect as both Nb and Ta have the same ionic radius, and thus the lattice parameter does not change with alloying, but we observed that the splitting follows the alloying concentration as x(1‐x) (see Figure S25a and relevant discussions for the correlation trend). Unlike the CB, the VB does not undergo any such band splittings, as its majority contribution is from Fe atoms as shown in Figure S24. Note that we also verified the dynamical stability of this alloyed composition by the absence of imaginary modes in Figure S26.

We now examine the effect of alloying and band structure changes on electronic and TE transport for *n*‐type materials, as the alloying effect is more prominent in the CB. Before that, there is a subtle point that we need to clarify. The most significant scattering mechanisms IIS and POP, are anisotropic, with the scattering strength depending strongly on the k‐space distance between the initial and final states in the BZ. While the folded supercell provides a reasonable alloyed electronic structure, the price to be paid is loss in the k‐space placement information. Thus, since the different folded valleys are in reality placed farther from each other at different edges of the BZ, rather than the center, they essentially contribute independently to transport without inter‐valley scattering from IIS and POP. Inter‐valley scattering is also not applicable for ADP scattering which is intra‐valley due to the small phonon momenta involved. ODP can still allow inter‐valley scattering and we consider this. We implement these scattering considerations in our BTE code ElecTra  [33].

**FIGURE 8:**
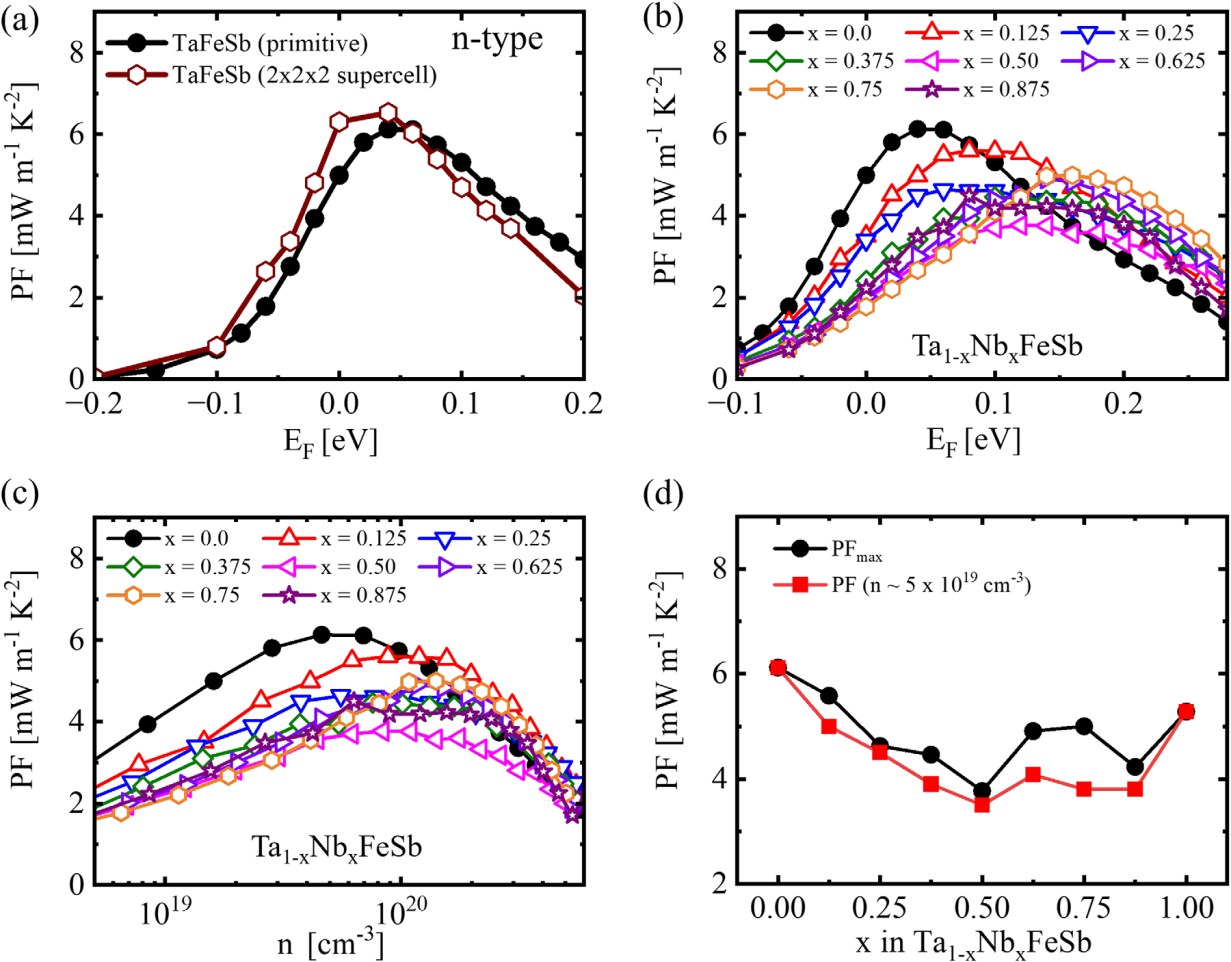
(a) Comparison of the PF of pristine TaFeSb, calculated on the primitive unit cell and on the supercell structure. (b,c) PF as a function of Fermi energy and carrier concentration for all compositions‐x of ${\mathrm{Ta}}_{1-x}{\mathrm{Nb}}_{x}\mathrm{FeSb}$. (c) Maximum power factor (${\mathrm{PF}}_{max}$) and PF at fixed density (5 × 10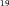 ${\mathrm{cm}}^{-3}$) as a function of composition.

We verify our approach by comparing the PFs of the pristine TaFeSb primitive versus those of the supercell, as shown in Figure 8a. The slight mismatch between the pristine (black line) and supercell (red line) PFs, is due to the mixing of the Fermi surfaces in the supercell electronic structure calculation, a consequence of the bands not being perfectly parabolic (unlike earlier where we used an orthorhombic unit cell to avoid crossings, here for this larger unit cell we don't have this, thus bands fold onto the Γ‐point and some mixing occurs). The associated error in the PF with choice of supercell is shown in Figure S6d–f. We also calculated all the alloyed system parameters needed as inputs to the transport calculations. As in the previous systems, we find that the calculated parameters, such as sound velocity and dielectric constants, follow a linear change with composition *x* in ${\mathrm{Ta}}_{1-x}{\mathrm{Nb}}_{x}\mathrm{FeSb}$ as shown in Table S3. Therefore, we also assume a linear change in the deformation potentials for phonon scattering as well. All the parameters we use are listed in Table S3. Figure 8b,c shows the PF as a function of the Fermi energy and carrier concentration, respectively. The PF maxima in the alloyed system is observed at different Fermi energies, but with respect to carrier concentration they all fall between n = 5×10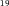 and 2×10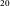 ${\mathrm{cm}}^{-3}$ as shown in Figure 8c. Figure 8d shows the maximum PF of the different alloys, and the PF at n = 5×10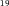, the carrier concentration at which PF is maximum for pristine TaFeSb and NbFeSb  [37]. For *x* = 0.5 (magenta line), the maximum alloyed case, the ${\mathrm{PF}}_{max}$ decreases by almost 50% compared to the PF of the pristine TaFeSb (black line), down to 4 mW ${\mathrm{cm}}^{-1}$ $K^{-2}$.

Figure S27a–c shows the PF, Seebeck coefficient, and electrical conductivity versus Fermi level, respectively, for *x* = 0 and 0.5. Our calculation shows the reduction in the PF is a consequence of reduction in conductivity. A direct measure of conductivity is the TDF (Equation 12), which accounts for the combined effect of scattering rates, band velocities and density of states. Figure S27d shows that for *x* = 0.5, where the bands are split, the TDF decreases, which is reflected in the reduced electrical conductivity. In summary, the loss in degeneracy is the reason for reduction in the maximum TE PF upon alloying, as it leads to reductions in the TDF around those low energy values. In fact, we have also shown this using a simple parabolic band model  [83, 84]. When two bands are split by 0.2 eV, a reduction of 20%–60% in PF is observed depending on the effective mass of the bands and various scattering considerations. Even with the reduced PF in the alloyed composition, these alloys will typically have higher zT compared to pristine materials, because of the larger reduction in $\kappa_{L}$. Figure S28 shows a 3× reduction of $\kappa_{L}$ due to mass disorder in ${\mathrm{Ta}}_{1-x}{\mathrm{Nb}}_{x}\mathrm{FeSb}$.

### 4.3 Effect of Aliovalent Substitution in the PF of TaFeSb

**FIGURE 9:**
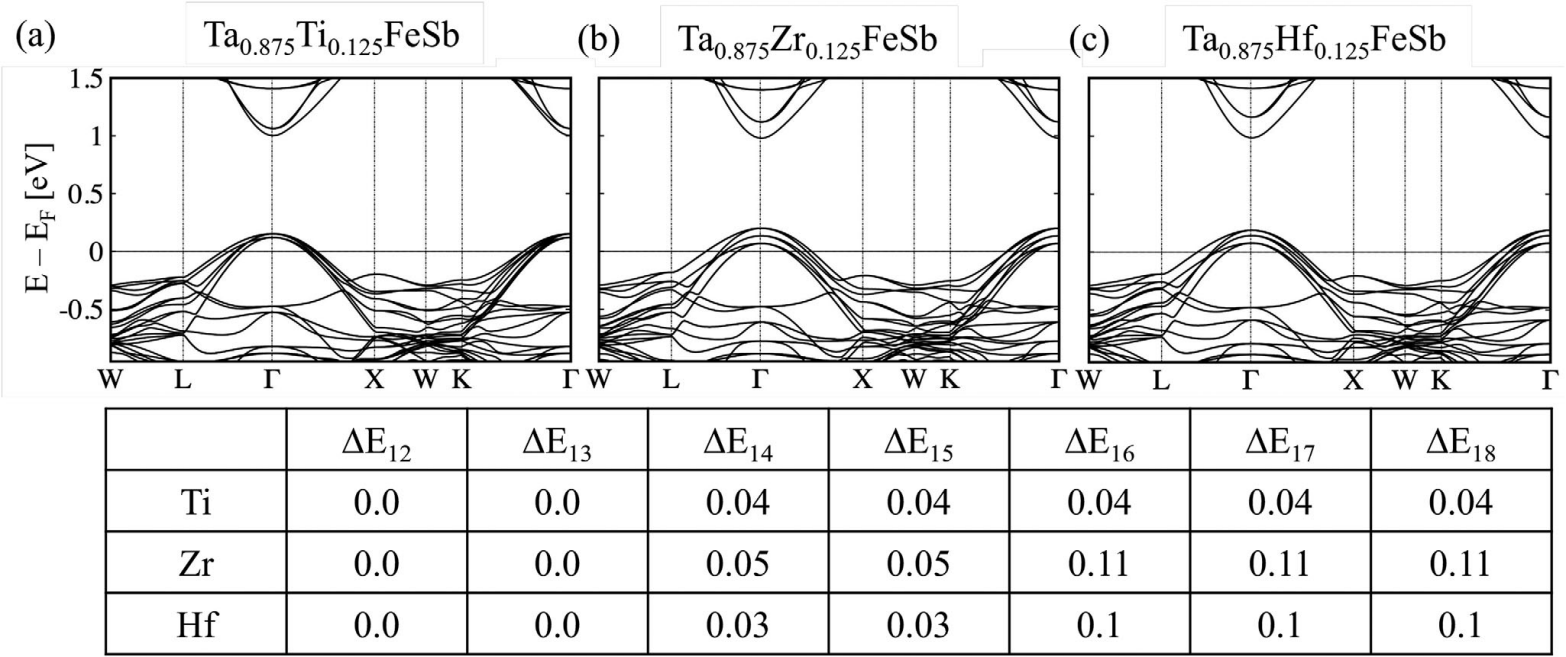
Band structure of: (a) ${\mathrm{Ta}}_{0.875}{\mathrm{Ti}}_{0.125}\mathrm{FeSb}$, (b) ${\mathrm{Ta}}_{0.875}{\mathrm{Zr}}_{0.125}\mathrm{FeSb}$, and (c) ${\mathrm{Ta}}_{0.875}{\mathrm{Hf}}_{0.125}\mathrm{FeSb}$. The table at the bottom shows the energy difference between the eight valence bands, where $\Delta E_{12}$, $\Delta E_{13}$ and so on, are the energy gaps between the first‐second, first‐third bands and so on, respectively.

In order to achieve the optimum carrier concentration, the most practical approach is aliovalent substitution. Here, we investigate the doping of Ti, Zr, and Hf at the Ta site of TaFeSb and how transport properties might be altered. Ti doped TaFeSb/NbFeSb is reported to have excellent p‐type thermoelectric properties  [10, 78, 80, 85, 86]. Figure 9a–c shows the electronic structure of 12.5% Ti, Zr, and Hf doped TaFeSb respectively. Although Ta contributes mostly in the CB, doping Ti/Zr/Hf having one less electron will change the bonding with Fe, which can split the VB as well. Here, the extent of band splitting depends on the size difference between the host and dopant atoms. The splitting is largest in the case of Zr, because of its larger size compared to Ta. Hf, being sightly smaller than Zr, splits the bands similarly qualitatively, but somewhat less. A linear correlation of size difference and splitting values is shown in Figure S25b. Thus, although at this point we cannot establish a full mapping between physical effects and valley splittings for most systems a prior, we have indications that the atomic size difference could be a strong indication. The valley energy positions of the seven higher energy bands in the VB, compared to the top VB maximum (the first band) are tabulated in Figure 9 in the Table at the bottom.

Transport treatment in the VB case is more complicated compared to the CB earlier. While the CB has single degenerate valleys, and when folder on the same BZ point, they can still be treated independently of each other (with intra‐band scattering only). VB is more complex, as it has two two bands at the same valley (L‐point). In the pristine unit cell BZ, the overlap integral ($\left\langle I_{kk^{\prime }}^{2}\right\rangle$) is calculated to be 0.5 for both intra‐ and inter‐band transitions  [37, 38]. However, when a supercell of 2×2×2 is created, the two bands at the L‐point, get folded onto a same Γ‐point as shown in Figure S29a,b (L valley is four‐fold degenerate). Now, the situation is complex and simplification is difficult. Intra‐band scattering can be implemented with $\left\langle I_{kk^{\prime }}^{2}\right\rangle$ = 0.5. But inter‐band scattering has to be implemented on these bands pair‐wise, i.e. from each band transitions into only one other band transition is allowed with $\left\langle I_{kk^{\prime }}^{2}\right\rangle$ = 0.5, whereas all other inter‐band transitions are weak (as in the pristine material ‐ these valleys are farther away from each other) and should not be considered. With eight bands mixed together, it is impossible to identify and implement such pair‐wise scattering. The reason is fundamental to DFT calculation and how band numbers are assigned based on energy. So, instead of implementing pairwise inter‐band scattering we simplify the treatment using $\left\langle I_{kk^{\prime }}^{2}\right\rangle$ = 1 (for intra‐band) and 0 (for inter‐band) for all bands (i.e., each bands scatters fully into one band ‐ its own band, rather than with half strength in two bands). With this assumption the PF calculated on 2×2×2 supercell is shown in Figure S29c, which leads to a 23% error, due to unavoidable mixing of the 8 bands at same Γ‐point. So far we could not find any other transformation matrix to avoid band mixing in this case, as we did for the for XNiSn system by choosing the orthorhombic supercell. With Ti/Zr/Hf alloying the PF reduces as shown in Figure S29d. Since the absolute value of the PF has an error of 23%, we discuss the relative change in the PF with Ti/Zr/Hf alloying, rather than absolute numbers here below.

**FIGURE 10:**
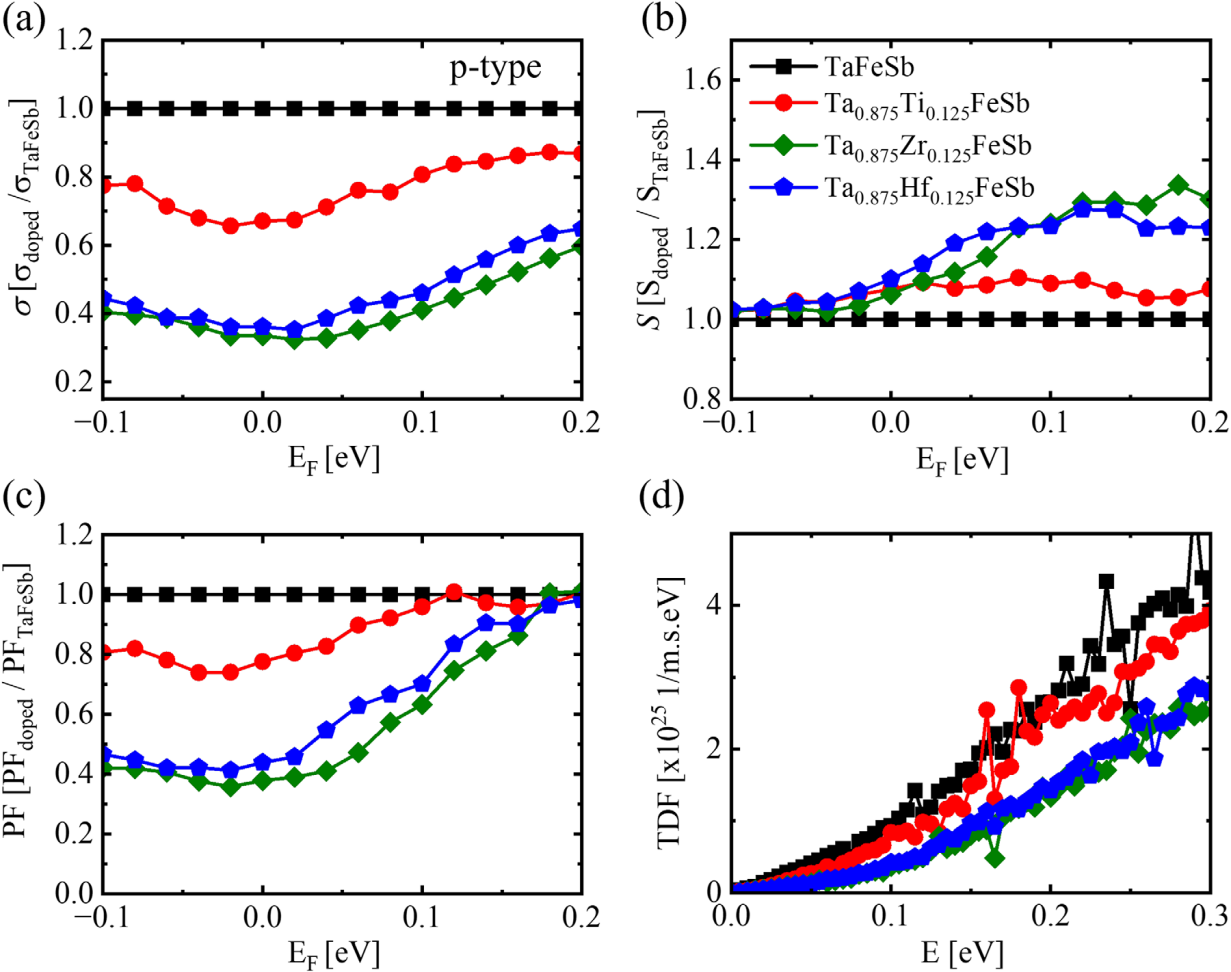
Relative change of: (a) electrical conductivity, (b) Seebeck coefficient, and (c) PF as a function of Fermi energy for Ti (red), Zr (green), and Hf (blue) doped TaFesb with respect to pristine TaFeSb (black). (d) Comparison between the Transport Distribution Functions (TDF) versus carrier energy for the four material cases.

Figure 10a shows a relative comparison of alloy material conductivity, σ, with respect to pristine TaFeSb. A large reduction in σ is observed in the alloy cases, especially in the case of Zr (${\mathrm{Ta}}_{0.875}{\mathrm{Zr}}_{0.125}\mathrm{FeSb}$) and Hf alloying (green‐diamond and blue‐pentagon lines), where σ is reduced down to 20% of the pristine at the band edge (E_F_ = 0 eV). For these materials, the splitting in the bands is the largest, which reduces the DOS, carrier velocities (Figure S30a,b), and the Transport Distribution Function (TDF) as well, as shown in Figure 10d. There the TDF is plotted for E_F_ = 0 eV, and directly determines σ. For Ti‐alloying, in which case the splitting is the lowest, the decrease is around half, down to 60% of the pristine value (red‐circle‐point‐line). Upon valley splitting, all quantities that determine the TDF (velocity, DOS, scattering times), contribute to its reduction. Doping not only splits the bands, but it also increases the band's effective mass and decreases velocities (see Table S4). This also reduces the scattering times, especially for Zr and Hf doping (see Figure S30c).

Figure 10b shows a relative comparison of the alloy materials' S with respect to pristine TaFeSb. Around the band edge (where the PF peak is typically found), all three alloyed materials experience increase in S by about 20%, following the expected inverse trend compared to σ. Beyond that energy, the Zr and Hf alloys, which have the largest splitting, have an even larger increase in S (blue/green lines). Note that this comparison is performed at a fixed Fermi level, and has to be linked to the conductivity for the trends to make sense (Figure S32 shows an absolute comparison for S as a function of carrier density where in that case S is lowest for Zr doping). The reason for the increase in S at a fixed Fermi level upon band splitting is the fact that transport suffers at the energies around the band edges as the global average velocities suffer. This shifts the energy of the current flow to higher energies, and increases S.

This behavior, overall leads to a large (50%) reduction in the PF in case of Zr and Hf, around E_F_ = 0 eV (band edge) as shown in Figure 10c. In general, these results show that compounds with high valley degeneracy (typically at the BZ edges), are more susceptible to band structure changes with alloying/doping. As shown in this case, doping with Ti, Zr, and Hf, influence the band differently, leading to differences in conductivity and PF. The atomic size and (possibly) electronegativity of the foreign element are the major factors that affect degeneracy lifting, a major PF degradation mechanism, as we have shown. Thus, although many details regarding the chemistry of each dopant are not considered in the calculation, our results show the importance of the doping element choice in order to achieve optimum PF. A similar trend is observed experimentally in NbFeSb which is analogous to TaFeSb. Among Ti, Zr, and Hf doped NbFeSb, Zr doping shows the lowest PF  [87]. Note that due to the discrepancy of band mixing and the corresponding error in this material, we further used an eight‐band parabolic effective mass model (with band edges and masses in Table S4) to validate the transport coefficients and findings, which shows similar factor of reduction in the PF as in the Ti/Zr/Hf alloying case (Figure S31).

## 5 Conclusions

In conclusion, this work presented an efficient ab initio method to calculate thermoelectric transport properties in alloyed structures. To incorporate alloying, supercell approaches were used, which led to band folding, band splitting, and broken degeneracies, posing significant challenges for Boltzmann transport (BTE) calculations. Using the proposed approach, these challenges were addressed without additional computational cost.

The method was first tested on the well‐studied Si–Ge alloy system, where it reproduced the low‐field mobility trends as a function of Ge content. We found that considering alloying through the electronic structure of supercells, accounts for the effect of alloying on transport only partially, around 50%. An additional alloy scattering rate term is required to match experimental mobility. This term empirically captures the effect of atomic‐scale disorder that cannot be represented within finite simulation cells. At higher carrier concentrations, relevant for thermoelectric operation, alloy scattering was found to be dominated by ionized impurity scattering, indicating a limited role under optimal conditions.

The method was then applied to alloyed half‐Heusler systems ${\mathrm{Ti}}_{1-x}{\mathrm{Zr}}_{x}\mathrm{NiSn}$, ${\mathrm{Zr}}_{1-x}{\mathrm{Hf}}_{x}\mathrm{NiSn}$, and ${\mathrm{Hf}}_{1-x}{\mathrm{Ti}}_{x}\mathrm{NiSn}$ (*x* = 0.0, 0.25, 0.50, 0.75, 1.0). Alloying affected the band curvature in terms of effective mass, while band degeneracy at high‐symmetry points remained preserved. Transport calculations showed consistent variation in the power factor with composition, particularly at 300 K. The reduction in lattice thermal conductivity emerged as the dominant factor enabling high thermoelectric performance (zT≈2). In a second class of materials, TaFeSb‐type half‐Heuslers with isovalent (Nb) and aliovalent (Ti/Zr/Hf) alloying were examined. These systems, characterized by zone‐edge valleys, exhibited significant changes in band structure upon alloying. In this case, alloying led to band splitting and a reduction in peak power factor of up to 50%. This indicates that while multi‐valley materials can offer enhanced transport properties, they are more susceptible to disorder‐induced degradation, resulting in a lower maximum achievable power factor compared to the pristine compounds.

Overall, we observe the following regarding power factors (PFs) and zT: Pristine non‐alloyed materials have the highest PF, but lowest zT due to high thermal conductivity. From pristine materials to alloys, typically a small reduction is observed in the PF, up to a factor of 50% in special cases of multi‐off‐zone‐center valleys (this is the idealized computational case, where no further defects are assumed). The thermal conductivity, however, suffers more, by up to 3×, which allows for a factor of around 2× improvement in zT (which turns out to be the best performing case with zT approaching 2). In reality, however, defects such as grain boundaries, vacancies, disorder, etc, typically reduce the PF by a factor of 2×, dragging the zT somewhat lower, despite further reductions in the thermal conductivity as well. Typically, we observe that the electrical conductivity is a factor of 3× higher in calculations compared to experiment, while the Seebeck coefficient somewhat lower, resulting in PFs of 2× higher in calculations. For practical purposes, at the same zT a material with higher PF (defect‐free alloy) would be preferred.

## 6 Computational Methods

The electronic band structure, phonon dispersion, and electron‐phonon coupling matrix elements are determined using DFT and DFPT through the Quantum ESPRESSO package  [88, 89]. The calculations employ optimized norm‐conserving Vanderbilt (ONCV) pseudopotentials within the framework of the generalized gradient approximation (GGA) utilizing the Perdew–Burke–Ernzerhof (PBE) functional  [90]. The EPW package is utilized to carry out Wannier function interpolation for the electron‐phonon coupling matrix elements  [47, 91]. For electronic transport calculations, a denser Monkhorst‐Pack k‐point mesh of 80 × 80 × 80 was used  [92]. The sound velocities are calculated using ${\mathrm{Thermo}}_{\mathrm{PW}}$ package  [93]. We use a special quasi‐random structure (SQS) generated using the mcsqs code (a part of the ATAT toolkit) to obtain random structures of alloys  [81]. Phonon dispersion is calculated using Phonopy package which uses finite displacement to calculate the dynamical matrix  [67, 68]. The lattice thermal conductivity is calculated by solving the phonon BTE in Phono3py, which uses the second order force constants and third order force constant (anharmonicity) of the system  [67, 68].

We use our BTE simulator ElecTra for calculating the transport properties  [33]. The scattering rate expressions for different scattering processes used are as follows. For acoustic deformation potential (ADP):

(1) $$|S_{k,k^{\prime }}^{\text{ADP}}|=\frac{\pi }{\hbar }D_{\text{ADP}}^{2}\frac{k_{B}T}{\rho v_{s}^{2}}g_{k^{\prime }}\left(E\right)$$

where k and $k^{\prime }$ are the initial and final state of carriers, ρ is the mass density, $v_{s}$ is the average sound velocity, and g(E) is the density of states of the final scattering state.

For optical deformation potential (ODP):

(2) $$|S_{k,k^{\prime }}^{\text{ODP}}|=\frac{\pi D_{\text{ODP}}^{2}}{2\rho \omega }\left(N_{\omega }+\frac{1}{2}\mp \frac{1}{2}\right)g_{k^{\prime }}\left(E\pm \hbar \omega \right)$$

where ω is the dominant frequency of the optical phonons, considered to be constant over the entire reciprocal unit cell. $N_{\omega }$ is the phonon Bose–Einstein statistical distribution and the + and ‐ signs indicate the emission and absorption processes.

For inter‐valley scattering (IVS), a similar expression as ODP is used:

(3) $$|S_{k,k^{\prime }}^{\text{IVS}}|=\frac{\pi D_{\text{IVS}}^{2}}{2\rho \omega }\left(N_{\omega }+\frac{1}{2}\mp \frac{1}{2}\right)g_{k^{\prime }}\left(E\pm \hbar \omega \right)$$

where ω is the phonon frequency associated with the corresponding inter‐valley scattering processes.

For polar optical phonon (POP) scattering, we use the Fröhlich formalism as:

(4) $$\begin{array}{ccc} |S_{k,k^{\prime }}^{\text{POP}}| & = & \frac{\pi e^{2}\omega }{\varepsilon_{0}}\frac{|k-k^{\prime }{|}^{2}}{(|k-k^{\prime }{{|}^{2}+\frac{1}{L_{D}^{2}})}^{2}}\left(\frac{1}{k_{\infty }}-\frac{1}{k_{0}}\right)\\ & & \times \;\left(N_{\omega }+\frac{1}{2}\mp \frac{1}{2}\right)g_{k^{\prime }}\left(E\pm \hbar \omega \right)<I_{k,k^{\prime }}^{2}> \end{array}$$

where e is the electronic charge, and ω is the dominant frequency of polar optical phonons over the whole Brillouin zone, which has been validated to be a satisfactory approximation [55]. $k_{0}$ is the static dielectric constant and $k_{\infty }$ is the high‐frequency dielectric constant and $L_{D}$ is the screening length for POP given by $L_{D}=\sqrt{\frac{k_{\infty }\varepsilon_{0}}{e}{\left(\frac{\partial n}{\partial E_{F}}\right)}^{-1}}$. Here, $I_{k,k^{\prime }}$ is the overlap integral, which is given by:

(5) $$I_{mn}\left(k,k+q\right)\;=\;\langle u_{m,k+q}|u_{n,k}\rangle ,$$

and is computed directly from the DFT wave functions stored in the Quantum ESPRESSO output.

For ionized impurity scattering (IIS), we use the Brooks‐Herring model as:

(6) $$|S_{k,k^{\prime }}^{\text{IIS}}|=\frac{2\pi }{\hbar }\frac{Z^{2}e^{4}}{k_{0}^{2}\varepsilon_{0}^{2}}\frac{N_{\text{imp}}}{(|k-k^{\prime }{{|}^{2}+\frac{1}{L_{D}^{2}})}^{2}}g_{k^{\prime }}\left(E\right)<I_{k,k^{\prime }}^{2}>$$

where Z is the electric charge of the ionized impurity (we assume Z = 1 here), $N_{imp}$ is the density of the ionized impurities, and $L_{D}$ is the generalized screening length given by $L_{D}=\sqrt{\frac{k_{0}\varepsilon_{0}}{e}{\left(\frac{\partial n}{\partial E_{F}}\right)}^{-1}}$, where $E_{F}$ is the Fermi‐level and *n* is the carrier density.

The alloy scattering rate (S ^alloy^) is given by  [94]:

(7) $$|S_{k,k^{\prime }}^{\text{alloy}}|=\frac{2\pi }{\hbar }\Omega_{c}\;x\left(1-x\right)G_{k-k^{\prime }}\Delta E_{g}^{2}\;g_{k^{\prime }}<I_{k,k^{\prime }}^{2}>$$

where $\Omega_{c}$ is the volume of the cell, *x* is the alloying fraction and $\Delta E_{g}$ is the scattering potential. The function $G_{k-k^{\prime }}$ is the form factor of hard sphere represented as:

(8) $$G_{k,k^{\prime }}=3\frac{sin\left(qR_{a}\right)-\left(qR_{a}\right)cos(\left(qR_{a}\right)}{{\left(qR_{a}\right)}^{3}}$$

where $q=k-k^{\prime }$ and $R_{a}$ is the radius of a hard sphere with volume equal to cell's volume.

The overall transition rate, $|S_{k,k^{\prime }}|$, is calculated by combining the strength of all scattering mechanisms using Matthiessen's rule as:

(9) $$|S_{k,k^{\prime }}\left|=\right|S_{k,k^{\prime }}^{\text{ADP}}\left|+\right|S_{k,k^{\prime }}^{\text{ODP}}\left|+\right|S_{k,k^{\prime }}^{\text{IVS}}\left|+\right|S_{k,k^{\prime }}^{\text{POP}}\left|+\right|S_{k,k^{\prime }}^{\text{IIS}}\left|+\right|S_{k,k^{\prime }}^{\text{alloy}}|$$

After calculating the transition rates, the thermoelectric coefficients, namely the electrical conductivity, σ, and Seebeck coefficient, S, are calculated as:

(10) $$\sigma_{ij}=e^{2}\int_{E}\Xi_{ij}\left(E\right)\left(-\frac{\partial f_{0}}{\partial E}\right)dE$$

(11) $$S_{ij}=\frac{ek_{B}}{\sigma_{ij}}\int_{E}\Xi_{ij}\left(E\right)\left(-\frac{\partial f_{0}}{\partial E}\right)\frac{E-E_{F}}{k_{B}T}dE$$

Here, $f_{0}$ is the equilibrium Fermi–Dirac distribution function, $\Xi_{ij}\left(E\right)$ is transport distribution function (TDF) defined as:

(12) $$\Xi_{ij}\left(E\right)=\int_{E}\tau_{k,k^{\prime }}\left(E\right)v_{ij}^{2}\left(E\right)g\left(E\right)$$

where, v(E) is the band structure velocity, g(E) is the density of states at energy E, and i,j are the Cartesian coordinate indexes, (for which we set i=j=x) and $\tau_{k,k^{\prime }}$ is the scattering relaxation time (inversely proportional to the transition rates), which is calculated for each scattering mechanism ($m_{s}^{\prime }$) as:

(13) $$\tau_{k,k^{\prime }}=\frac{1}{{\left(2\pi \right)}^{3}}\sum_{k^{\prime }}\left|S_{k,k^{\prime }}^{m_{s}^{\prime }}\right|\left(1-\frac{v_{\left(k^{\prime },n^{\prime }\right)}}{v_{\left(k,n\right)}}\right).$$

## Conflicts of Interest

The authors declare no conflicts of interest.

## Supporting information


**Supporting File**: advs77630‐sup‐0001‐SuppMat.pdf.

## Data Availability

The data that support the findings of this study are available from the corresponding author upon reasonable request.

## References

[1] D. Beretta , N. Neophytou , J. M. Hodges , et al., “Thermoelectrics: From History, a Window to the Future,” Materials Science and Engineering: R: Reports 138 (2019): 100501.

[2] Z. Bu , X. Zhang , Y. Hu , Z. Chen , S. Lin , W. Li , C. Xiao , and Y. Pei , “A Record Thermoelectric Efficiency in Tellurium‐Free Modules for Low‐Grade Waste Heat Recovery,” Nature Communications 13, no. 1 (2022): 237. 10.1038/s41467-021-27916-y PMC8752736 35017505

[3] J. Mao , H. Zhu , Z. Ding , Z. Liu , G. A. Gamage , G. Chen , and Z. Ren , “High Thermoelectric Cooling Performance of n‐Type Mg3Bi2‐Based Materials,” Science 365, no. 6452 (2019): 495–498. 31320557 10.1126/science.aax7792

[4] L. E. Bell , “Cooling, Heating, Generating Power, and Recovering Waste Heat With Thermoelectric Systems,” Science 321, no. 5895 (2008): 1457–1461. 18787160 10.1126/science.1158899

[5] M. Martin‐Gonzalez , K. Lohani , and N. Neophytou , “Phonon and Electron Transport Engineering for Enhanced Thermoelectric Performance and the Challenges of Device Integration,” Energy Materials 5, no. 9 (2025): 500121.

[6] G. Yang , L. Sang , D. R. Mitchell , et al., “Enhanced Thermoelectric Performance and Mechanical Strength of n‐Type BiTeSe Materials Produced via a Composite Strategy,” Chemical Engineering Journal 428 (2022): 131205.

[7] G. Joshi , X. Yan , H. Wang , W. Liu , G. Chen , and Z. Ren , “Enhancement in Thermoelectric Figure‐of‐Merit of an n‐Type Half‐Heusler Compound by the Nanocomposite Approach,” Advanced Energy Materials 1, no. 4 (2011): 643–647.

[8] Y. Liu , H. Xie , C. Fu , G. J. Snyder , X. Zhao , and T. Zhu , “Demonstration of a Phonon‐Glass Electron‐Crystal Strategy in (Hf, Zr) NiSn Half‐Heusler Thermoelectric Materials by Alloying,” Journal of Materials Chemistry A 3, no. 45 (2015): 22716–22722.

[9] M. Schrade , K. Berland , S. N. Eliassen , et al., “The Role of Grain Boundary Scattering in Reducing the Thermal Conductivity of Polycrystalline XNiSn (X= Hf, Zr, Ti) Half‐Heusler Alloys,” Scientific Reports 7, no. 1 (2017): 1–10. 29062049 10.1038/s41598-017-14013-8 PMC5653807

[10] C. Fu , T. Zhu , Y. Liu , H. Xie , and X. Zhao , “Band Engineering of High Performance p‐Type FeNbSb Based Half‐Heusler Thermoelectric Materials for Figure of Merit ZT> 1,” Energy & Environmental Science 8, no. 1 (2015): 216–220.

[11] J. W. Simonson , D. Wu , W. J. Xie , T. M. Tritt , and S. J. Poon , “Introduction of Resonant States and Enhancement of Thermoelectric Properties in Half‐Heusler Alloys,” Physical Review B 83 (2011): 235211.

[12] N. Neophytou and M. Thesberg , “Modulation Doping and Energy Filtering as Effective Ways to Improve the Thermoelectric Power Factor,” Journal of Computational Electronics 15, no. 1 (2016): 16–26.

[13] M. Thesberg , H. Kosina , and N. Neophytou , “On the Effectiveness of the Thermoelectric Energy Filtering Mechanism in Low‐Dimensional Superlattices and Nano‐Composites,” Journal of Applied Physics 120, no. 23 (2016): 234302‐1–234302‐9.

[14] N. Neophytou , X. Zianni , H. Kosina , S. Frabboni , B. Lorenzi , and D. Narducci , “Simultaneous Increase in Electrical Conductivity and Seebeck Coefficient in Highly Boron‐Doped Nanocrystalline Si,” Nanotechnology 24, no. 20 (2013): 205402. 23598565 10.1088/0957-4484/24/20/205402

[15] M. Martin‐Gonzalez , K. Lohani , and N. Neophytou , “Phonon and Electron Transport Engineering for Enhanced Thermoelectric Performance and the Challenges of Device Integration,” Energy Materials 5, no. 9 (2025): N–A.

[16] A. Masci , E. Dimaggio , N. Neophytou , D. Narducci , and G. Pennelli , “Large Increase of the Thermoelectric Power Factor in Multi‐Barrier Nanodevices,” Nano Energy 132 (2024): 110391.

[17] T. Berry , C. Fu , G. Auffermann , G. H. Fecher , W. Schnelle , F. Serrano‐Sanchez , Y. Yue , H. Liang , and C. Felser , “Enhancing Thermoelectric Performance of TiNiSn Half‐Heusler Compounds via Modulation Doping,” Chemistry of Materials 29, no. 16 (2017): 7042–7048.

[18] A. Kumar , D. K. Kedia , P. Ghosh , and S. Singh , “Band Engineering and Synergistic Modulation Doping for Excellent Thermoelectric Performance in Composites Ti1–x Nb x CoSb–Nb0.8+ δCoSb ,” ACS Applied Energy Materials 6, no. 20 (2023): 10694–10703.

[19] B. Yu , M. Zebarjadi , H. Wang , et al., “Enhancement of Thermoelectric Properties by Modulation‐Doping in Silicon Germanium Alloy Nanocomposites,” Nano Letters 12, no. 4 (2012): 2077–2082. 22435933 10.1021/nl3003045

[20] M. Zebarjadi , G. Joshi , G. Zhu , et al., “Power Factor Enhancement by Modulation Doping in Bulk Nanocomposites,” Nano Letters 11, no. 6 (2011): 2225–2230. 21553899 10.1021/nl201206d

[21] A. Kumar , S. S. Vishak , A. Das , K. Biswas , P. Ghosh , and S. Singh , “Entropy‐Stabilized Half‐Heuslers (TiHf) 1/2 (Fe1–x CoNi1+ x) 1/3Sb With Highly Reduced Lattice Thermal Conductivity,” Chemistry of Materials 37 (2025): 1370–1381.

[22] Y. Liu , H. Xie , C. Fu , G. J. Snyder , X. Zhao , and T. Zhu , “Demonstration of a Phonon‐Glass Electron‐Crystal Strategy in (Hf, Zr) NiSn Half‐Heusler Thermoelectric Materials by Alloying,” Journal of Materials Chemistry A 3, no. 45 (2015): 22716–22722.

[23] S. Chen , K. C. Lukas , W. Liu , C. P. Opeil , G. Chen , and Z. Ren , “Effect of Hf Concentration on Thermoelectric Properties of Nanostructured n‐Type Half‐Heusler Materials HfxZr1–xNiSn0.99Sb0.01,” Advanced Energy Materials 3, no. 9 (2013): 1210–1214.

[24] G. J. Snyder and E. S. Toberer , “Complex Thermoelectric Materials,” Nature Materials 7, no. 2 (2008): 105–114. 18219332 10.1038/nmat2090

[25] A. Pindor , W. Temmerman , and B. Gyorffy , “KKR CPA for Two Atoms per Unit Cell: Application to Pd and PdAg Hydrides,” Journal of Physics F: Metal Physics 13, no. 8 (1983): 1627–1644.

[26] A. Gonis , W. Butler , and G. Stocks , “First‐Principles Calculations of Cluster Densities of States and Short‐Range Order in Ag c Pd 1‐ c Alloys,” Physical Review Letters 50, no. 19 (1983): 1482.

[27] D. D. Johnson , D. Nicholson , F. Pinski , B. Gyorffy , and G. Stocks , “Density‐Functional Theory for Random Alloys: Total Energy within the Coherent‐Potential Approximation,” Physical Review Letters 56, no. 19 (1986): 2088. 10032854 10.1103/PhysRevLett.56.2088

[28] W. Temmerman and Z. Szotek , “Calculating the Electronic Structure of Random Alloys With the KKR‐CPA Method,” Computer Physics Reports 5, no. 4 (1987): 173–220.

[29] L. Bellaiche and D. Vanderbilt , “Virtual Crystal Approximation Revisited: Application to Dielectric and Piezoelectric Properties of Perovskites,” Physical Review B 61, no. 12 (2000): 7877.

[30] Z. Ikonic , R. Kelsall , and P. Harrison , “Virtual‐Crystal Approximation and Alloy Broadening of Intersubband Transitions in p‐Type SiGe Superlattices,” Physical Review B 64, no. 12 (2001): 125308.

[31] A. Baldereschi , K. Maschke , E. Hess , H. Neumann , K.‐R. Schulze , and K. Unger , “Energy Band Structure of AlxGa1‐xAs,” Journal of Physics C: Solid State Physics 10, no. 23 (1977): 4709–4717.

[32] T. Dargam , R. Capaz , and B. Koiller , “Critical Analysis of the Virtual Crystal Approximation,” Brazilian Journal of Physics 27, no. 4 (1997): 299–304.

[33] P. Graziosi , Z. Li , and N. Neophytou , “ELECTRA Code: Full‐Band Electronic Transport Properties of Materials,” Computer Physics Communications 287 (2023): 108670.

[34] C. Martinez‐Blanque , F. G. Ruiz , A. Godoy , E. G. Marin , L. Donetti , and F. Gamiz , “Influence of Alloy Disorder Scattering on the Hole Mobility of SiGe Nanowires,” Journal of Applied Physics 116, no. 24 (2014): 244502‐1–244502‐5.

[35] T. Maeda , H. Hattori , W. H. Chang , Y. Arai , and K. Kinoshita , “Hole Hall Mobility of SiGe Alloys Grown by the Traveling Liquidus‐Zone Method,” Applied Physics Letters 107, no. 15 (2015): 152104‐1–152104‐4.

[36] M. V. Fischetti and S. E. Laux , “Band Structure, Deformation Potentials, and Carrier Mobility in Strained Si, Ge, and SiGe Alloys,” Journal of Applied Physics 80, no. 4 (1996): 2234–2252.

[37] B. Sahni , Y. Zhao , Z. Li , R. Dutt , P. Graziosi , and N. Neophytou , “Thermoelectric Transport and the Role of Different Scattering Processes in the Half‐Heusler NbFeSb,” Materials Horizons 12, no. 23 (2025): 10255–10269. 40859722 10.1039/d5mh00228a

[38] R. Dutt , B. Sahni , Y. Zhao , Y. Go , S. E. A. Akhtar , A. Kumar , S. Kukreti , P. Graziosi , Z. Li , and N. Neophytou , “Carrier Scattering Considerations and Thermoelectric Power Factor of Half‐Heuslers,” Journal of Materials Chemistry A 14 (2026): 10332–10345.

[39] G. Busch and O. Vogt , “Elektrische Leitfähigkeit und Halleffekt von Ge‐Si‐Legierungen,” Helvetica Physica Acta 33 (1960): 437–458.

[40] Z. Li , P. Graziosi , and N. Neophytou , “Deformation Potential Extraction and Computationally Efficient Mobility Calculations in Silicon from First Principles,” Physical Review B 104, no. 19 (2021): 195201.

[41] J. Bardeen and W. Shockley , “Deformation Potentials and Mobilities in Non‐Polar Crystals,” Physical Review 80, no. 1 (1950): 72.

[42] G. Ottaviani , L. Reggiani , C. Canali , F. Nava , and A. Alberigi‐Quaranta , “Hole Drift Velocity in Silicon,” Physical Review B 12, no. 8 (1975): 3318.

[43] K. Takeda , A. Taguchi , and M. Sakata , “Valence‐Band Parameters and Hole Mobility of Ge‐Si Alloys‐Theory,” Journal of Physics C: Solid State Physics 16, no. 12 (1983): 2237–2249.

[44] J. Dewey and M. Osman , “Monte Carlo Study of Hole Transport in Silicon,” Journal of Applied Physics 74, no. 5 (1993): 3219–3223.

[45] M. Fischetti , Z. Ren , P. Solomon , M. Yang , and K. Rim , “Six‐Band k·p Calculation of the Hole Mobility in Silicon Inversion Layers: Dependence on Surface Orientation, Strain, and Silicon Thickness,” Journal of Applied Physics 94, no. 2 (2003): 1079–1095.

[46] “EMAF Code,” GitHub repository, accessed 2026, https://github.com/PatrizioGraziosi/EMAF‐code.

[47] T. Whall , “Two‐Dimensional Hole Gas in SiGe Heterostructures: Electrical Properties and Field Effect Applications,” Journal of Crystal Growth 157, no. 1‐4 (1995): 353–361.

[48] Y. Pei , Z. M. Gibbs , A. Gloskovskii , B. Balke , W. G. Zeier , and G. J. Snyder , “Optimum Carrier Concentration in n‐Type PbTe Thermoelectrics,” Advanced Energy Materials 4, no. 13 (2014): 1400486.

[49] Q. Ren , C. Fu , Q. Qiu , et al., “Establishing the Carrier Scattering Phase Diagram for ZrNiSn‐Based Half‐Heusler Thermoelectric Materials,” Nature Communications 11, no. 1 (2020): 3142. 10.1038/s41467-020-16913-2 PMC7305298 32561856

[50] K. S. Kim , Y.‐M. Kim , H. Mun , et al., “Direct Observation of Inherent Atomic‐Scale Defect Disorders Responsible for High‐Performance Ti1‐xHfxNiSn1‐ySby Half‐Heusler Thermoelectric Alloys,” Advanced Materials 29, no. 36 (2017): 1702091. 10.1002/adma.201702091 28737233

[51] X. Ai , Y. Wu , H. Lyu , et al., “High‐Performance ZrNiSn‐Based Half‐Heusler Thermoelectrics With Hierarchical Architectures Enabled by Reactive Sintering,” Nature Communications 16, no. 1 (2025): 6497. 10.1038/s41467-025-61868-x PMC12259942 40659688

[52] R. A. Downie , S. Barczak , R. Smith , and J.‐W. G. Bos , “Compositions and Thermoelectric Properties of XNiSn (X= Ti, Zr, Hf) Half‐Heusler Alloys,” Journal of Materials Chemistry C 3, no. 40 (2015): 10534–10542.

[53] W. G. Zeier , J. Schmitt , G. Hautier , U. Aydemir , Z. M. Gibbs , C. Felser , and G. J. Snyder , “Engineering Half‐Heusler Thermoelectric Materials Using Zintl Chemistry,” Nature Reviews Materials 1, no. 6 (2016): 1–10.

[54] X. Ai , W. Xue , L. Giebeler , et al., “Interstitial Defect Modulation Promotes Thermoelectric Properties of p‐Type HfNiSn,” Advanced Energy Materials 14, no. 38 (2024): 2401345.

[55] Z. Li , P. Graziosi , and N. Neophytou , “Efficient First‐Principles Electronic Transport Approach to Complex Band Structure Materials: The Case of n‐Type Mg3Sb2,” npj Computational Materials 10, no. 1 (2024): 8.

[56] P. Graziosi and N. Neophytou , “Ultra‐High Thermoelectric Power Factors in Narrow Gap Materials With Asymmetric Bands,” The Journal of Physical Chemistry C 124, no. 34 (2020): 18462–18473.

[57] X.‐Y. Wang , X. Yang , X.‐H. Meng , et al., “Theoretical Insights into the Thermoelectric Transport Performance of the MoP2Ga2S2 Monolayer,” Physical Chemistry Chemical Physics 26, no. 48 (2024): 29913–29921. 39611710 10.1039/d4cp03860f

[58] X. Wang , Y. Shen , X. Yang , X. Meng , Y. Shuai , Q. Ai , and Z. Zhou , “The Regulation of Thermoelectric Transport in Janus1T‐In2OSe Monolayer With Biaxial Tensile Strain,” Physica Scripta 100, no. 8 (2025): 085939.

[59] M. Goyal and M. M. Sinha , “Topological Phase Transition and Lattice Dynamical Properties of Half‐Heusler XPtBi (X= Gd, Nd),” Physica Status Solidi (b) 260, no. 4 (2023): 2200463.

[60] H. B. Kang , B. Poudel , W. Li , et al., “Decoupled Phononic‐Electronic Transport in Multi‐Phase n‐Type Half‐Heusler Nanocomposites Enabling Efficient High Temperature Power Generation,” Materials Today 36 (2020): 63–72.

[61] Y. Zhang , G. Peng , S. Li , et al., “Phase Interface Engineering Enables State‐of‐the‐Art Half‐Heusler Thermoelectrics,” Nature Communications 15, no. 1 (2024): 5978. 10.1038/s41467-024-50371-4 PMC11252142 39013905

[62] R. Yan , C. Shen , M. Widenmeyer , et al., “The Role of Interstitial Cu on Thermoelectric Properties of ZrNiSn Half‐Heusler Compounds,” Materials Today Physics 33 (2023): 101049.

[63] Q. Lou , Z. Gao , S. Han , F. Liu , C. Fu , and T. Zhu , “High Defect Tolerance in Heavy‐Band Thermoelectrics,” Advanced Energy Materials 14, no. 41 (2024): 2402399.

[64] Rahul , B. Sharma , S. Pal , and N. Verma , “Synthesis of Phase‐Pure n‐Type TiNiSn Half‐Heusler Thermoelectric Material Using Reactive Hot Pressing,” ACS Applied Energy Materials 8, no. 14 (2025): 10037–10049.

[65] H. Zhu , W. Li , A. Nozariasbmarz , N. Liu , Y. Zhang , S. Priya , and B. Poudel , “Half‐Heusler Alloys as Emerging High Power Density Thermoelectric Cooling Materials,” Nature Communications 14, no. 1 (2023): 3300. 10.1038/s41467-023-38446-0 PMC10244423 37280195

[66] S. Ye , S. Zhi , X. Ma , et al., “Superior Electron Transport in the Single‐Crystalline TiCoSb‐Based Half‐Heuslers,” Nature Communications 16, no. 1 (2025): 1812. 10.1038/s41467-025-56961-0 PMC11850857 39994177

[67] A. Togo , L. Chaput , and I. Tanaka , “Distributions of Phonon Lifetimes in Brillouin Zones,” Physical Review B 91 (2015): 094306.

[68] A. Togo , L. Chaput , T. Tadano , and I. Tanaka , “Implementation Strategies in Phonopy and Phono3py,” Journal of Physics: Condensed Matter 35, no. 35 (2023): 353001. 10.1088/1361-648X/acd831 37220761

[69] S. N. Eliassen , A. Katre , G. K. Madsen , C. Persson , O. M. Løvvik , and K. Berland , “Lattice Thermal Conductivity of ${\mathrm{Ti}}_{x}{\mathrm{Zr}}_{y}{\mathrm{Hf}}_{1-xy}\mathrm{NiSn}$ Half‐Heusler Alloys Calculated from First Principles: Key Role of Nature of Phonon Modes,” arXiv (2016): arXiv:1611.01757.

[70] J. Carrete , W. Li , N. Mingo , S. Wang , and S. Curtarolo , “Finding Unprecedentedly Low‐Thermal‐Conductivity Half‐Heusler Semiconductors via High‐Throughput Materials Modeling,” Physical Review X 4, no. 1 (2014): 011019.

[71] P. Klemens , “Thermal Resistance Due to Point Defects at High Temperatures,” Physical Review 119, no. 2 (1960): 507.

[72] R. Gurunathan , R. Hanus , and G. J. Snyder , “Alloy Scattering of Phonons,” Materials Horizons 7, no. 6 (2020): 1452–1456.

[73] D. Rabin , D. Fuks , and Y. Gelbstein , “Alloying Effect on the Lattice Thermal Conductivity of MNiSn Half‐Heusler Alloys,” Physical Chemistry Chemical Physics 25, no. 1 (2022): 520–528. 36477717 10.1039/d2cp04653a

[74] J. Yang , G. Meisner , and L. Chen , “Strain Field Fluctuation Effects on Lattice Thermal Conductivity of ZrNiSn‐Based Thermoelectric Compounds,” Applied Physics Letters 85, no. 7 (2004): 1140–1142.

[75] M. Zhou , L. Chen , W. Zhang , and C. Feng , “Disorder Scattering Effect on the High‐Temperature Lattice Thermal Conductivity of TiCoSb‐Based Half‐Heusler Compounds,” Journal of Applied Physics 98, no. 1 (2005): 013708‐1–013708‐5.

[76] H. Geng and H. Zhang , “Effects of Phase Separation on the Thermoelectric Properties of (Ti, Zr, Hf) NiSn Half‐Heusler Alloys,” Journal of Applied Physics 116, no. 3 (2014): 033708‐1–033708‐7.

[77] K. Yadav , S. Singh , O. Muthuswamy , T. Takeuchi , and K. Mukherjee , “Unravelling the Phonon Scattering Mechanism in Half‐Heusler Alloys ZrCo1‐x Ir x Sb (x= 0, 0.1 and 0.25),” Journal of Physics: Condensed Matter 34, no. 3 (2022): 035702. 10.1088/1361-648X/ac30b5 34663764

[78] H. Zhu , J. Mao , Y. Li , et al., “Discovery of TaFeSb‐Based Half‐Heuslers With High Thermoelectric Performance,” Nature Communications 10, no. 1 (2019): 270. 10.1038/s41467-018-08223-5 PMC6336844 30655512

[79] R. He , D. Kraemer , J. Mao , et al., “Achieving High Power Factor and Output Power Density in p‐Type Half‐Heuslers Nb1‐xTixFeSb,” Proceedings of the National Academy of Sciences 113, no. 48 (2016): 13576–13581. 10.1073/pnas.1617663113 PMC5137697 27856743

[80] A. Kumar , S. Vishak , P. Ghosh , and S. Singh , “Defect‐Assisted Ultrahigh ZT of TaFeSb Based Half‐Heuslers,” Small 22, no. 37 (2026): e73765. 42125785 10.1002/smll.73765

[81] A. van de Walle , P. Tiwary , M. de Jong , et al., “Efficient Stochastic Generation of Special Quasirandom Structures,” Calphad 42 (2013): 13–18.

[82] G. A. Naydenov , P. J. Hasnip , V. Lazarov , and M. Probert , “Huge Power Factor in p‐Type Half‐Heusler Alloys NbFeSb and TaFeSb,” Journal of Physics: Materials 2, no. 3 (2019): 035002.

[83] S. E. A. Akhtar and N. Neophytou , “Conditions for Thermoelectric Power Factor Improvements upon Band Alignment in Complex Bandstructure Materials,” ACS Applied Energy Materials 8, no. 3 (2025): 1609–1619. 39949821 10.1021/acsaem.4c02747 PMC11815832

[84] C. Kumarasinghe and N. Neophytou , “Band Alignment and Scattering Considerations for Enhancing the Thermoelectric Power Factor of Complex Materials: The Case of Co‐Based Half‐Heusler Alloys,” Physical Review B 99, no. 19 (2019): 195202.

[85] J. Yu , C. Fu , Y. Liu , K. Xia , U. Aydemir , T. C. Chasapis , G. J. Snyder , X. Zhao , and T. Zhu , “Unique Role of Refractory Ta Alloying in Enhancing the Figure of Merit of NbFeSb Thermoelectric Materials,” Advanced Energy Materials 8, no. 1 (2018): 1701313.

[86] C. Fu , T. Zhu , Y. Pei , et al., “High Band Degeneracy Contributes to High Thermoelectric Performance in p‐Type Half‐Heusler Compounds,” Advanced Energy Materials 4, no. 18 (2014): 1400600.

[87] W. Ren , H. Zhu , Q. Zhu , et al., “Ultrahigh Power Factor in Thermoelectric System Nb0.95M0.05FeSb (M= Hf, Zr, and Ti),” Advanced Science 5, no. 7 (2018): 1800278. 30027058 10.1002/advs.201800278 PMC6051200

[88] P. Giannozzi , S. Baroni , N. Bonini , et al., “Quantum ESPRESSO: A Modular and Open‐Source Software Project for Quantum Simulations of Materials,” Journal of Physics: Condensed Matter 21, no. 39 (2009): 395502. 21832390 10.1088/0953-8984/21/39/395502

[89] P. Giannozzi , O. Andreussi , T. Brumme , et al., “Advanced Capabilities for Materials Modelling With Quantum ESPRESSO,” Journal of Physics: Condensed Matter 29, no. 46 (2017): 465901. 29064822 10.1088/1361-648X/aa8f79

[90] J. P. Perdew , K. Burke , and M. Ernzerhof , “Generalized Gradient Approximation Made Simple,” Physical Review Letters 77 (1996): 3865–3868. 10062328 10.1103/PhysRevLett.77.3865

[91] H. Lee , S. Poncé , K. Bushick , et al., “Electron–Phonon Physics from First Principles Using the EPW Code,” npj Computational Materials 9, no. 1 (2023): 156.

[92] H. J. Monkhorst and J. D. Pack , “Special Points for Brillouin‐Zone Integrations,” Physical Review B 13, no. 12 (1976): 5188.

[93] “thermo_pw,” accessed 2026, https://github.com/dalcorso/thermo_pw.

[94] M. Glicksman , “Mobility of Electrons in Germanium‐Silicon Alloys,” Physical Review 111, no. 1 (1958): 125.

