# Supplementary material for: Ab Initio Thermoelectric Transport Calculations of Alloyed Half‐Heuslers
Source: Adv Sci (Weinh). 2026 Sep 9:e77630. Online ahead of print. doi: 10.1002/advs.77630 (PMC13558921; doi:10.1002/advs.77630)
Supplement: Supplementary file 1 [file ADVS-9999-e77630-s001.pdf]

SI: *Ab initio* thermoelectric transport calculations of alloyed half-Heuslers

Ankit Kumar Bhawna Sahni Neophytos Neophytou**

This supporting information file contains: i) tables for the input parameters to the simulations, ii) electronic structures and effective masses to understand the effect of alloying, iii) quantities that affect the transport properties such as DOS, band velocity, band gap, transport distribution functions (TDF), relaxation times of electrons, holes and phonon.
Tables (S1, S2, S3) list the calculated parameters required as inputs in the transport calculations. *Ab initio* calculation is performed for all the pristine materials listed in the tables except deformation potential (DP) calculations in some cases, which are taken from prior studies. The DP of Si and Ge is chosen to match the experimental mobility values. The DPs for ZrNiSn, HfNiSn, and NbFeSb are from Reference [1, 2]. A brief discussion of the DP calculation method is described in Section 1. Linearly interpolated values are used for the intermediate alloy compositions. The validity of the approximation is discussed in Section 2. Table S4 shows the band effective masses for the eight bands of pristine TaFeSb along with those of Ti, Zr, and Hf doped materials.
Figure S1 shows a comparison of band and transport quantities (DOS, velocity, TDF, and PF) between the pristine (2-atom) and cubic (8-atom) structures. Figure S2 shows the electronic structures and variation of effective masses of Si-Ge alloys as a function of x.
Figure S3 and S4 shows a schematic band folding process and error in PF depending on the choice of supercell.
Figure S7, S8, and S9 shows the band structures, band gap, and phonon dispersion with alloying in XNiSn type HH.
Figure S10, and S11 shows the liner variation of physical parameters and error in PF when there is deviation from linearity. A comparison of alloy scattering with IIS and POP is shown for HH alloys in Figure S12. The PFs as a function of Fermi energy at 300 K for all these compositions are shown in Figures S13 and S14. Effect of bipolarity is shown in Figure S15 and comparison transport coefficient is done with experimental data is shown in Figure S16
Figure s17 shows the band velocities in n- and p-type $Ti_{1-x}Zr_xNiSn$ materials. Change in band gap with alloying is shown in Figure S18.
Figure S19 shows the lattice thermal conductivity (κ_L) as a function of temperature for the noted materials. Alloyed compositions suffer drastic reduction in κ_L. The phonon lifetimes are shown in Figure S20 to justify such reduction. The lifetime of the alloyed compositions are around half compared to those of the pristine compositions for both acoustic and optical phonon. A full calculation and comparison with reported κ_L is done for all the composition for 300 K, 600 K, and 900 K in Figure S21. The electronic part of the thermal conductivity is shown in Figure S22. The values here are taken at the Fermi level of maximum PF.
Figure S23 shows the comparison of transport coefficient of pristine TaFeSb with experimental reported values For Ti doped TaFeSb. Figure S24 shows the atomic projected band structure of TaFeSb. The conduction band has major contribution from Ta, whereas valence band has major contributions from Fe. The band splitting correlation with alloying is shown in Figure S25. The phonon dispersion for a few composition is shown in Figure S26 showing the dynamical stability of alloyed composition with κ_L variation in Figure S28
Figure S27 shows the comparison of PF, Seebeck coefficient, conductivity, and TDF between pristine n-type TaFeSb and n-type $Ta_{0.5}Nb_{0.5}FeSb$. Figure 29 shows the validity of p-type TaFeSb and associated error when 2×2×2 supercell consideration. Figure 30 shows the comparison of DOS, band velocity and relaxation times for TaFeSb, $Ta_{0.875}Ti_{0.125}FeSb$, $Ta_{0.875}Zr_{0.125}FeSb$, and $Ta_{0.875}Hf_{0.125}FeSb$. Comparisons of the Seebeck coefficient are shown in Figure S32. Similar comparison of Seebeck, conductivity and PF is shown in Figure S31, using parabolic band model.

1 Deformation potential extraction method

We describe phonon scattering within the deformation potential theory [3]. We extract deformation potentials through electron–phonon coupling matrix elements, from which scattering rates can be computed using Fermi's Golden rule. The scattering matrix elements, describing transitions from an initial state $|\psi_{n\mathbf{k}}\rangle$ to a final state $|\psi_{m\mathbf{k+q}}\rangle$ due to a perturbation $\delta_{\mathbf{q}\nu}V$, are defined as [4, 5, 6]:

$$M_{mn\nu}(\mathbf{k},\mathbf{q}) = \langle\psi_{n\mathbf{k}}|\delta_{\mathbf{q}\nu}V|\psi_{m\mathbf{k+q}}\rangle \tag{S1}$$

The acoustic deformation potential (ADP) is obtained by taking the slope of the matrix elements with respect to the phonon wave vectors as [7]:

$$D_{\mathrm{ADP}} = \frac{M_{mn\nu}(\mathbf{k},\mathbf{q})}{|\mathbf{q}|} \tag{S2}$$

The optical deformation potential (ODP) is the value of the matrix element itself:

$$D_{\mathrm{ODP}} = M_{mn\nu}(\mathbf{k},\mathbf{q}) \tag{S3}$$

In polar materials, long-wavelength longitudinal optical (LO) phonons generate a macroscopic electric field, leading to the Fröhlich interaction [8]. The long-range component of the electron–phonon matrix element is given by [9]:

$$M^{\mathrm{L}}_{mn\nu}(\mathbf{k},\mathbf{q}) = i\frac{m_0^{1/2}e^2}{\Omega\varepsilon_0}\sum_k M_k^{-1/2}\sum_{\mathbf{G}\neq-\mathbf{q}}\frac{(\mathbf{q}+\mathbf{G})\cdot\mathbf{Z}^*_k\cdot\mathbf{e}_{k\nu}(\mathbf{q})}{(\mathbf{q}+\mathbf{G})\cdot k_\infty\cdot(\mathbf{q}+\mathbf{G})}\langle u_{mk+q}|e^{i(\mathbf{q}+\mathbf{G})\cdot\mathbf{r}}|u_{nq}\rangle \tag{S4}$$

Here, Ω is the unit cell volume, m_0 the total atomic mass, M_k the atomic mass, ε_0 the vacuum permittivity, $\mathbf{G}$ the reciprocal lattice vector, $\mathbf{e}_{k\nu}(\mathbf{q})$ the phonon eigenmode, k_∞ the high-frequency dielectric constant, and $\mathbf{Z}^*_k$ the Born effective charge tensor. In polar materials, phonon modes include both short- and long-range (POP) contributions [10]; the latter is subtracted from the total matrix elements to remove the $|\mathbf{q}| \rightarrow 0$ divergence, yielding the short-range component used to compute D_{ADP} and D_{ODP}. The overall D_{ADP} value is calculated after averaging the longitudinal and transverse acoustic deformation potentials as:

$$D_{\mathrm{ADP}} = v_s\sqrt{\frac{D^2_{\mathrm{ADP,LA}}}{v_l^2}+\frac{D^2_{\mathrm{ADP,TA}}}{v_t^2}} \tag{S5}$$

where v_s, v_l and v_t are the average, longitudinal, and transverse sound velocities, respectively.
The D_{ODP} value is calculated by taking the average of the short-range components of all optical modes for small q-vector values near the Γ point as:

$$D_{\mathrm{ODP}} = \sqrt{\omega_{\mathrm{ODP}}\sum_\nu\frac{|M_{mn\nu}(\mathbf{k},\mathbf{q})-M^{\mathrm{L}}_{mn\nu}(\mathbf{k},\mathbf{q})|^2}{\omega_\nu}} \tag{S6}$$

where, ω_{ODP} is the average value of optical phonon energy. Both of these deformation potentials are calculated along different crystallographic directions to take anisotropy into account, and then averaged to calculate the overall deformation potentials. More detailed description of calculation method can be found in Ref. [2].

2 Deformation potential for supercell:

Computationally prohibitive problem el-ph interactions in supercells: As explained in the previous section, we have used the EPW code to extract the deformation potential of all the pristine compositions, which itself is highly computationally expensive. These calculation typically require the computation and further post-processing of millions of matrix elements and need a very fine sampling of the

electron and phonon momentum spaces, making scaling quite challenging and impractical for larger systems [11, 12, 13]. The computational cost to extract deformation potentials largely depends on the size of the unit cell. For pristine HHs (with a 3-atom unit cell) using our DFPT+Wanner method, is around 12000 CPU hours with an additional 6000 CPU hours for the full transport calculation with ElecTra. (In contrast, the full transport calculation with EPW requires more than 70000 CPU hours [2]). For a 5-atoms unit cell materials such as Mg_3Sb_2, the computational time in one of our previous studies exceeded a million CPU hours [14]. Therefore, performing such calculations for supercells (12-atom or 24-atom systems) is completely prohibitive, and this is why it has not been performed so far. With more atoms in the system, even the Wannier function convergence require larger k-grid and larger q-grid within DFPT calculations, which will increase the computational cost exponentially. Thus, electron-phonon interactions for alloys are essentially impossible at this point.

Linear approximation and associated error: Linear interpolation of parameters with alloying is standard practice and has been very successful in the past for SiGe and AlAs-GaAs alloyed compositions [15, 16]. The VCA method used in such alloys itself uses linear interpolation to create the virtual crystal [17]. Along with VCA, the variation in scattering parameters is also considered to vary linearly with alloy composition to obtain mobilities close to experiment.
To further justify our assumption for HH, we have calculated the variation in parameters like dielectric constant and sound velocity along with D_{ADP} using the simple band shift method to estimate global ADP values (as implemented in AMSET code by straining the lattice and extracting the band shift [18]). We use the alloyed compositions of $Ti_{1-x}Zr_xNiSn$ and $Ta_{1-x}Nb_xFeSb$ as example to demonstrate this linear trend for D_{ADP} with alloying, using the band shift method. For this, we vary the lattice volume (V) of the composition and record the shift in the energy of band edge (E_{edge}). For the deformation potential, we then use:

$$D_{\text{ADP}} = \frac{d(E_{edge} - E_{ref})}{d(lnV)} \tag{S7}$$

where E_{ref} is the reference energy before strain. As the DFT calculation for band structure assumes periodic boundary conditions, the absolute position of the energy levels in a periodic crystal include an unknown constant shift. Therefore, a reference level (E_{ref}) is required to obtain the absolute value of the change in the band edge with respect to volume change. There are many proposed methods for the E_{ref} consideration. The objective is to have a energy reference which is unaffected by volume change, such as a deep core level, the dielectric midgap energy level, the first valence electron energy band etc. [19, 20, 21], etc.. Each of the assumed methods produce different D_{ADP} values as shown in Figure S1 of Ref [21]. Our objective here is to see the variation of D_{ADP} values with alloying, irrespective of the absolute values. We considered the simplest among all the proposed methods, i.e. choosing the first valence electron energy band as E_{ref}. This method is widely used for high throughput calculation of ADP values for HHs [21]. Figure S10a shows the D_{ADP} values for $Ti_{1-x}Zr_xNiSn$ and $Ta_{1-x}Nb_xFeSb$ as a function of x. Even though the values are not accurate in the absolute sense, they indeed follow an overall linear trend. Similar variation for the D_{ADP} is reported for $Hf_{1-x}Zr_xCoBi$, where the maximum deviation from linearity is only 2.5 % [22]. Beyond the deformation potentials, we have calculated other relevant parameters such as the sound velocity and dielectric constants, which strictly follow linear variation with alloying compositions, as shown in Figure S10b, c, d. Therefore, overall, it is reasonable to consider that the linear interpolation would hold for all parameters for the alloy compositions considered.
Due to the reduced strength of the ADP+ODP mechanisms compared to IIS+POP, which do not require matrix elements, even large errors in the deformation potential values will not affect the PF. For example, even using a combined ADP + ODP deformation potential values that deviate form linearity by 20 % for $Ti_{0.5}Zr_{0.5}NiSn$, the error in the maximum PF is less than 5 % (see Figure S11). Overall, we have also concluded in this work that the change in deformation potential with alloying, is not as important as the change in electronic structure such as band splittings or band curvature affects, which affect the PF significantly. Inaccuracies in the linear variation assumptions for the deformation potentials will introduce an error of less than 5%.

3 Klemens model for lattice thermal conductivity

Lattice thermal conductivity of pristine TiNiSn, ZrNiSn, and HfNiSn is calculated using phono3py [23, 24]. However, the phonon transport calculations are quite expensive for supercells using phono3py, as a very large number of displaced atomic configurations (≈28000, depending on symmetry) for the supercell (96 atoms) are needed for the 12-atom system.
In order to estimate the κ_L for the alloyed composition, we have used Klemens model [25, 26]. There are various reports on XNiSn and other HH alloys in which this model has been used successfully [27, 28, 29, 30, 31, 32]. The lattice thermal conductivity for alloys (κ_L) are calculated using the lattice thermal conductivity of pure compounds (κ_0) and scattering parameter (Γ) as:

$$\frac{\kappa_L}{\kappa_0} = \frac{tan^{-1}u}{u}, \quad u^2 = \frac{(6\pi^5 V^2)^{1/3}}{2k_B \nu_s}\kappa_0 \Gamma \tag{S8}$$

where V is the average volume per atom and ν_s is the average sound velocity. The disorder parameter Γ is composed of two parts; disorder because of mass difference (Γ_m) and size difference ($\epsilon\Gamma_s$), where ϵ is a phenomenological parameter that can be used for fitting experimental results. For an XNiSn alloy, where there is a substitution of different elements only in the X sub-lattice of a compound with 3 lattices, the mass (Γ_m) and strain (Γ_s) variance parameters are defined as:

$$\Gamma_m = \frac{\frac{1}{3}\sum(x_i(M_i - M_{avg})^2)}{(\frac{1}{3}(M_{avg} + M_{Ni} + M_{Ni}))^2} \tag{S9}$$

$$\Gamma_s = \frac{\frac{1}{3}\sum(x_i(R_i - R_{avg})^2)}{(\frac{1}{3}(R_{avg} + R_{Ni} + R_{Ni}))^2} \tag{S10}$$

where x is the site fraction of an element in the X sub-lattice of XNiSn, M_i/R_i are the mass/atomic radius of an element in the X sub-lattice and M_{avg}/R_{avg} are the average mass/radius of the elements in the X sub-lattice.
In order to have better estimation of disorder parameter (Γ), we have calculated the κ_L for intermediate composition, $Ti_{0.5}Zr_{0.5}NiSn$, $Zr_{0.5}Hf_{0.5}NiSn$, and $Hf_{0.5}Ti_{0.5}NiSn$, using Phono3Py. With intermediate data points, we tuned the ϵ such that that κ_L calculated using the model fits the calculated values of Phono3Py. The fitted curves are shown in Figure S21. We also compared our results with experimental reported κ_L. In all the alloyed compositions (x = 0.25, 0.5, and 0.75), κ_L is reduced and remains in the same range of value as estimated by the model (see Figure S21). Similar calculation is done for Nb alloyed TaFeSb where only mass disorder is used as Ta and Nb has similar size (shown in Figure S28).

Table S1: Input parameters to the BTE for describing scattering in the calculation of the transport coefficients for Si and Ge. Equilibrium values of lattice constant (a), values for acoustic deformation potentials (D_{ADP}), and optical deformation potentials (D_{ODP}) are presented. $\hbar\omega_{\mathrm{ODP}}$ is the highest energy of the transverse optical phonon mode (TO) at the Γ point. k_∞ is the high frequency dielectric constant. A linear interpolation of parameters is considered as a function of x in $Si_{1-x}Ge_x$.

Composition	Si	Ge
a [Å]	5.47	5.77
density [g/cm^3]	2.33	5.02
k_∞	15.19	25.85
ν_s [m/s]	5812	5862
D_{ADP}(h) [eV]	5.0	3.6
D_{ODP}(h) [eV/Å]	7.63	7.45
$\hbar\omega_{\mathrm{ODP}}$ [meV]	63.0	41.0

Table S2: Scattering parameters used in the calculation of the transport coefficients for TiNiSn, ZrNiSn, HfNiSn. Equilibrium values of lattice constant (a), values for acoustic deformation potentials (D_{ADP}), and optical deformation potentials (D_{ODP}) are presented. $\hbar\omega_{\mathrm{ODP}}$ and $\hbar\omega_{\mathrm{POP}}$ is the highest energy of the transverse optical (TO) and longitudinal optical (LO) phonon modes at the Γ point. k_s and k_∞ are the static and high frequency dielectric constants. A linear interpolation of parameters is considered for alloyed composition between any two pristine TiNiSn, ZrNiSn, and HfNiSn materials.

Composition	TiNiSn	ZrNiSn	HfNiSn
a [Å]	5.94	6.11	6.07
density [g/cm^3]	7.15	7.81	10.56
k_∞	23.95	21.49	20.36
k_s	30.58	26.55	25.29
ν_s [m/s]	3469	3489	3096
D_{ADP}(e) [eV]	2.55	2.67	1.87
D_{ODP}(e) [eV/Å]	0.92	2.12	2.09
D_{ADP}(h) [eV]	2.52	2.70	3.32
D_{ODP}(h) [eV/Å]	1.04	1.34	1.40
$\hbar\omega_{\mathrm{POP}}$ [meV]	31.3	28.2	26.4
$\hbar\omega_{\mathrm{ODP}}$ [meV]	29.3	26.6	25.0

Table S3: Scattering parameters used in the calculation of the transport coefficients for $Ta_{1-x}Nb_xFeSb$. Equilibrium values of lattice constant (a), values for acoustic deformation potentials (D_{ADP}), and optical deformation potentials (D_{ODP}) are presented. $\hbar\omega_{\mathrm{ODP}}$ and $\hbar\omega_{\mathrm{POP}}$ is the highest energy of the transverse optical (TO) and longitudinal optical (LO) phonon modes at the Γ point. k_s and k_∞ are the static and high frequency dielectric constants.

x =	0.0	0.125	0.25	0.375	0.50	0.625	0.75	0.875	1.0
a [Å]	5.95	5.95	5.95	5.95	5.95	5.95	5.95	5.95	5.95
density [g/cm^3]	11.31	10.95	10.59	10.25	9.89	9.55	9.19	8.84	8.50
k_∞	21.26	21.64	22.01	22.41	22.79	23.21	23.60	24.03	24.43
k_s	36.74	37.57	38.43	39.42	40.10	41.28	42.19	43.22	44.13
$\nu_s$$^@$ [m/s]	3183	3222	3261	3301	3340	3379	3418	3458	3497
D_{ADP}(e) [eV]$^@$	3.39	3.55	3.72	3.88	4.04	4.21	4.37	4.53	4.70
D_{ODP}(e) [eV/Å]$^@$	2.71	2.78	2.86	2.93	3.0	3.08	3.15	3.22	3.30
D_{ADP}(h) [eV]$^@$	2.98	2.92	2.86	2.80	2.74	2.68	2.62	2.56	2.50
D_{ODP}(h) [eV/Å]$^@$	2.78	2.78	2.79	2.79	2.79	2.79	2.80	2.80	2.80
$\hbar\omega_{\mathrm{POP}}$ [meV]$^@$	32.0	32.0	32.0	32.0	32.0	32.0	32.0	32.0	32.0
$\hbar\omega_{\mathrm{ODP}}$ [meV]$^@$	27.50	27.68	27.87	28.06	28.25	28.44	28.62	28.81	29.00

$^@$ a liner interpolation of values are considered going from x = 0.0 to 1.0.

Table S4: Band effective mass (in units of m_e) of the eight valence bands of TaFesb, $Ta_{0.875}Ti_{0.125}FeSb$, $Ta_{0.875}Zr_{0.125}FeSb$, and $Ta_{0.875}Ti_{0.125}FeSb$.

Band No.	TaFeSb	$Ta_{0.875}Ti_{0.125}FeSb$	$Ta_{0.875}Zr_{0.125}FeSb$	$Ta_{0.875}Hf_{0.125}FeSb$
B1	1.42	1.52	1.55	1.6
B2	1.42	1.39	1.41	1.47
B3	1.42	1.01	1.0	1.04
B4	1.42	0.95	1.1	1.09
B5	0.89	0.91	0.91	0.86
B6	0.89	0.92	1.38	1.32
B7	0.89	0.84	1.0	1.0
B8	0.89	0.84	0.84	0.86

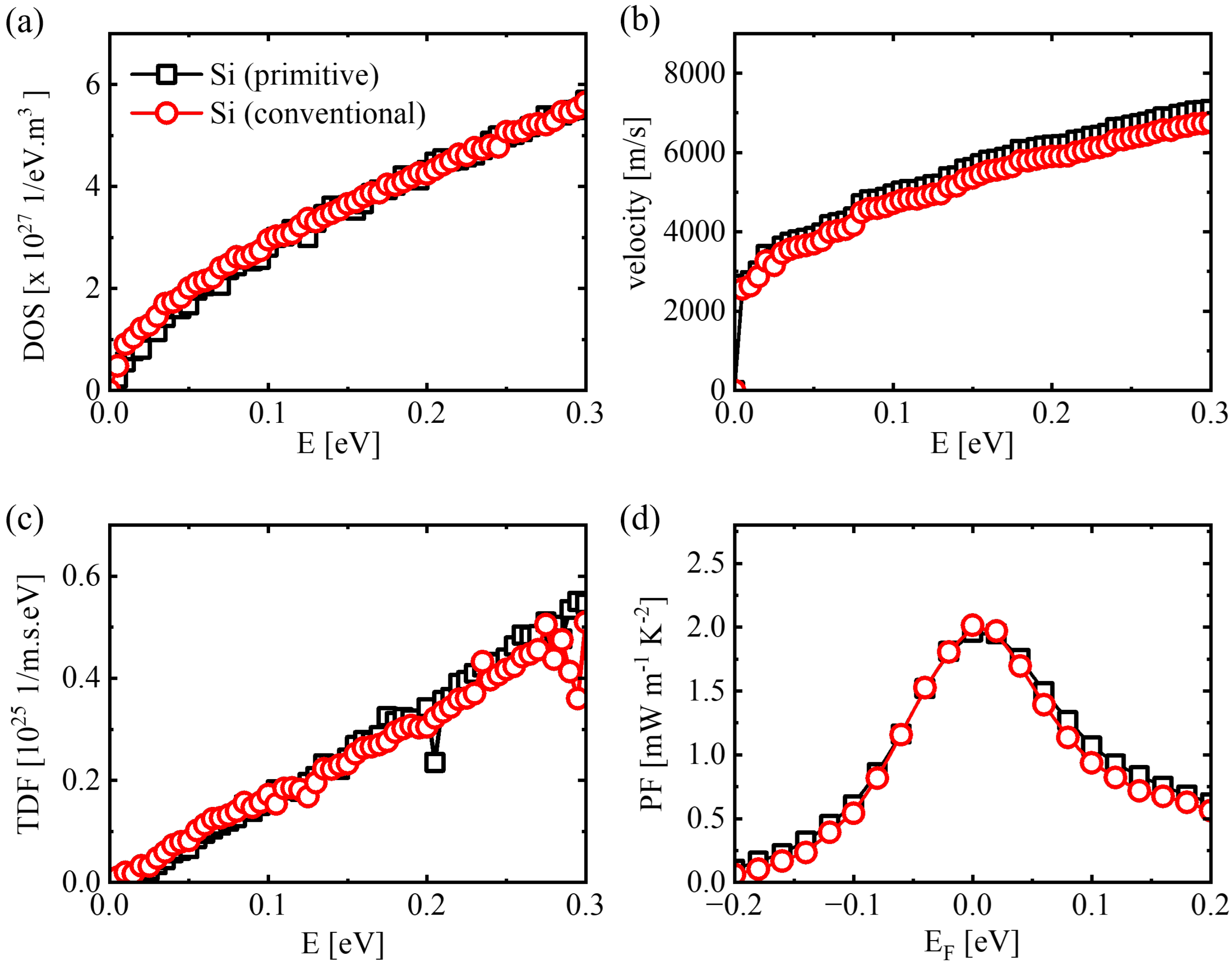

Figure S1: (a-c) Comparison of the density of states, band velocity and transport distribution functions (TDF) for p-type Si in the primitive and cubic structures, as noted in the legend. (d) Comparison of PFs versus Fermi energy for the two cases.

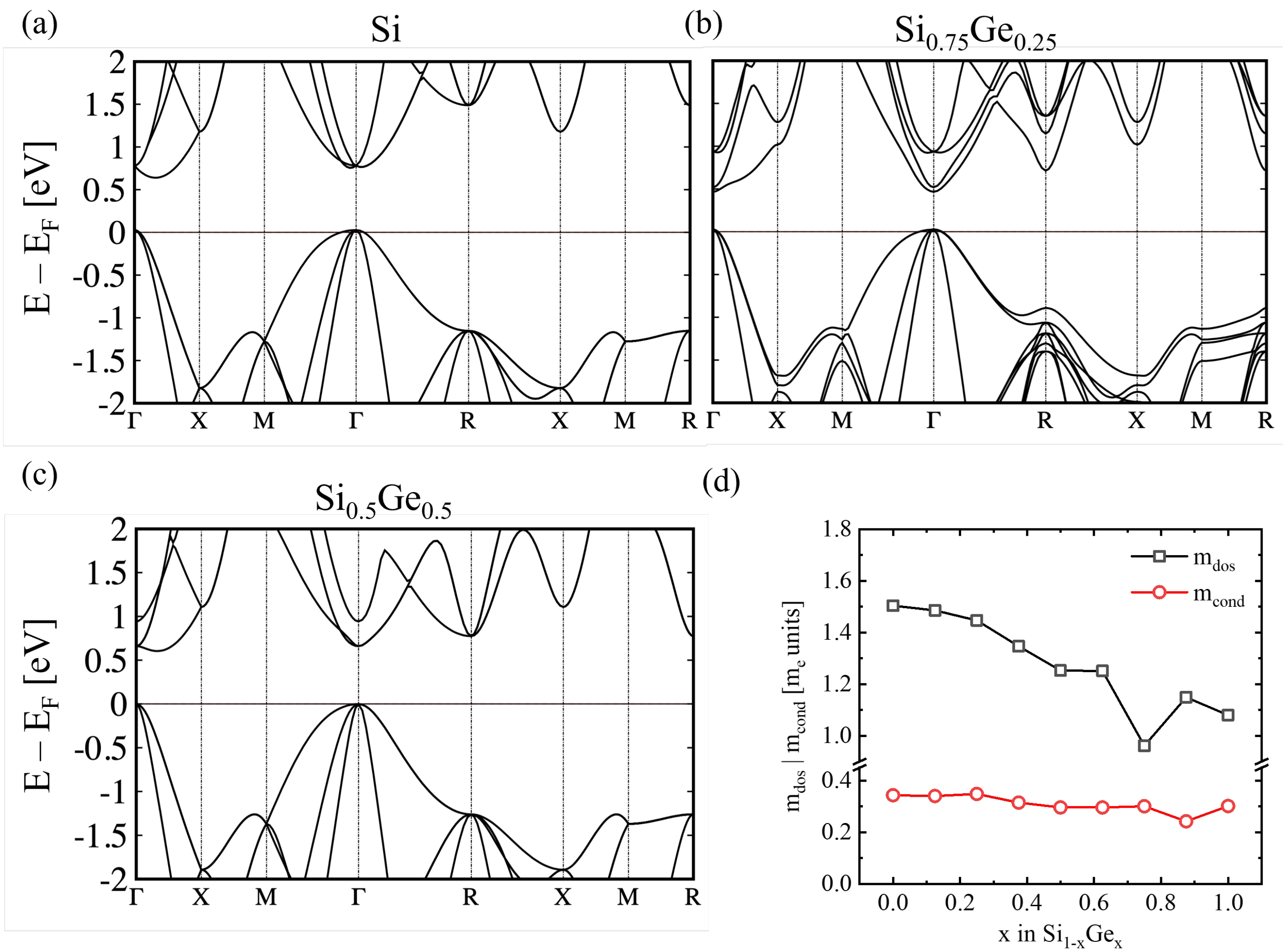

Figure S2: (a) Band structure of pristine Si. (b,c) Band structures of $Si_{0.75}Ge_{0.25}$, and $Si_{0.5}Ge_{0.5}$ in cubic symmetry. (d) Density of states and conductivity effective masses as a function of Ge alloying.

mobility comparison of Si-Ge at high carrier density:
The comparison of mobility revealed that alloy scattering reduces the mobility significantly at low carrier density, but it has a small effect at high carrier densities because of IIS which is much stronger. We have compared the mobility for a few compositions as a function of carrier concentration. Figure S3 shows the calculated mobility at high carrier densities along with experimental reported values for Si [33, 34, 35, 36], $Si_{0.90}Ge_{0.10}$ [37], $Si_{0.80}Ge_{0.20}$ [38], and $Si_{0.70}Ge_{0.30}$ [39, 40]. In all cases, the high carrier density mobility is lower than the calculated ones, which is typical. The reasons could be the additional disorder created by ionised impurity doping to achieve such high carrier densities (Boron or Aluminum doping), or the polycrystallinity of the materials, which introduces additional grain boundary scattering, plus additional defects, dislocations, etc., that are often present. In the calculation we are only considering the effect of alloying in pristine Si-Ge alloy. Comparing the influence of composition, however across these measured data, we observe that they all reside around the non-alloyed Si material (black square symbols), indicating that the effect of alloy scattering of up to 30% Ge in this case, is not strong enough compared to IIS to affect mobility.

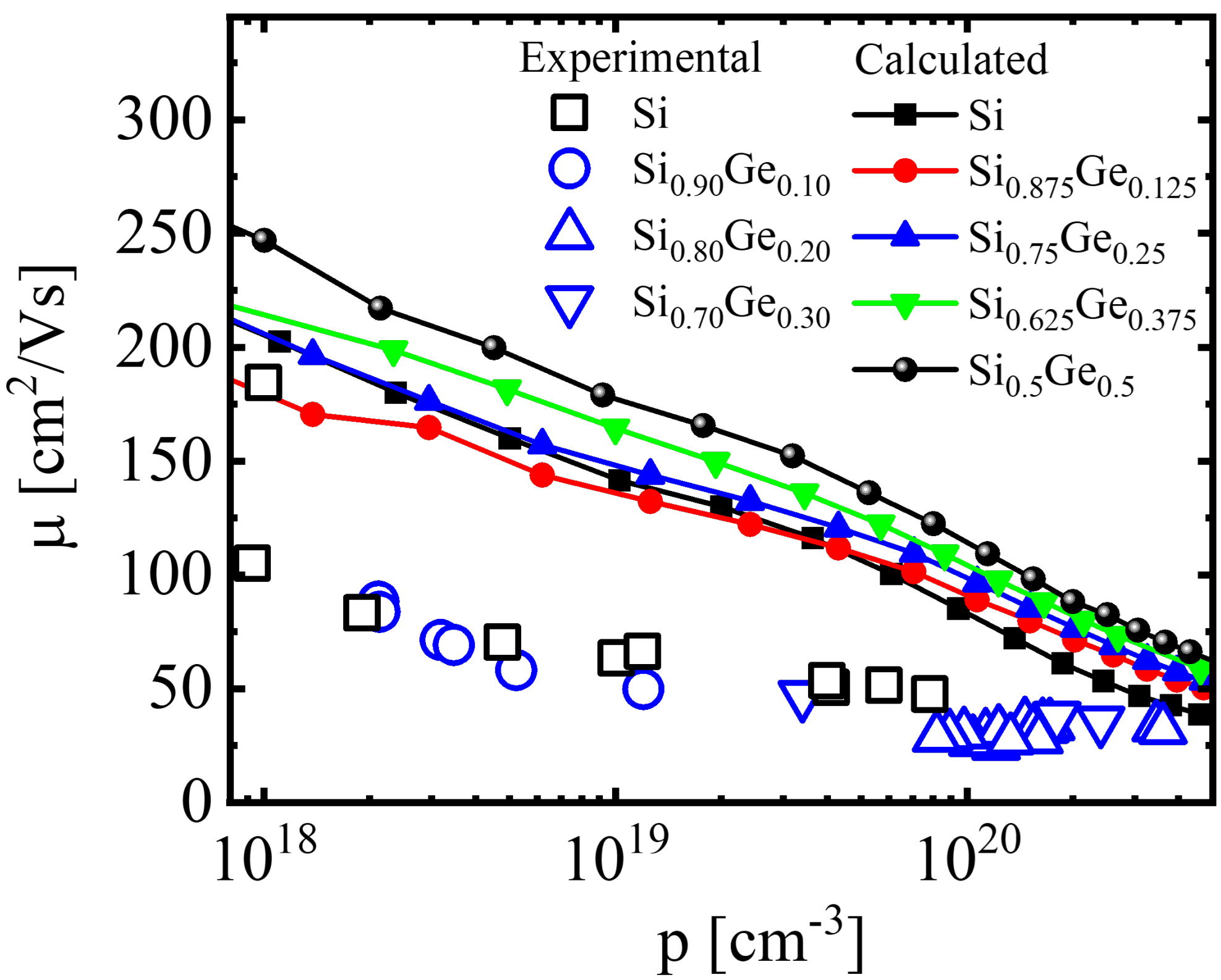

Figure S3: Experimental and computational hole mobility in the high carrier concentration range for a few Si-Ge alloy compositions. The experimental data represented as scatter plot, taken from Ref. [33, 34, 35, 36, 37, 38, 39, 40]

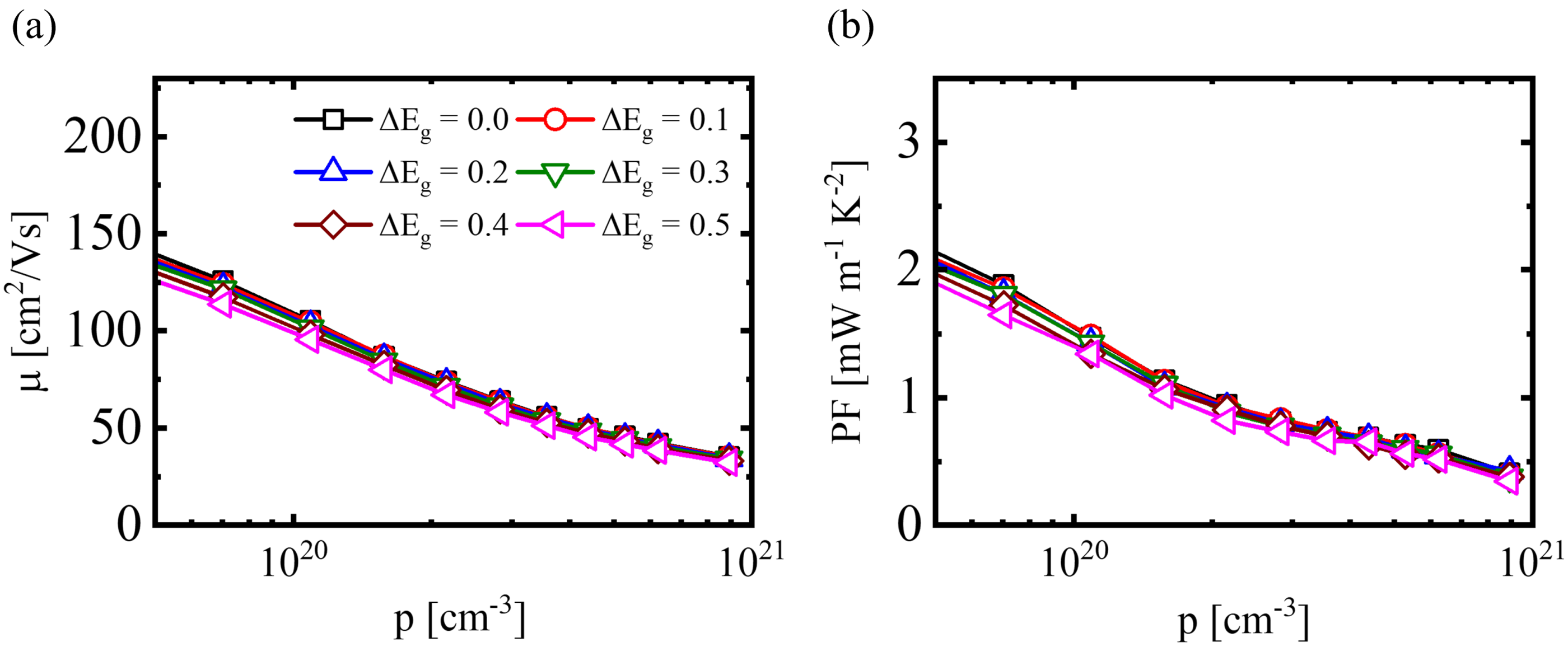

Figure S4: (a) Mobility, and (b) PF for 20 % Ge alloying in SiGe, with ΔE_g variation from 0 to 0.5 eV, as a function of carrier concentration (high carrier density) at 300 K. Insignificant variations are observed at high densities.

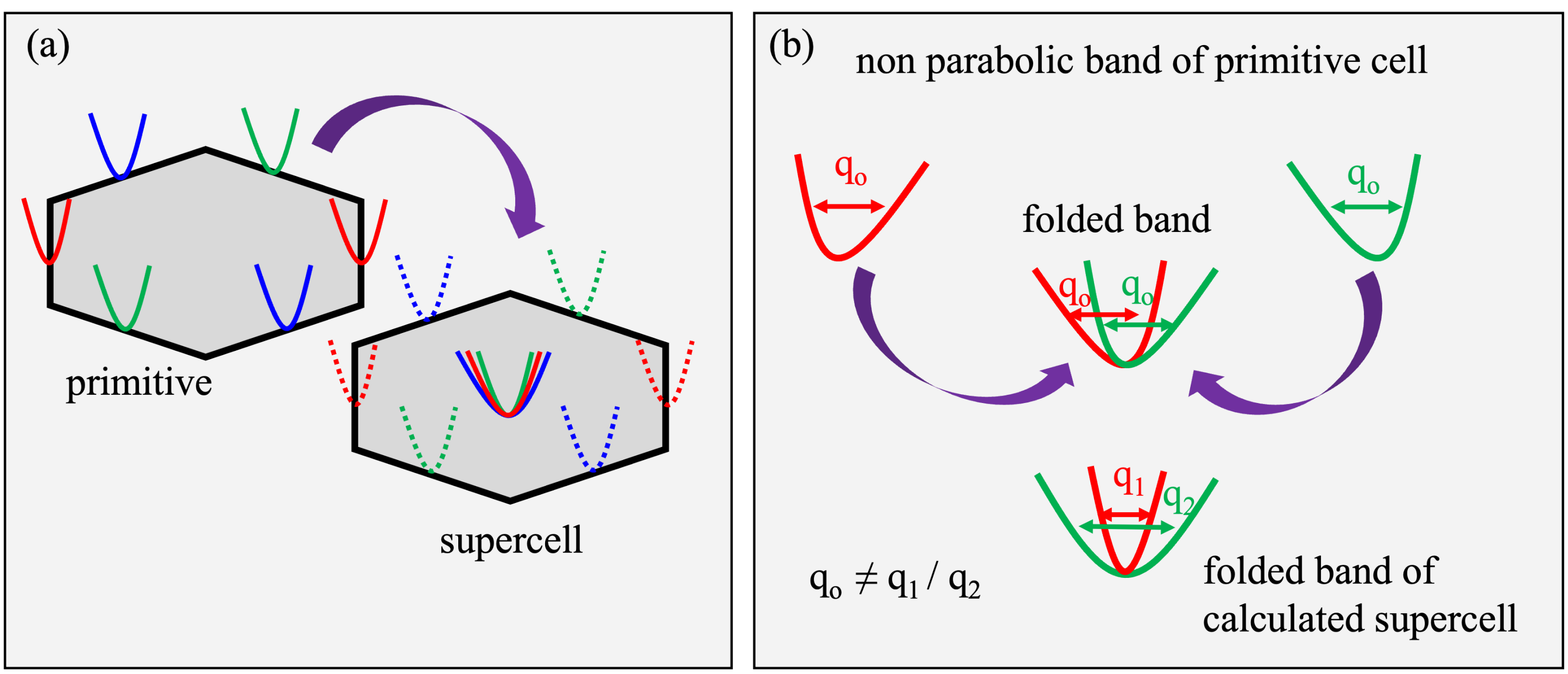

Figure S5: (a) Schematic of band folding from a primitive BZ to a folded BZ of a supercell for isotropic bands. (b) Folding of anisotropic bands from the primitive BZ to the supercell BZ. In the latter, band mixing will occur.

Scattering treatment, choice of supercell and associated error in calculated PF:
Below we explain our treatment of intra-/inter-valley/band scattering. In all alloy cases, we map the the scattering treatment to that of the pristine material treatment. In the case of ADP and ODP scattering, we extract effective single value deformation potentials across all bands and valleys using *ab initio* extracted matrix elements, which include all relevant selection rules and wavefunction overlap integrals, thus these values are used for the alloy supercells as well, and no issue arises for intra-/inter-valley/band treatment. The full formalism is described in Ref. [2]
Issues arise in the case of POP and IIS, for which the k-space information is lost upon folding in supercells, and the pristine overlap integral squared ($\langle I^2_{kk'} \rangle$) information needs to be explicitly employed. The treatment we follow for IIS and POP is as follows:
In the case of XNiSn-type systems, the CB has only one band (consisting of 6 equivalent X-valleys). In the pristine material the overlap integrals $I^2_{kk'}$ between all states in the same valley or other equivalent valleys is calculated to be very close to unity. Thus for pristine unit cell calculations we use $\langle I^2_{kk'} \rangle = 1$ for all transitions. However, in the pristine case, IIS and POP do not favor inter-valley scattering, because their strength reduces strongly for states farther away from each other in the BZ. Thus, effectively only intra-valley scattering is feasible for IIS and POP. For fcc and 2×2×2 supercells, where the valleys collapse on the same k-space point, in order to mimic this effective inter-valley scattering suppression, we only allow for intra-valley (band) scattering alone.
For the VB of XNiSn there are 3-bands at the Γ-point, and the overlap integrals deviate from unity both for intra- and inter-band transitions. In fact, it turns out that for both intra- and inter-band the squared overlap values averaged near the band edges average at $\langle I^2_{kk'} \rangle = 0.333$ [1]. We compute accurately all overlap integrals for many states, but for computational convenience we average the squared values around the relevant transport energies and use a single values for all transitions.
The CB of TaFeSb follows the same unity overlap as XNiSn
Ideally one should be able to use any supercell and obtain the same transport result, as long as the atomic arrangement is the same and the mean-free-path of carriers is larger than the size of the supercell. However, different supercells fold the band structure differently, which can lead to band mixing, or valleys placed nearby or on top of other valleys. Essentially the k-space placement information is different for different supercells. This presents a fundamental difficulty in describing scattering rates, especially for the strong IIS and POP, which are anisotropic and they depend on the momentum exchange vector $\mathbf{q} = |\mathbf{k} - \mathbf{k'}|$. Thus, for a specific material we chose a specific supercell depending on its electronic structure, such that band mixing is avoided, and such that the resultant folded band structure retains as much of the original k-space valley separation. More specific details and illustrations for better understanding are shown below:
By creating a supercell, the bands on different high symmetry points in the primitive BZ are transformed onto new high symmetry points depending on the transformation matrix used to create the supercell.
A cartoon representing such folding is shown in Figure S5. In case of XNiSn, the conduction band minima is at the X-point as shown in Figure S6a. Let us now consider 3 cases of supercells: a conventional fcc, a 2×2×2 and an orthorhombic supercell. In the cases of the fcc and 2×2×2 supercells, the band at the X-point (triply degenerate) will be folded onto the Γ-point as shown in the cartoon Figure S6b, top panel. For simplicity only two valleys (X_1 and X_2) are shown. Since these bands are anisotropic, there is band mixing (Fermi surface mixing), which cannot be avoided, as it is fundamental to the DFT calculation. The two resultant folded bands are now not the same as those in the primitive BZ, thus, will have different exchange vectors $|\mathbf{k} - \mathbf{k'}|$ in scattering, which introduces error in the PF, as shown in Figure S6c. The only way to avoid this, is to choose a supercell where the folding does not happen at the same high symmetry point, which can be achieved with the orthorhombic structure. Figure S6b (middle panel) shows that the ideal and folded DFT calculated band structures are the same, and the band mixing issue does not appear: this provides almost perfect agreement for the PF between the pristine and the folded orthorhombic structure BZ bands (Figure S6c, second panel). On the other hand, such band mixing problem will not arise for Γ-centred band maxima, as this point is invariant to any transformation. In this case any supercell would provide identical transport results to the pristine unit cell band

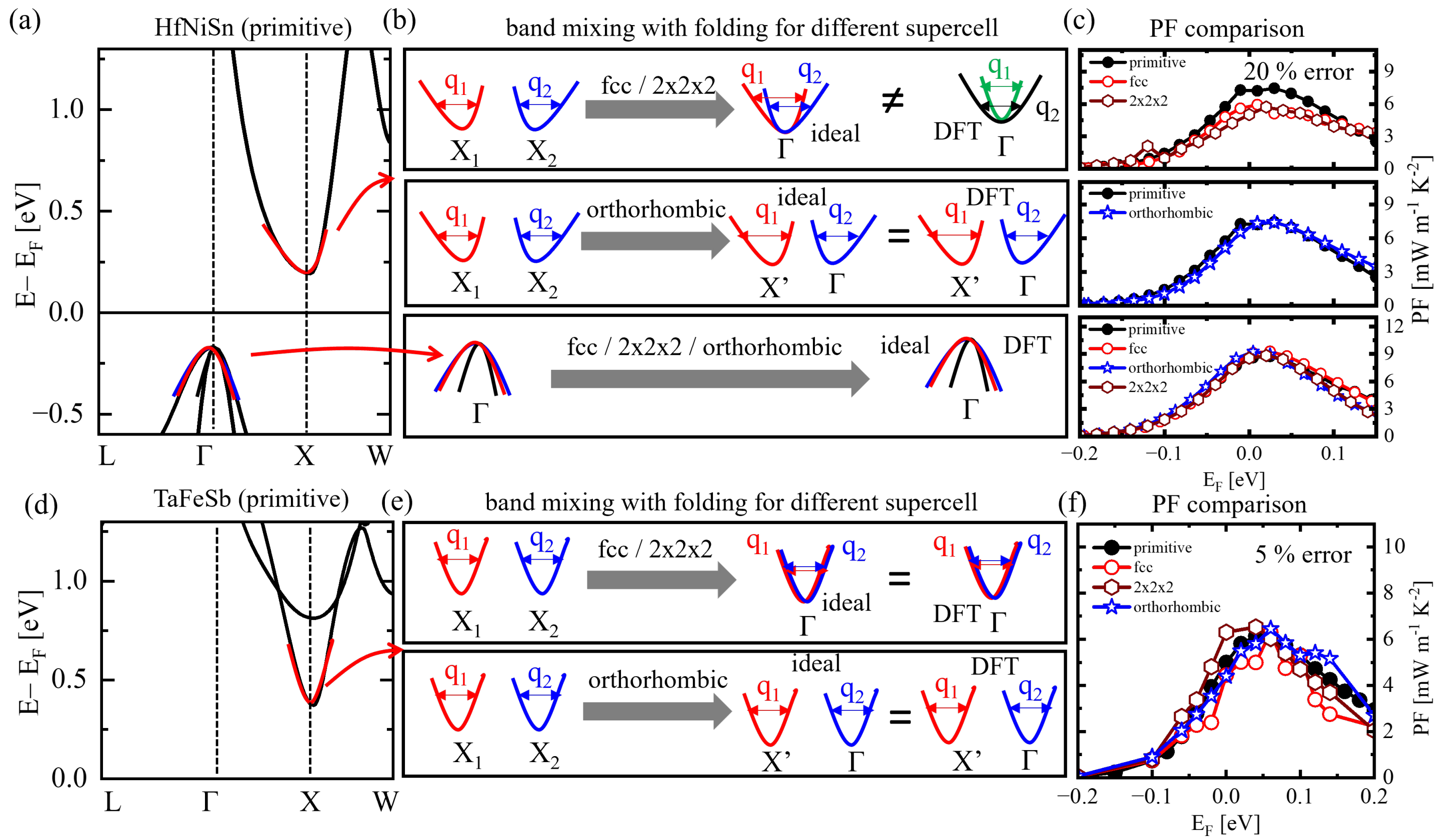

Figure S6: (a) Band structure of primitive HfNiSn with asymmetric conduction band (CB) minimum at the X-point. (b) Folding of the CB from the X-point to the Γ-point when a conventional fcc or a 2×2×2 supercell is considered (top panel), folding of the CB from the X-point to another X-point and Γ-point when an orthorhombic structure is considered (middle panel), and symmetry invariant Γ-centred VB maxima remains unchanged for any supercell (bottom panel). (c) Comparison of the PF from supercells with the PF primitive unit cell calculations. (d) Band structure of the primitive TaFeSb with a mostly symmetric conduction band (CB) minim at the X-point. (e) Folding of the CB from the X-point to the Γ-point when a conventional fcc or a 2×2×2 supercell are considered (top panel), folding of the CB from the X-point to another X-point and Γ-point when an orthorhombic structure is considered(bottom panel). (f) Comparison of the supercell obtain PF with the primitive cell obtained PF.

structure, irrespective of supercell size and symmetry as shown in Figure S6b, c (bottom panel).
A similar argument for the X-valley folding holds for TaFeSb as well in Figure S6e. However this material is more isotropic, thus, unlike HfNiSn, the effect of band mixing will be negligible when either a conventional fcc, or a 2×2×2, or an orthorhombic supercell is used. In this case, the use of any supercell will result in the same (or very similar) PF as that of the primitive structure (Figure S6e,f). Only a slight disagreement (5 %) in the PF in Figure S6f is observed due to the much smaller anisotropy. The scattering treatment for VB of TaFeSb is more complex and is discussed in the later section.

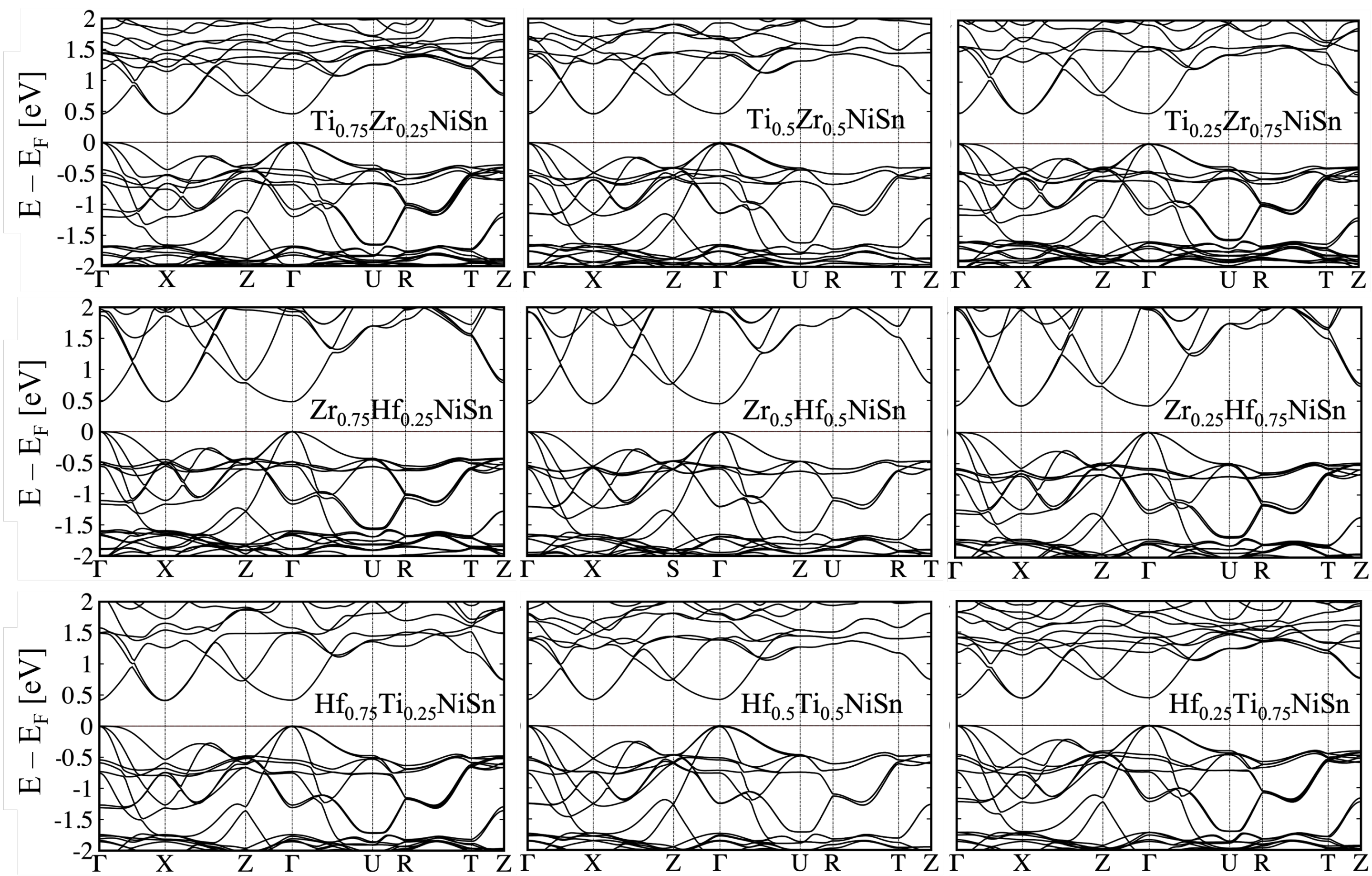

Figure S7: Electronic band structure of all the compositions considered in the main paper (as mentioned on each sub-figure) in orthorhombic phase.

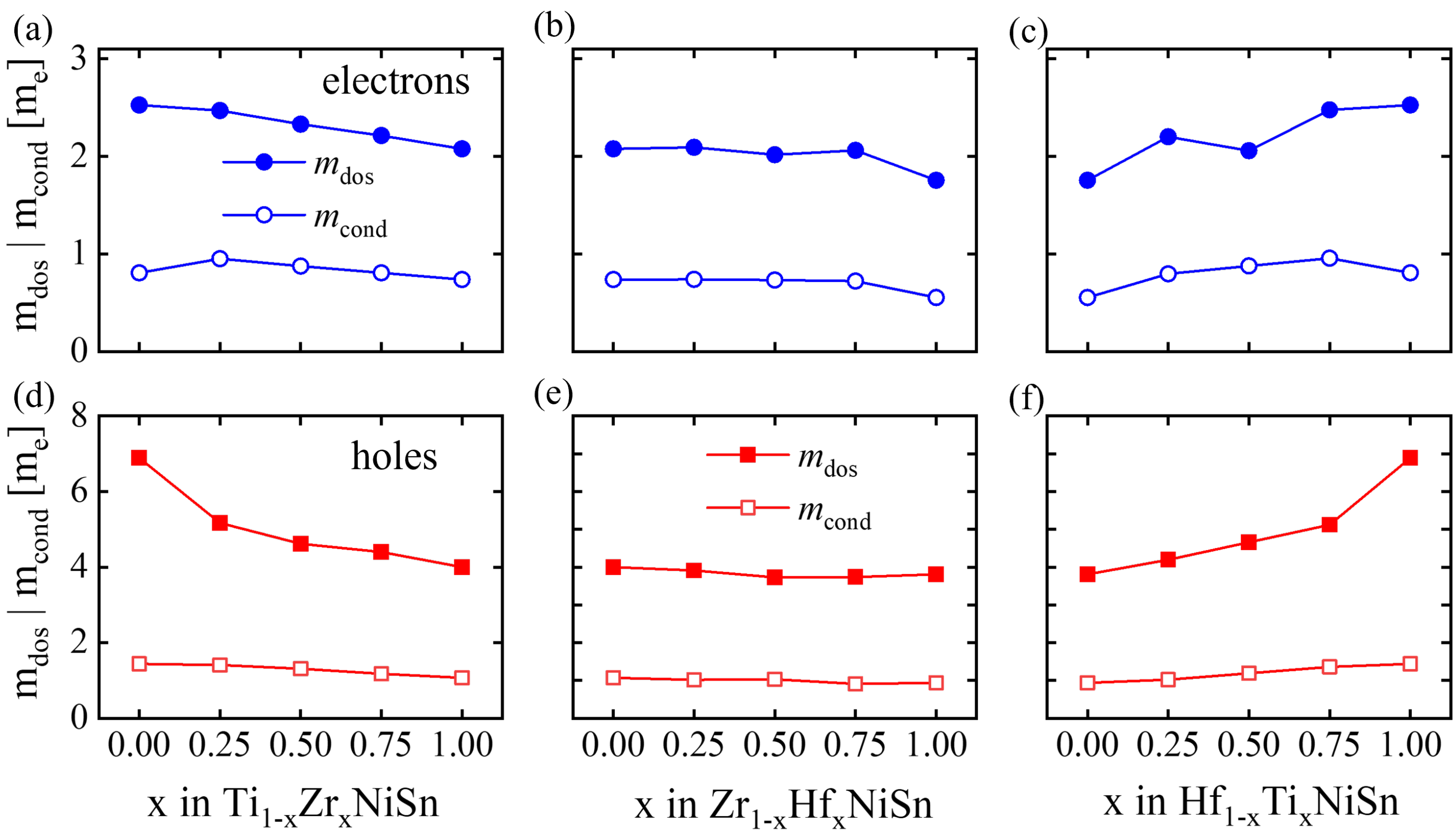

Figure S8: Density of states effective mass (m_{dos}) and conductivity effective mass (m_{cond}) of $\mathrm{Ti}_{1-x}\mathrm{Zr}_x\mathrm{NiSn}$, $\mathrm{Zr}_{1-x}\mathrm{Hf}_x\mathrm{NiSn}$, and $\mathrm{Hf}_{1-x}\mathrm{Ti}_x\mathrm{NiSn}$ for: (a-c) conduction band, and (d-e) valence band.

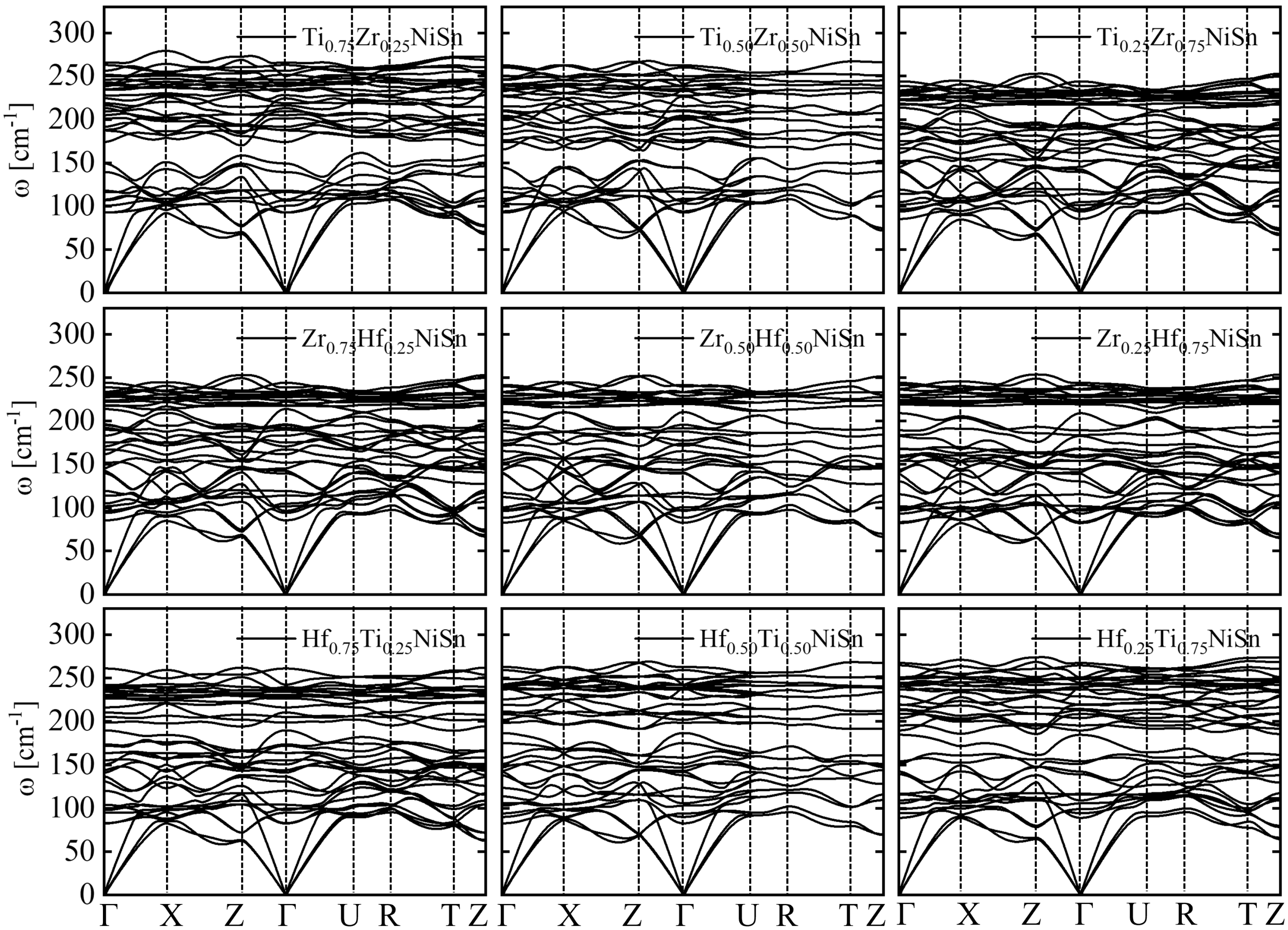

Figure S9: Phonon dispersion of all the compositions considered in the main paper (as mentioned on each sub-figure) in orthorhombic phase.

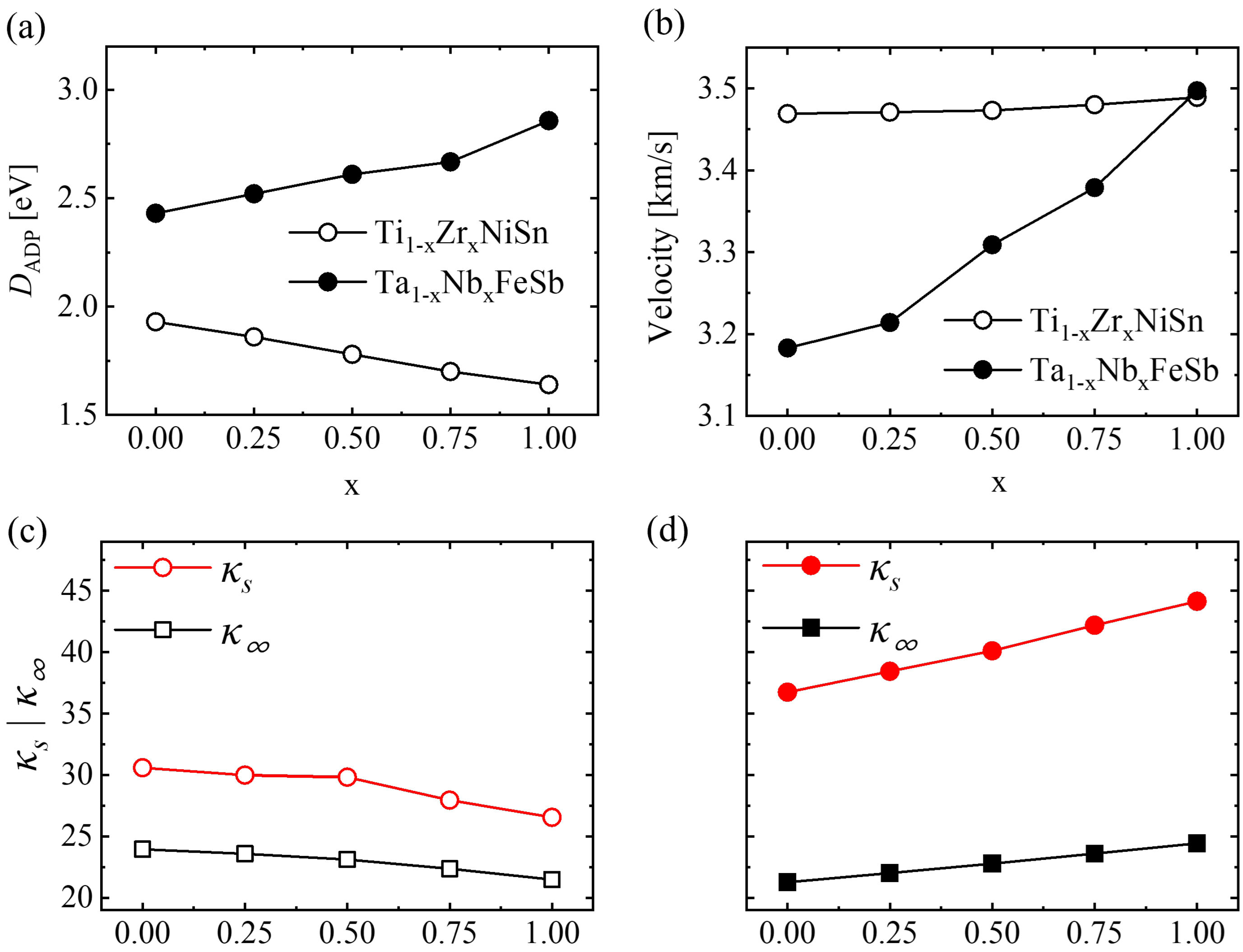

Figure S10: Calculated parameters for $Ti_{1-x}Zr_xNiSn$ and $Ta_{1-x}Nb_xFeSb$: (a) deformation potential trends, D_{ADP}, using the band shift method, (b) sound velocities, and (c, d) dielectric constants.

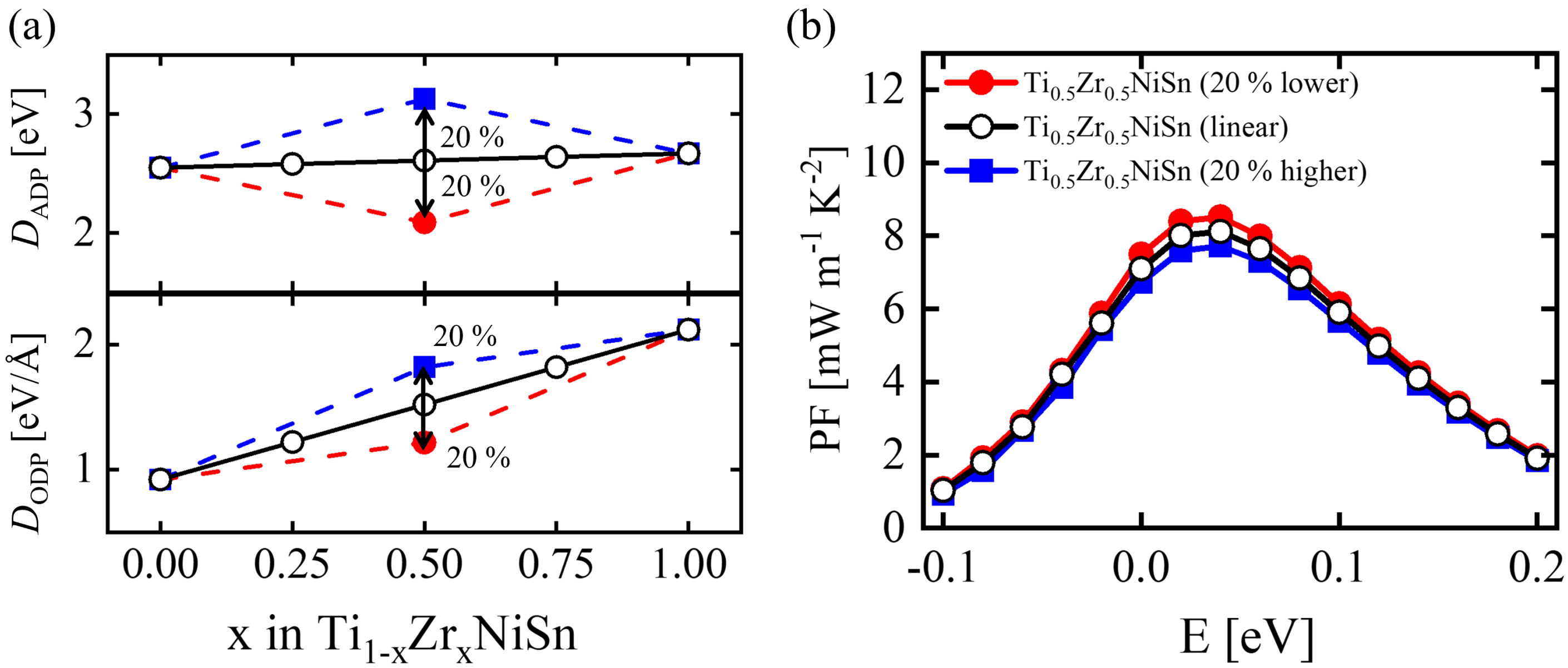

Figure S11: (a) Deviation from linear interpolated D_{ADP} and D_{ODP} values (by 20 %), and (b) the effect of such deviation on the PF of $Ti_{0.5}Zr_{0.5}NiSn$.

Alloy scattering comparison with IIS and POP for HH systems:
A comparison of alloy scattering with IIS and POP is done and is shown in Figure S12. The first row, Figure S12a,b,c, is the case for $Hf_{0.75}Zr_{0.25}NiSn$, and the second row, Figure S12d,e,f, is for $Ta_{0.75}Nb_{0.25}FeSb$. The three different columns show cases for carrier concentrations of n = 10^{18}, 10^{19}, and 10^{20} cm^{-3}. It is clear that in all cases the green lines, which show the combinations of IIS+POP have the highest scattering rate, with the blue lines that show IIS rates alone being very close, especially as the carrier density (and doping density) increases. The POP rates alone are shown by the red lines. The materials are polar, but not strongly polar, thus the POP lines are below the blue (IIS) lines for most cases. As the density increases, IIS becomes stronger, but POP suffers from screening, and its rates (red lines) decrease. Thus, with IIS increasing and POP decreasing, the overall IIS+POP (green lines) is almost invariant with density. The alloy scattering rates (black lines) are the lowest in general, certainly at least an order of magnitude lower compared to the IIS+POP (green lines). followed by the red lines, which are the POP scattering rates alone. However, as the density increases, and POP becomes more screened, the alloy and POP rates come closer together. The general trend, however, where alloy scattering is lower at such densities and can be ignored, remains.
Note, however, that the strength of the alloy scattering is determined by the alloy scattering potential ΔE_g used. In the case of the SiGe alloys we used $\Delta E_g = 0.2$ eV, which is the workfunction difference between the Si and Ge elements, but it also reproduces the experimental mobility at low densities. In the case of HHS the ΔE_g is not known, nor available reliable data for low densities (where IIS is negligible and cannot overshadow the alloy scattering rates) are available. However, following the same assumption as for Si-Ge, we considered the workfunction difference between Hf-Zr in the first case which is $\Delta E_g = 0.15$ eV, and in the second case that between Ta-Nb which is $\Delta E_g = 0.05$ eV.

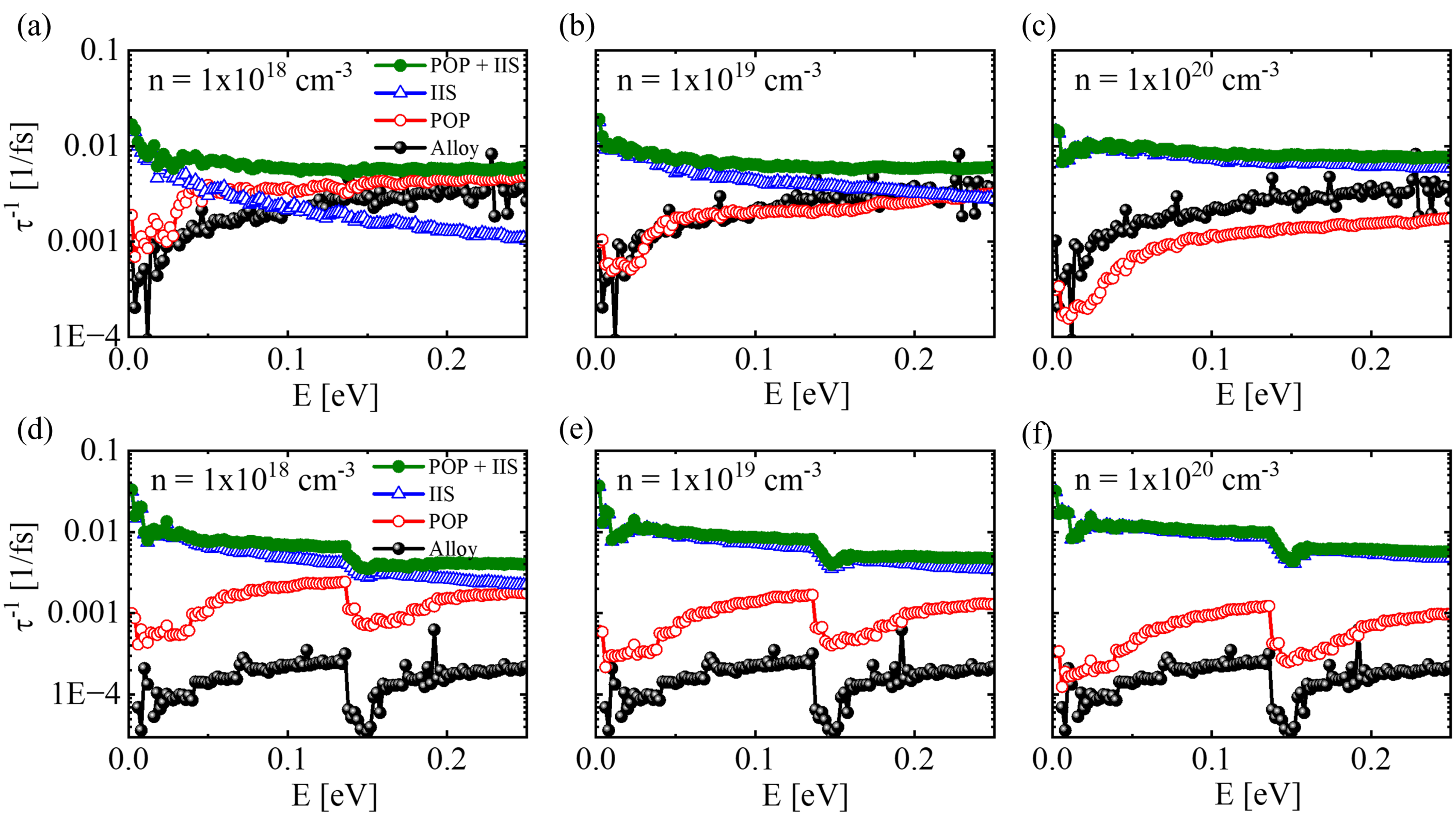

Figure S12: Scattering rate comparison between Alloy, POP, and IIS at carrier concentrations of 1×10^{18} cm^{-3}, 1×10^{19} cm^{-3} and 1×10^{20} cm^{-3} for: (a, b, c) $Hf_{0.75}Zr_{0.25}NiSn$ and (d, e, f) $Ta_{0.75}Nb_{0.25}FeSb$.

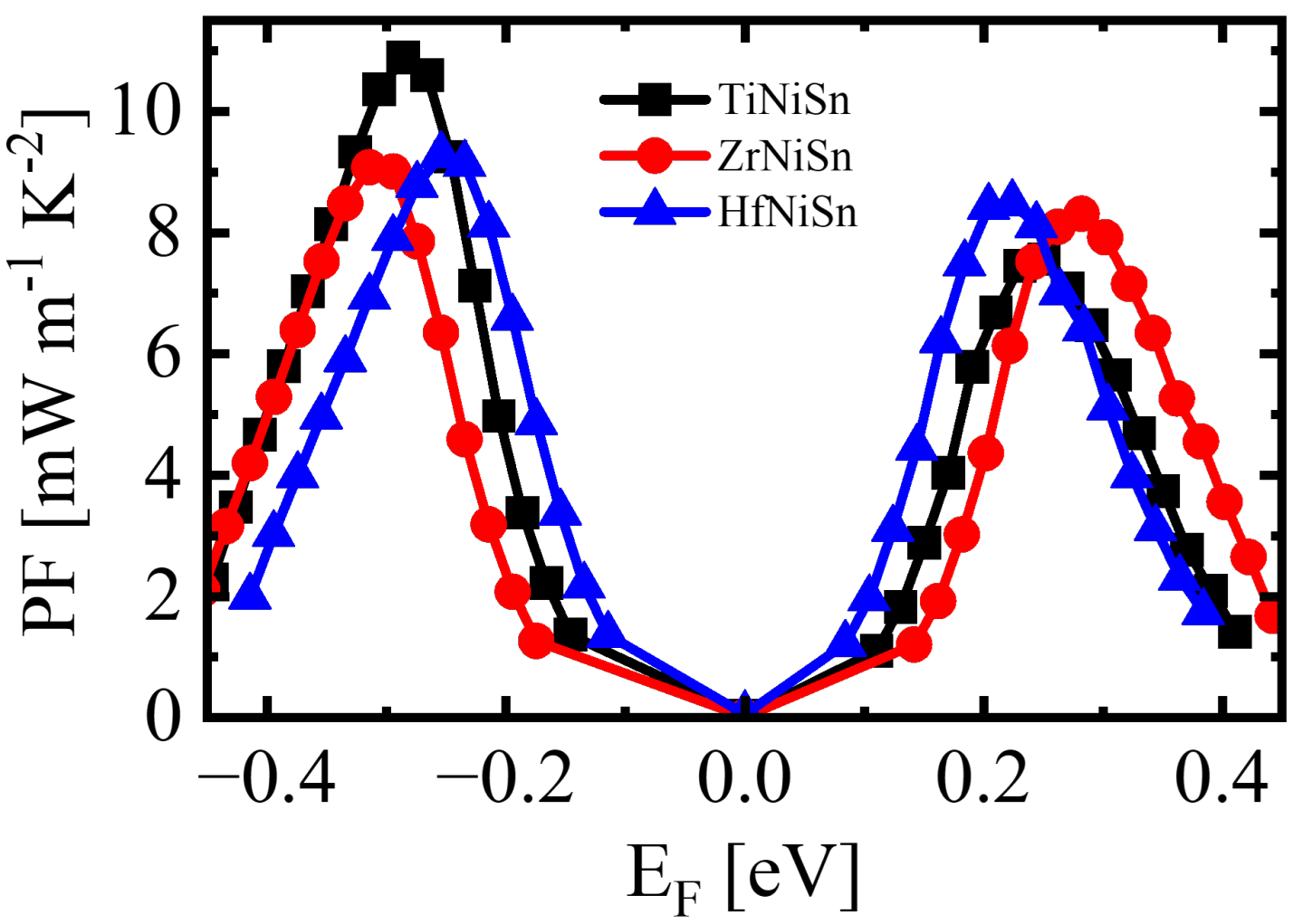

Figure S13: Power factor at 300 K versus Fermi energy for TiNiSn, ZrNiSn, and HfNiSn for both p-type (left side) and n-type (right side) polarities.

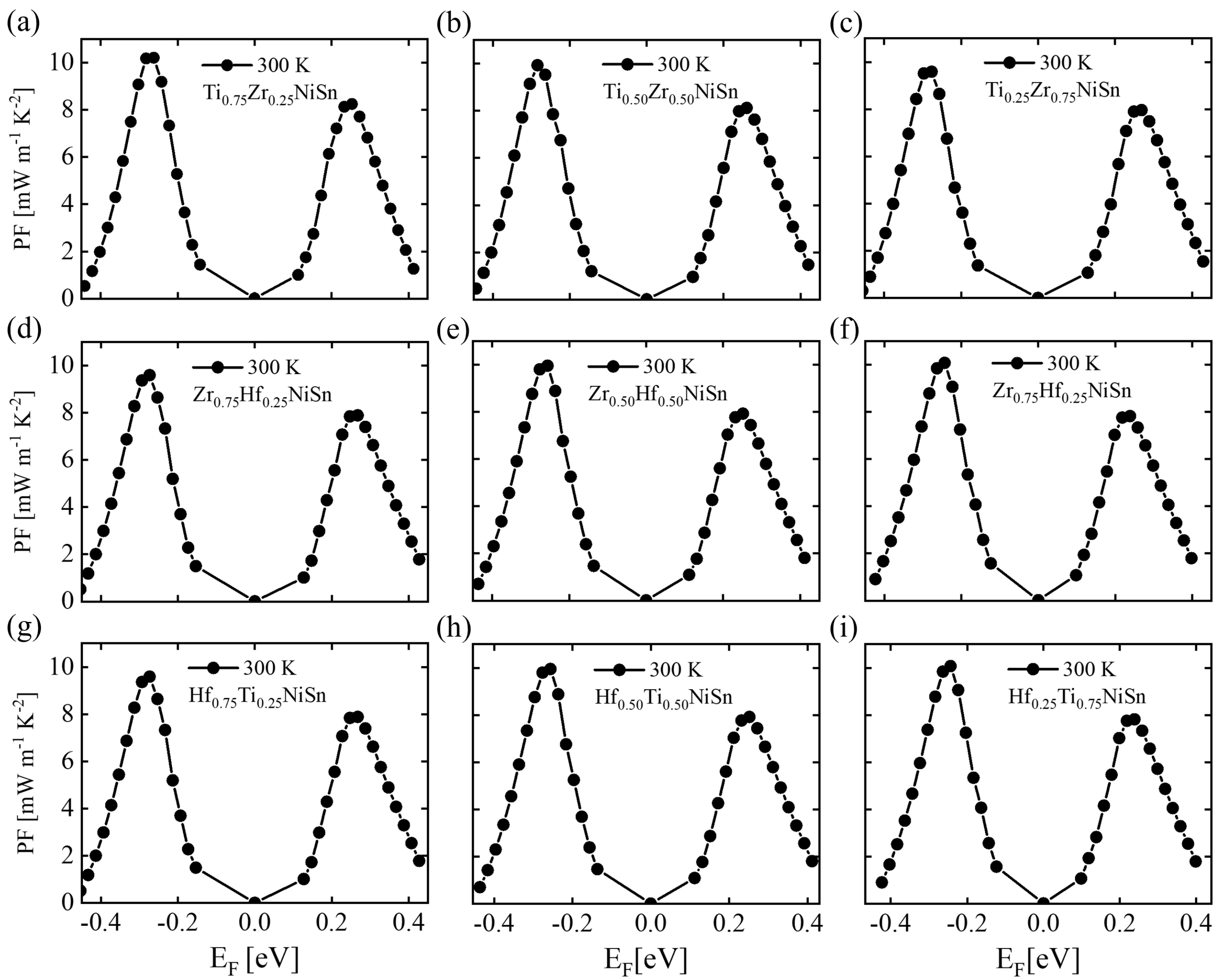

Figure S14: Power factor at 300 K versus Fermi energy for all the alloys considered, as noted in each sub-figure.

Bipolar transport calculation and its effect on PF:
We have considered FULL bipolar transport in XNiSn alloyed systems in which the band gap is small (and in all materials). Our calculation method integrates the TDF from E=-∞ to +∞, which includes simultaneously both the valence and conduction bands as:

$$\kappa_{el} = k_{\mathrm{B}}^2 T \int_{-\infty}^{+\infty} \Xi(E)\Big(-\frac{\partial f_0}{\partial E}\Big)\Big(\frac{E-E_{\mathrm{F}}}{k_{\mathrm{B}}T}\Big)^2 dE - \sigma S^2 T \tag{S11}$$

where the total Seebeck coefficient is computed fully numerically, but turns out to be close to $S = (\sigma_e S_e + \sigma_h S_h)/(\sigma_e + \sigma_h)$ as expected. Using this approximation, one can show that the total electronic thermal conductivity from the equation above can be split into the contribution from electrons (κ_e), holes (κ_h), and bipolar term (κ_{bi}) as:

$$\kappa_{el} = \kappa_e + \kappa_h + \kappa_{bi} \tag{S12}$$

where,

$$\kappa_e = k_{\mathrm{B}}^2 T \int_{E_F}^{+\infty} \Xi(E)\Big(-\frac{\partial f_0}{\partial E}\Big)\Big(\frac{E-E_{\mathrm{F}}}{k_{\mathrm{B}}T}\Big)^2 dE - \sigma_e S_e^2 T \tag{S13}$$

$$\kappa_h = k_{\mathrm{B}}^2 T \int_{-\infty}^{E_F} \Xi(E)\Big(-\frac{\partial f_0}{\partial E}\Big)\Big(\frac{E-E_{\mathrm{F}}}{k_{\mathrm{B}}T}\Big)^2 dE - \sigma_h S_h^2 T \tag{S14}$$

$$\kappa_{bi} = \frac{\sigma_e \sigma_h}{\sigma_e + \sigma_h}(S_e - S_h)^2 T \tag{S15}$$

This treatment includes the contribution of electrons (Equation S22) and holes (Equation S14) to the electronic thermal conductivity, as well as the bipolar thermal conduction term (κ_{bi}), the latter comes automatically in our computational method [41, 42, 43]. We also observe some bipolar effect on the PF even at 600 K, which becomes stronger at 900 K, as shown in Figure S15 for HfNiSn (band gap = 0.39 eV).At 900 K, PF can be reduced by 25 % because of bipolar transport.

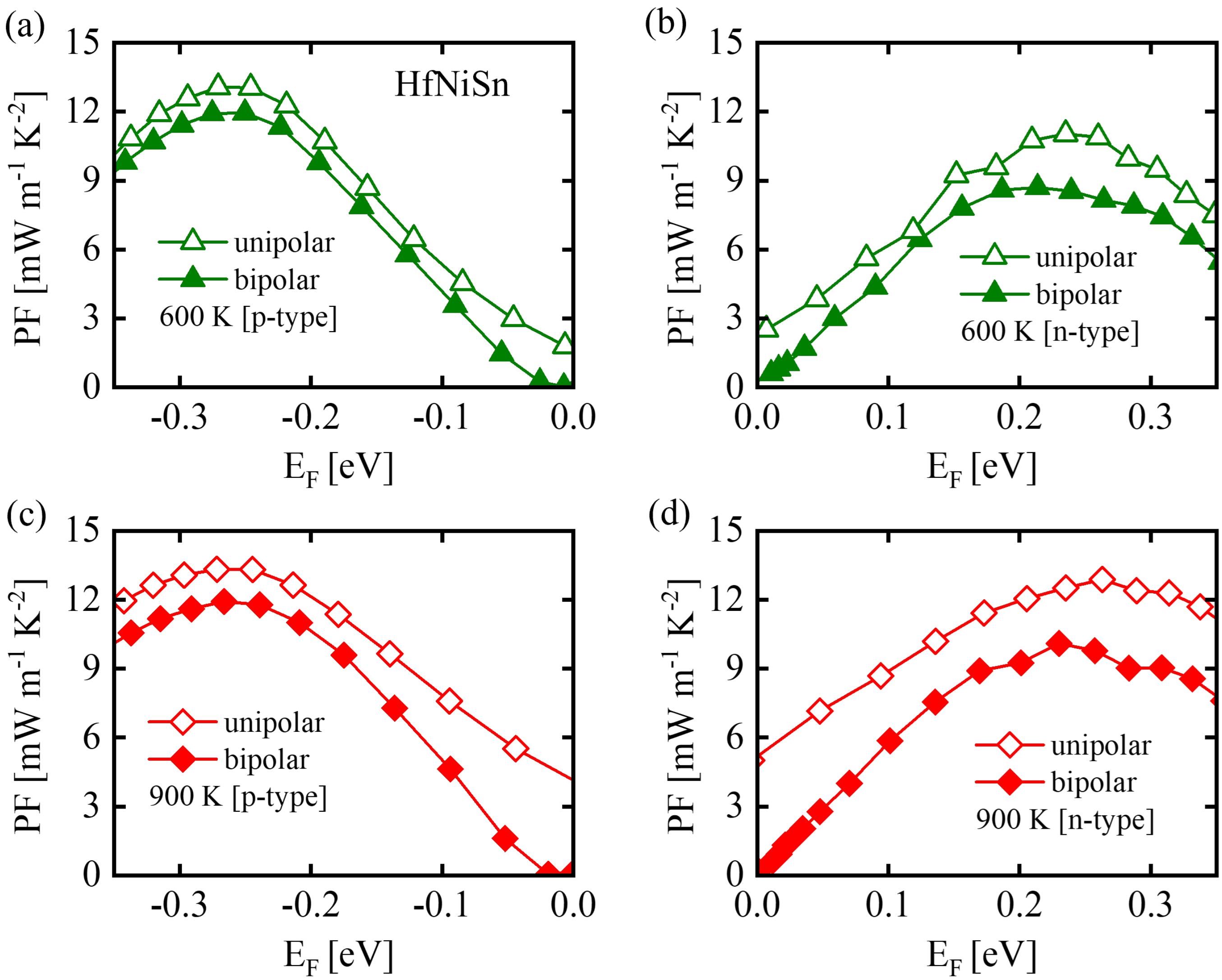

Figure S15: PF of HfNiSn at 600 K and 900 K with and without bipolar considerations.

Comparison with experiment at a fix carrier density:
A more systematic comparison of calculated data with experiment for a series of $Zr_{1-x}Hf_xNiSn$ alloys at a fixed carrier concentration is shown below in Figure S16. The experimental data is extracted from Ref. [44], and is more consistent in this case because of uniform synthesis conditions for all the compositions, which makes comparison more reliable (same grains, defects etc). Figures S16(a-c) show a comparison of the Seebeck coefficient, conductivity, and PF at the maximum reported PF in the range of 300 - 900 K. The carrier concentration is 4×10^{20} cm^{-3} for $Zr_{1-x}Hf_xNiSn$ for x = 0, 0.2, 0.4, 0.6, 0.8 and 1.0. Due to the presence of defects and disorder in these typical polycrystalline systems, the measured Seebeck is somewhat higher that the computed one, but the measured conductivity is around a factor of 2-3× reduced compared to computed values. Similar trends for the Seebeck coefficient and conductivity were also observed for NbFeSb [2]. Overall the measured PF remains lower than calculated by approximately a factor of 2×. Additionally, in Figure S16(d) below we show comparison with experimental zT. The magnitude of the reduction depends on the type of dopant and the quality of polycrystalline sample, but our simulations here indicate the ultimate limit that can be reached under ideal synthesis conditions for alloyed samples is somewhere at the middle of the alloyed composition. Under these conditions, the achievable zT can be as high as double to what is typically realized in experiment

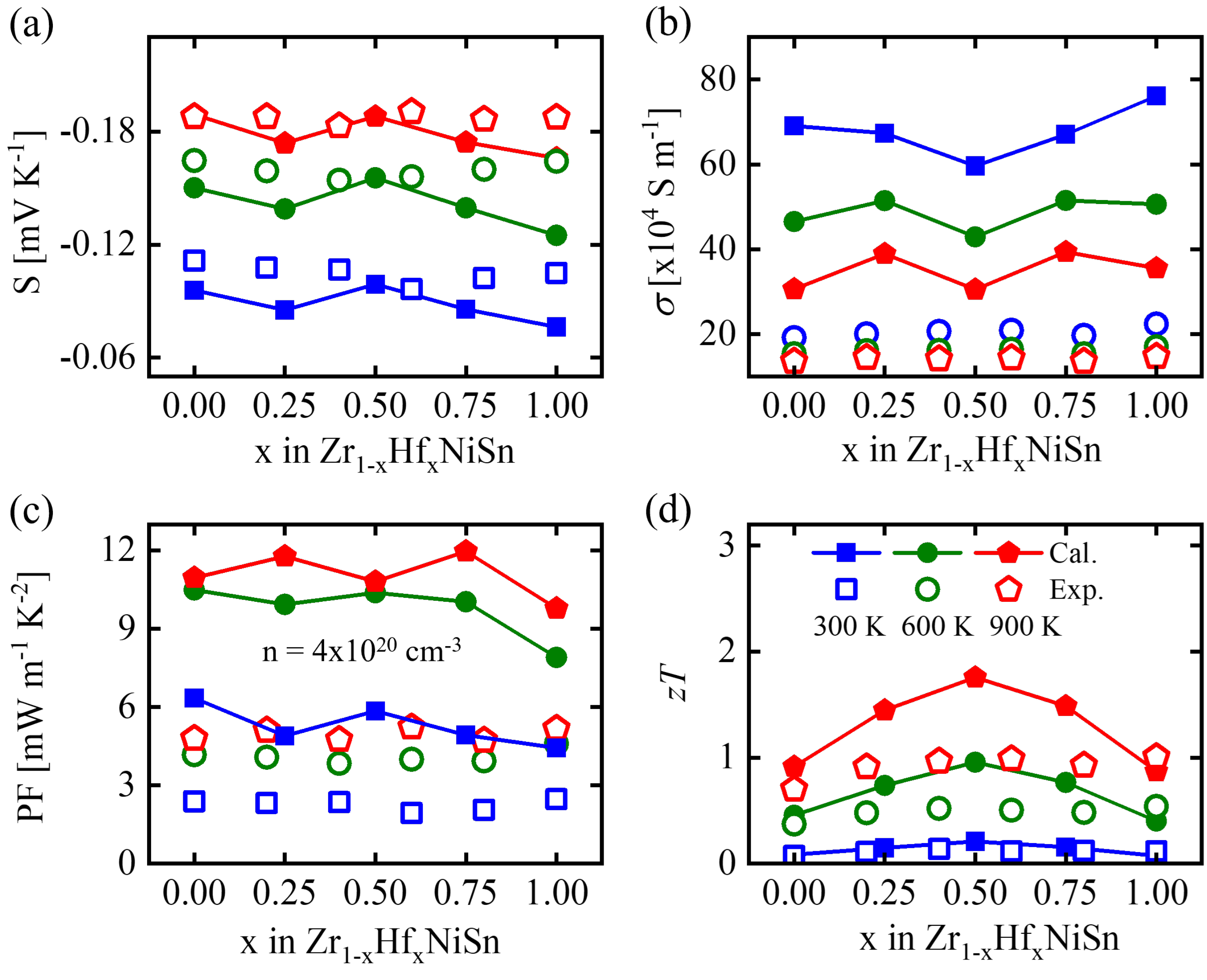

Figure S16: Calculated and experimental TE coefficients: (a) Seebeck coefficient, (b) electrical conductivity, (c) PF and, (d) zT of $Zr_{1-x}Hf_xNiSn$ at a carrier concentration of 4×10^{20} cm^{-3} at 300, 600 and 900 K. The experimental data is extracted from Ref. [44]..

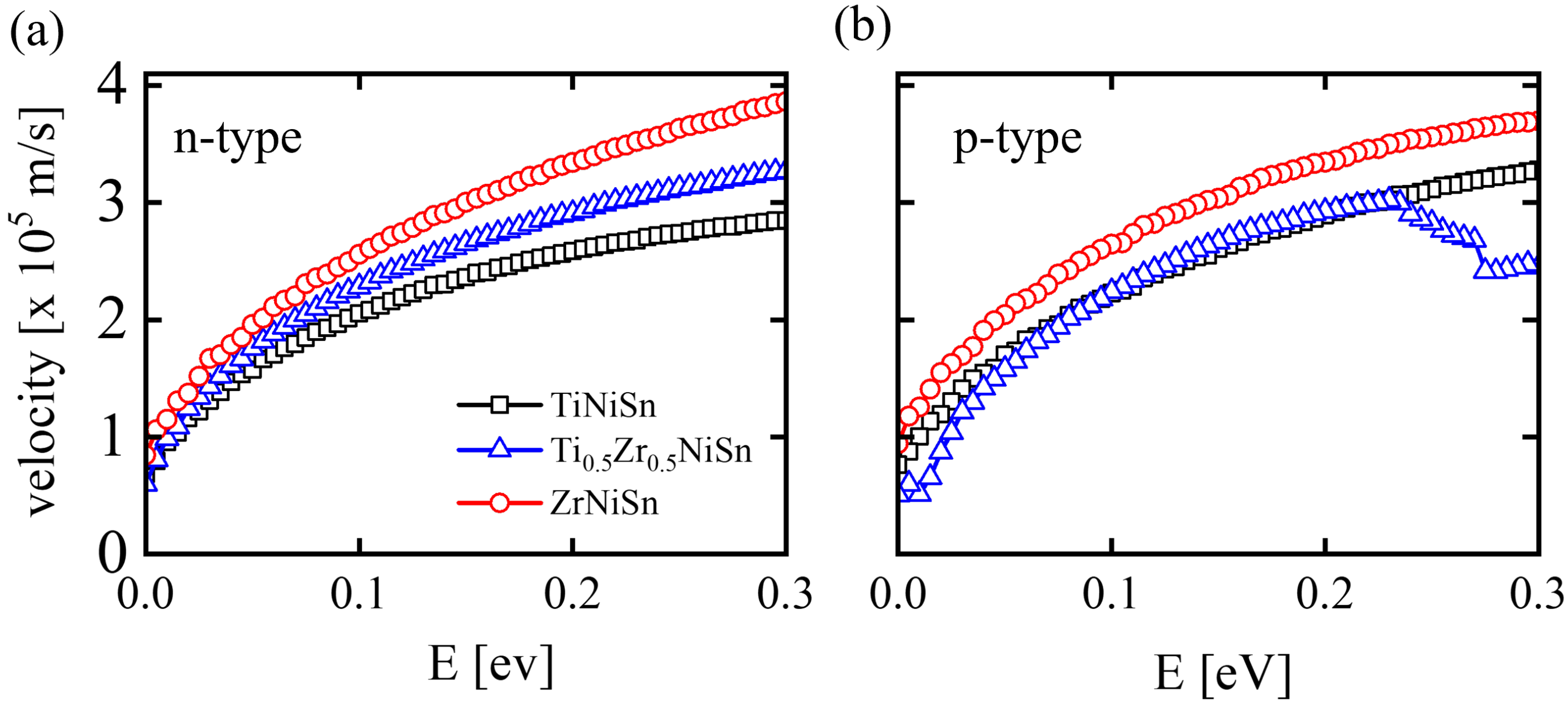

Figure S17: Comparison of band velocities for TiNiSn (black squire), $Ti_{0.5}Zr_{0.5}NiSn$ (blue triangle), and ZrNiSn (red circle) for: (a) n-type and (b) p-type.

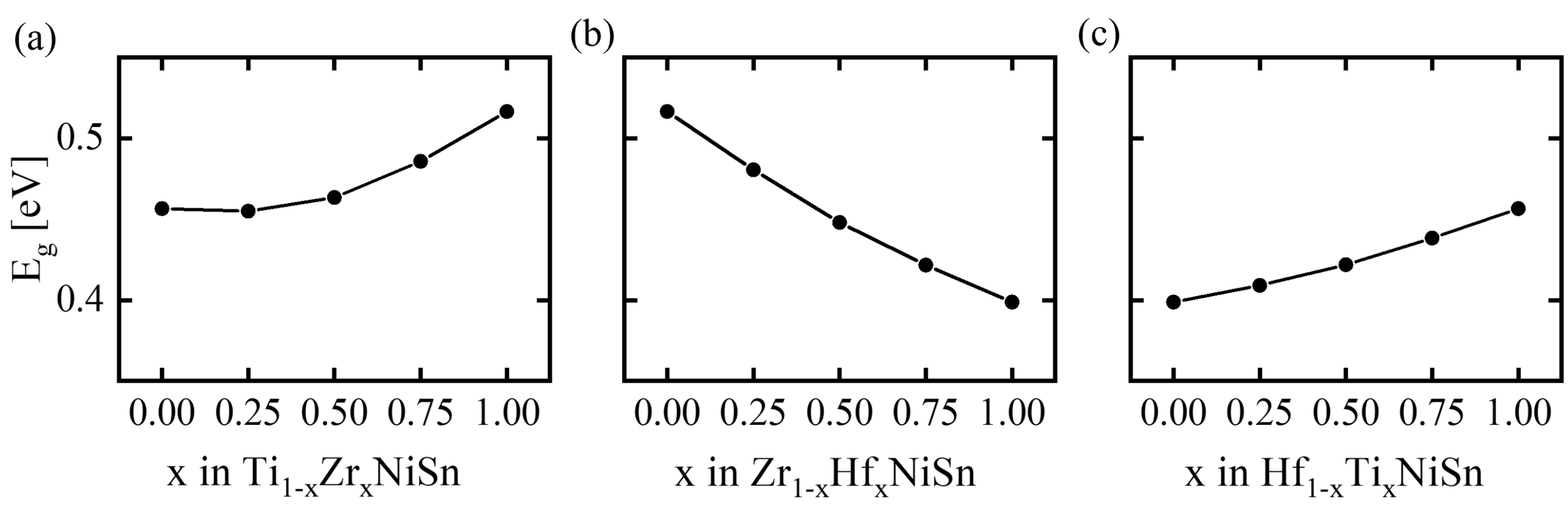

Figure S18: Effect of alloying on band gap of: (a) $Ti_{1-x}Zr_xNiSn$, (b) $Zr_{1-x}Hf_xNiSn$, and (c) $Hf_{1-x}Ti_xNiSn$ as a function of composition.

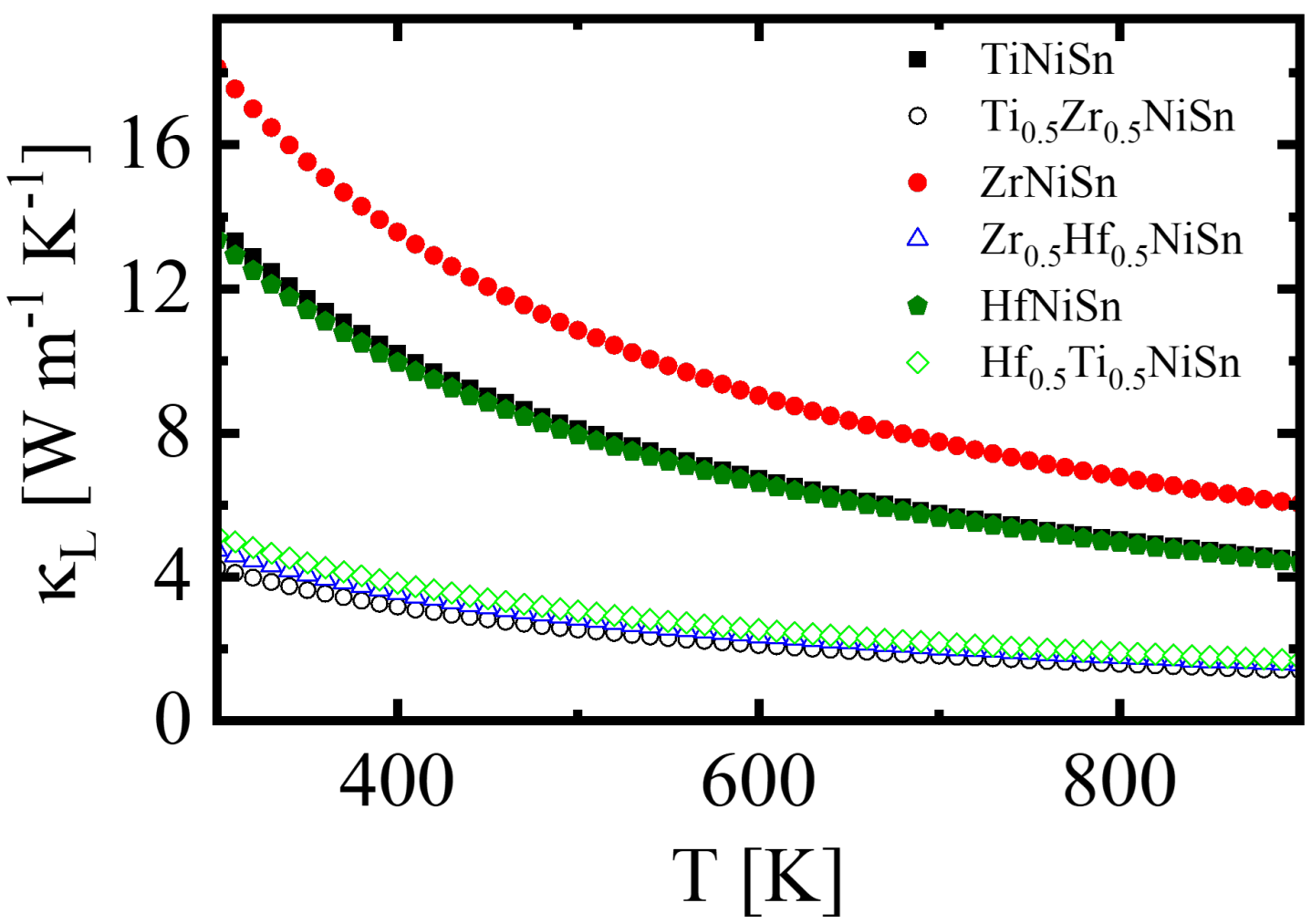

Figure S19: Lattice thermal conductivity versus temperature for pristine TiNiSn, ZrNiSn, and HfNiSn, and their alloys $Ti_{0.5}Zr_{0.5}NiSn$, $Zr_{0.5}Hf_{0.5}NiSn$, and $Hf_{0.5}Ti_{0.5}NiSn$.

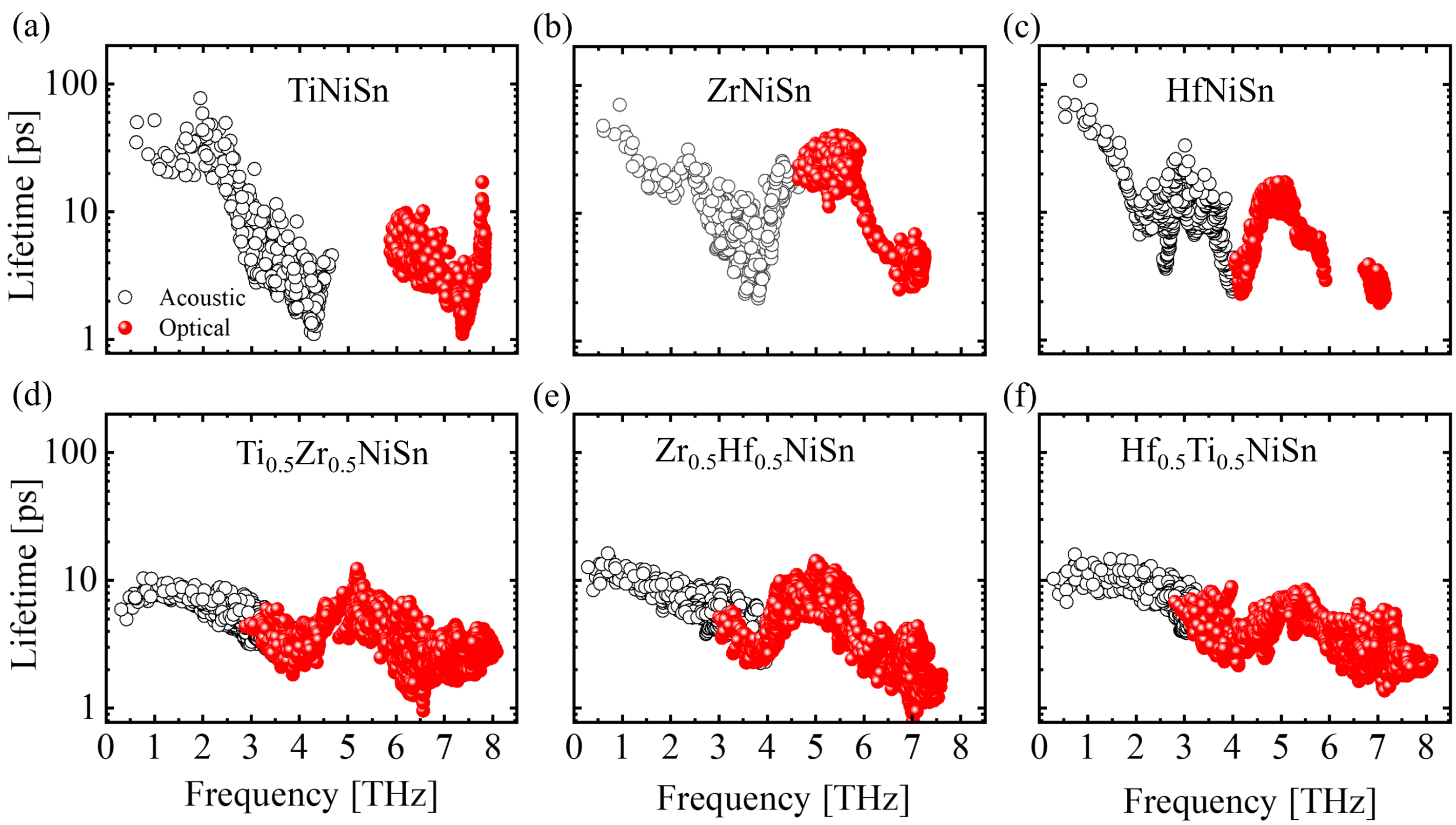

Figure S20: Phonon life time versus phonon frequency for pristine TiNiSn, ZrNiSn, and HfNiSn, and their alloys $Ti_{0.5}Zr_{0.5}NiSn$, $Zr_{0.5}Hf_{0.5}NiSn$, and $Hf_{0.5}Ti_{0.5}NiSn$. Black open-circles are the lifetimes of acoustic modes and red spheres for for optical modes.

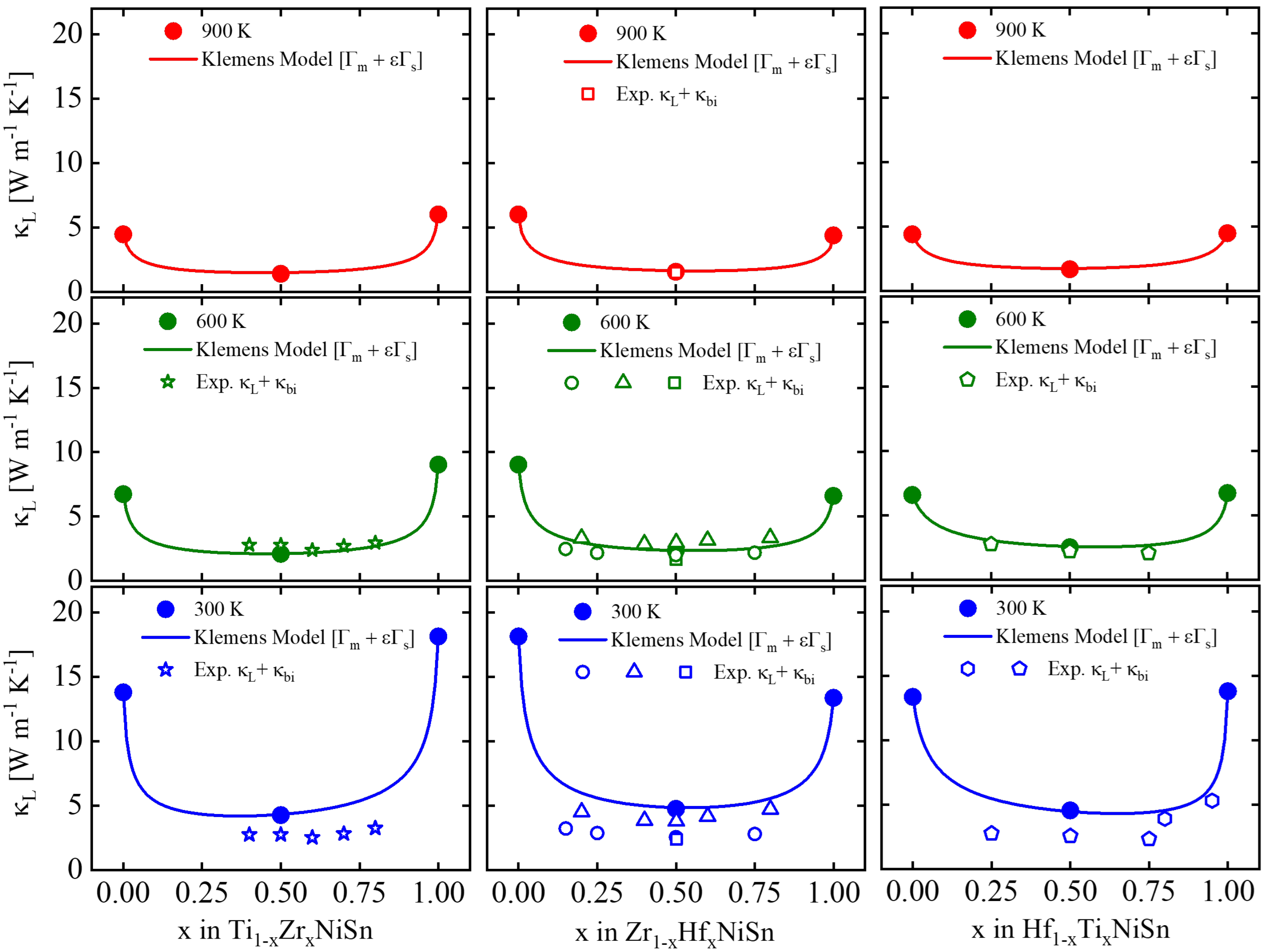

Figure S21: Lattice thermal coondutivity of $Ti_{1-x}Zr_xNiSn$, $Ti_{1-x}Zr_xNiSn$, and $Ti_{1-x}Zr_xNiSn$ for 300, 600 and 900 K. The filled data are calculated using Phono3Py, the line is the fitted using Klemens model by adjusting the disorder parameter ($\Gamma_m + \epsilon\Gamma_s$), and the open scatter points are experimental reported $\kappa_L + \kappa_{bi}$. The experimental data is for $Zr_{1-x}Hf_xNiSn$: Ref [45] (open circles) , Ref [44] (open triangles), [46](open squares); for $Ti_{1-x}Zr_xNiSn$: Ref [47] (open stars); and for $Hf_{1-x}Ti_xNiSn$: [48] (open pentagons) [49] (open hexagons).

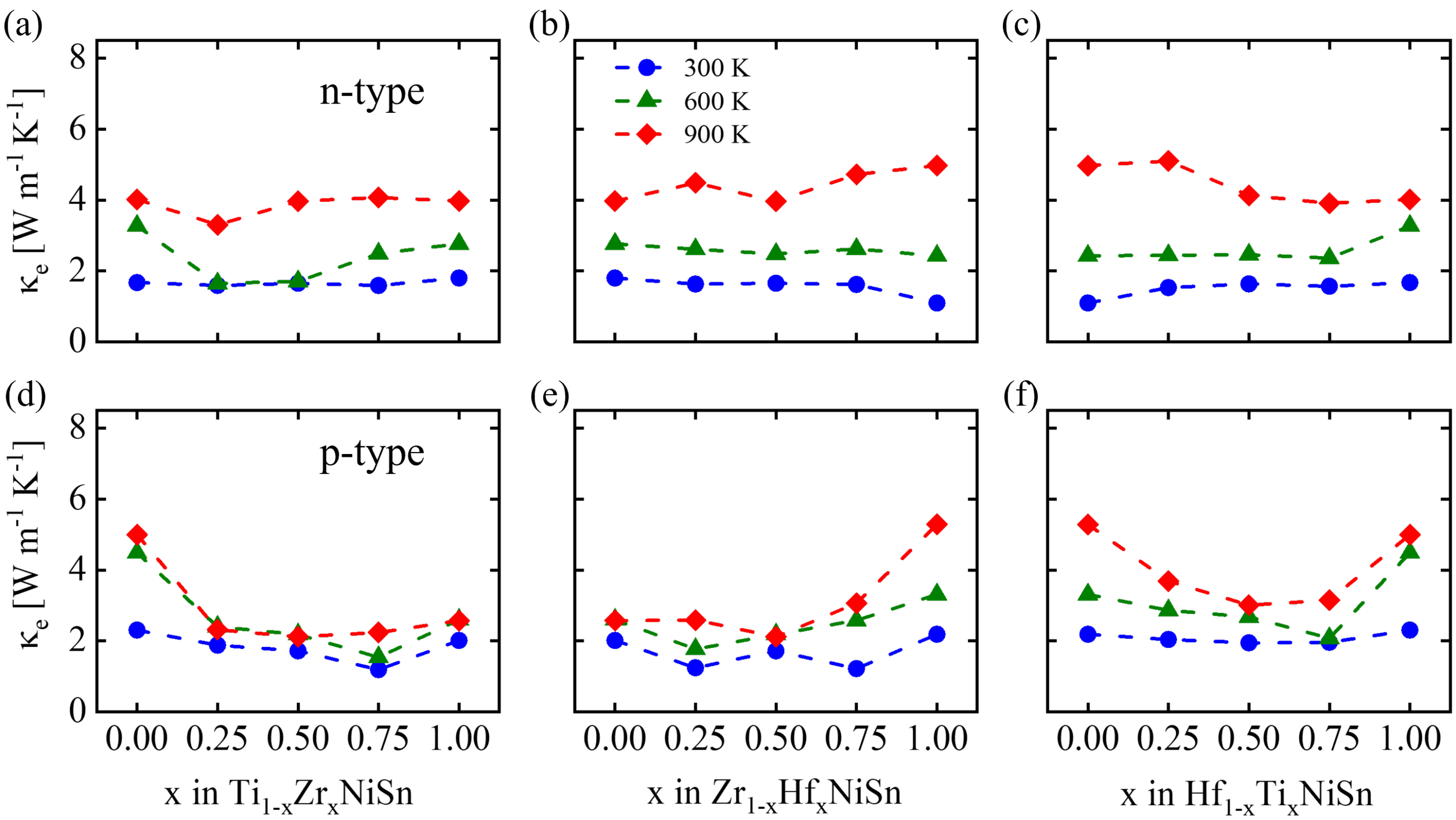

Figure S22: Electronic thermal conductivity at maximum PF for $Ti_{1-x}Zr_xNiSn$, $Zr_{1-x}Hf_xNiSn$, and $Hf_{1-x}Ti_xNiSn$ for: (a,b,c) n-type and, (d,e,f) p-type polarities, for 300 K, 600 K and 900 K.

Comparison of transport coefficient with experimental report for TaFeSb based HH:
In Figure S23, We have included a full comparison of the idealized calculated results to experimentally reported transport coefficients of similar alloyed systems (thus the comparison might not be fully fair, but useful, as it will reveal the differences between the PF and zT of pristine materials and their alloys). As in the case of the XNiSn system above, the experimental Seebeck coefficient is also higher here than the calculated one, but the experimental conductivity is significantly lower. The experimentally synthesized $Ta_{1-x}Ti_xFeSb$ is reported to have Ta/Ti-Fe antisite disorder, and together with grain boundaries would be the reason behind such disagreement [50]. Also, Ti substitution will lead to band splitting as discussed in the main text, which reduces the conductivity. Overall, the experimental maximum PF is around half of the computational values (as expected and observed in multiple studies [2, 14]).
The thermal conductivity and zT calculation and its comparison with the highest reported zT for TaFeSb-based HH is shown in Figures S23(d-e). In this case, because of the drastic reduction in κ_L in $Ta_{1-x}Ti_xFeSb$ (Figure S23d), the experimental zT becomes even higher than the calculated one for the ideal pristine TaFeSb system. Although he reduction in the PF because of disorder, defects and boundaries reduces the PF by 2× or 3× compared to pristine theoretical levels, the reduction in lattice thermal conductivity is much larger.

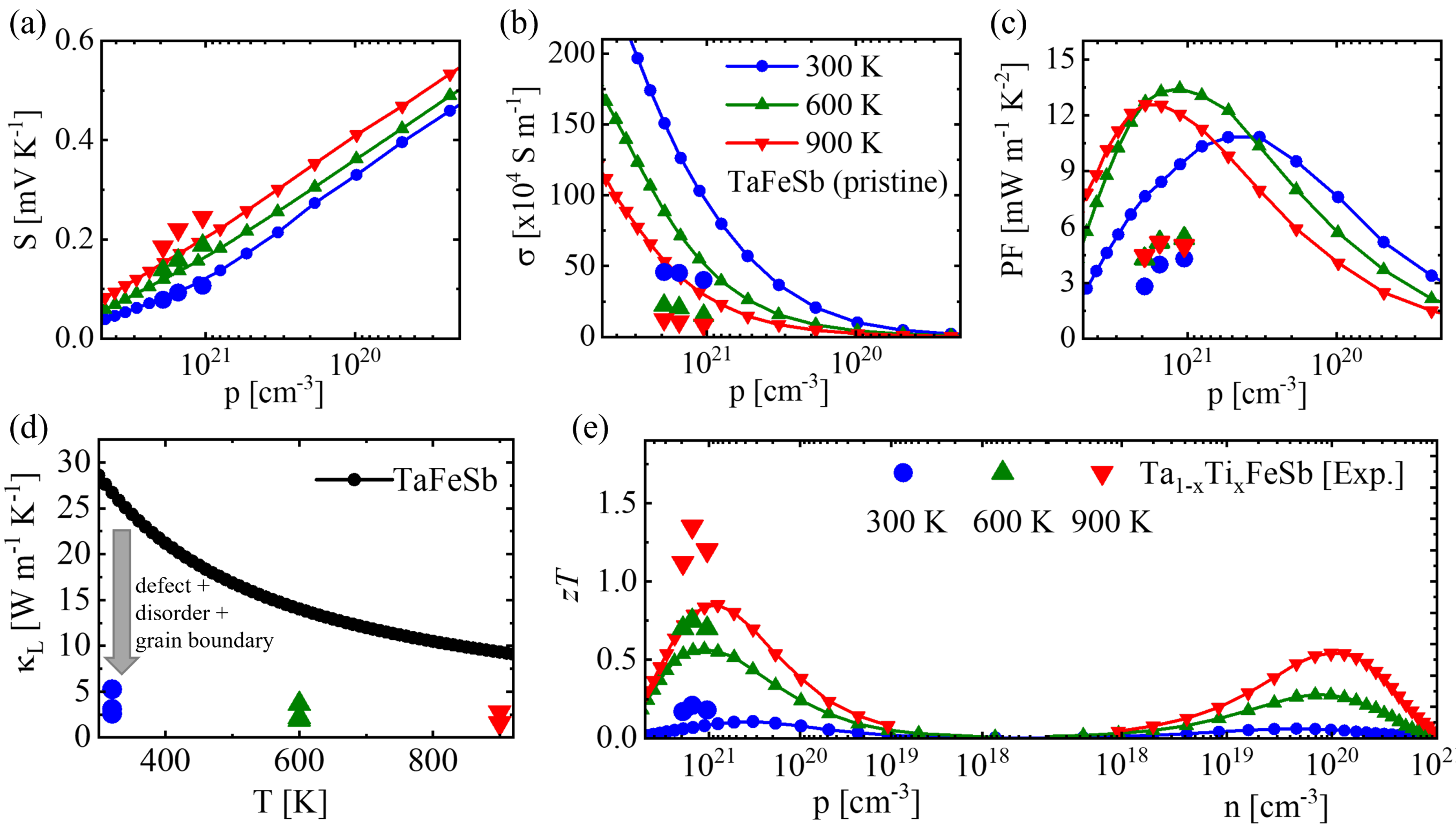

Figure S23: Calculated and experimental TE coefficients of p-type TaFeSb: (a) Seebeck coefficient, (b) electrical conductivity, and(c) PF. (d) Lattice thermal conductivity of TaFeSb as a function of temperature, (b) zT of TaFeSb as a function of carrier concentration for 300, 600 and 900 K. the experimental data of $Ta_{1-x}Ti_xFeSb$ is extracted from Ref [50].

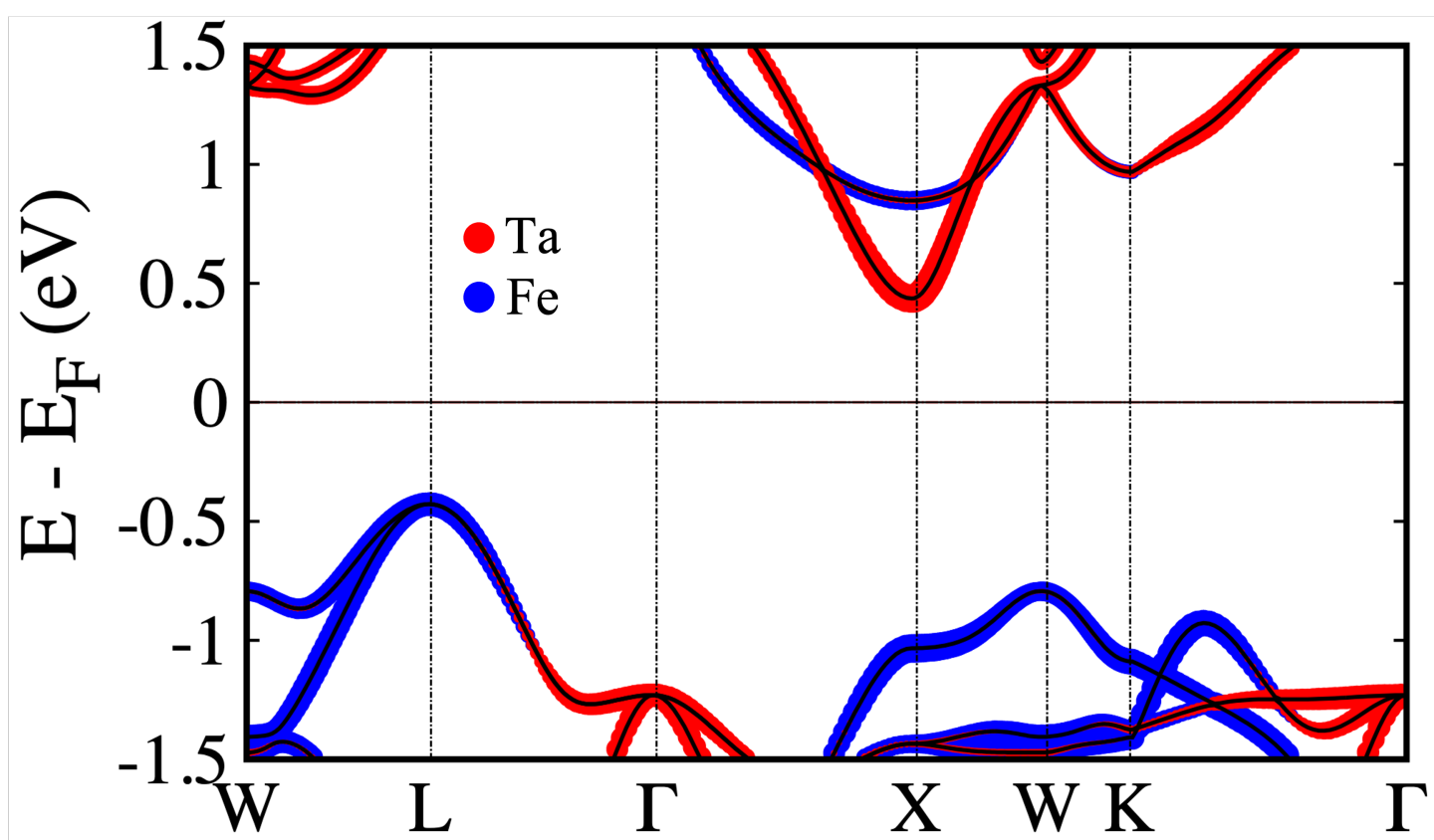

Figure S24: Atomic projected band structure for TaFeSb. Red and blue circles show the Ta and Fe atomic contributions in the electronic structure, respectively.

Band splitting quantification:
We have tried to fully pin-point to the physical reasons behind the band (valley) splitting; many more studies for different alloys are needed for generalization. However, we present below two examples that might provide some clues. In case of Nb alloying in TaFeSb, there is no size effect as both Nb and Ta have the same ionic radius, and thus the lattice parameter does not change with alloying. In this case, we observe that the splitting is dependent on the alloying concentration. Figure S25a shows a correlation between total band splitting ($\Delta E_1 + \Delta E_2$) and alloying concentration (here we sum up the splittings between both degenerate bands). It follows a C_1x(1-x) function where C_1 is a constant fitting parameter and x is the concentration of Nb in $Ta_{1-x}Nb_xFeSb$. The constant is not universal, but we indeed believe it the difference in electronegativity between Ta and Nb might be playing a role. The conduction band of TaFeSb is mostly consisting of the d-orbitals of Ta. Nb is more electronegative than Ta, which results in the Nb d-orbital having a lower energy. When Nb is substituted at Ta, the Nb d-orbital hybridizes with Fe and will bring the CB minima to a lower energy, leading to band splitting. But in order to fully qualitatively understand the constant C_1 and its relation with electronegativity, many more examples of such alloying will be needed, which would be the subject of future work.
In case of aliovalent doping, i.e. Ti/Zr/Hf in the place of Ta, we find a clearer correlation: it is the size difference of the substituting atom's size (ΔR) that is the determining factor of the band splitting. Figure S25b shows the correlation between that size difference and band splitting. It might be possible that in some cases both electronegativity difference and size difference both play important roles, but more work is needed for this.

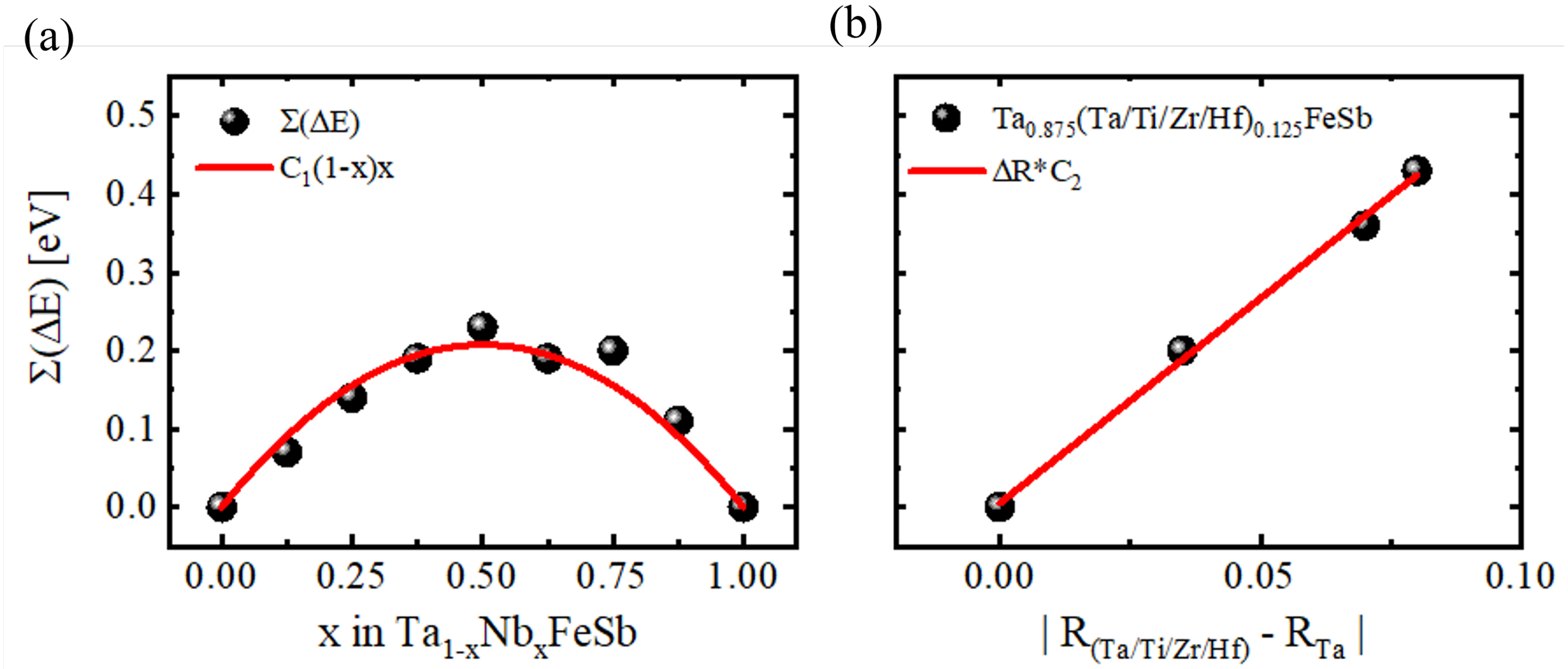

Figure S25: (a) Correlation of band splitting as a function of x in $Ta_{1-x}Nb_xFeSb$, (b) band splitting in TaFeSb when alloyed with Ti/Zr/Hf.

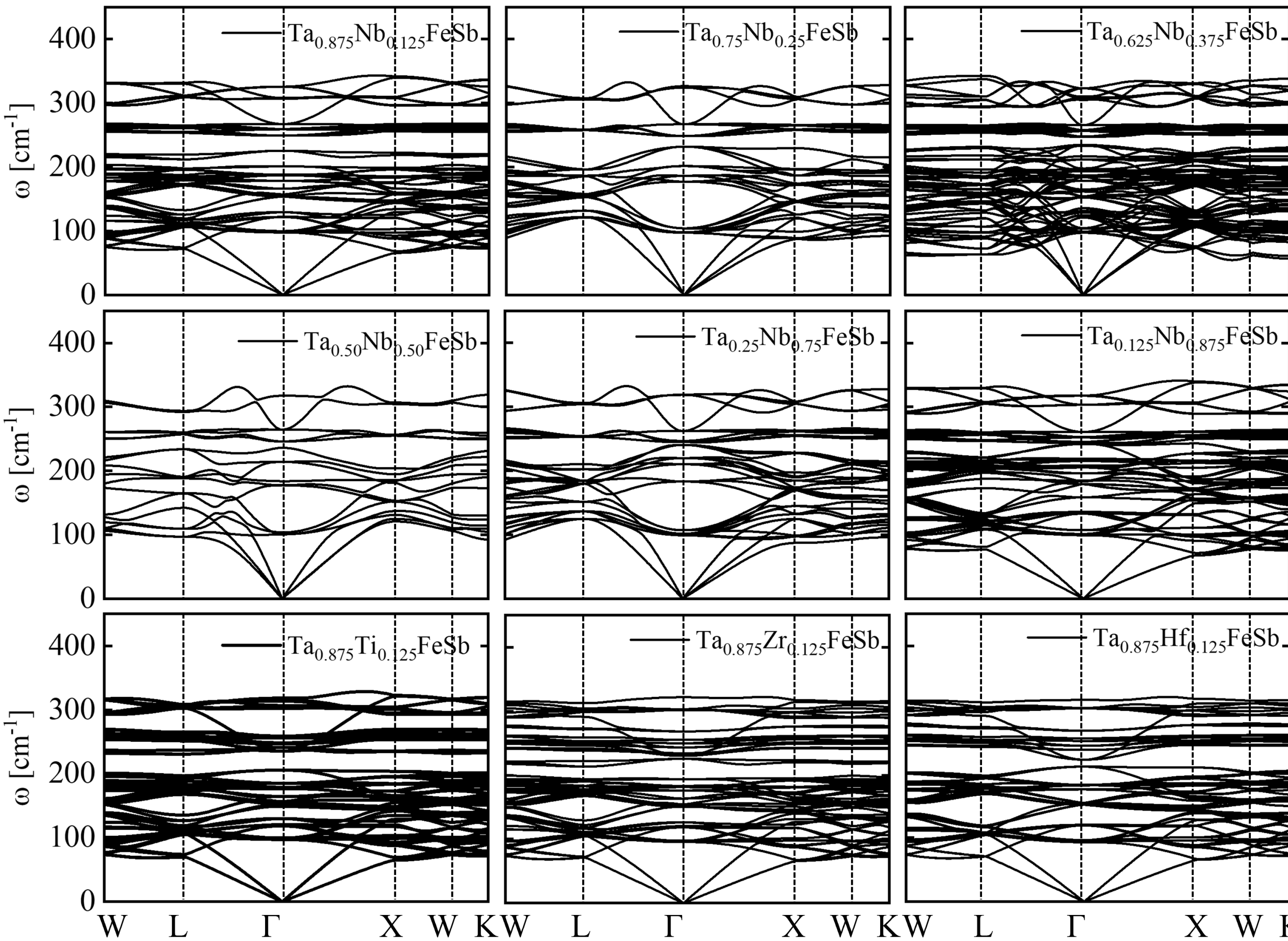

Figure S26: Phonon dispersion of a few compositions considered in the main paper (as mentioned on each sub-figure).

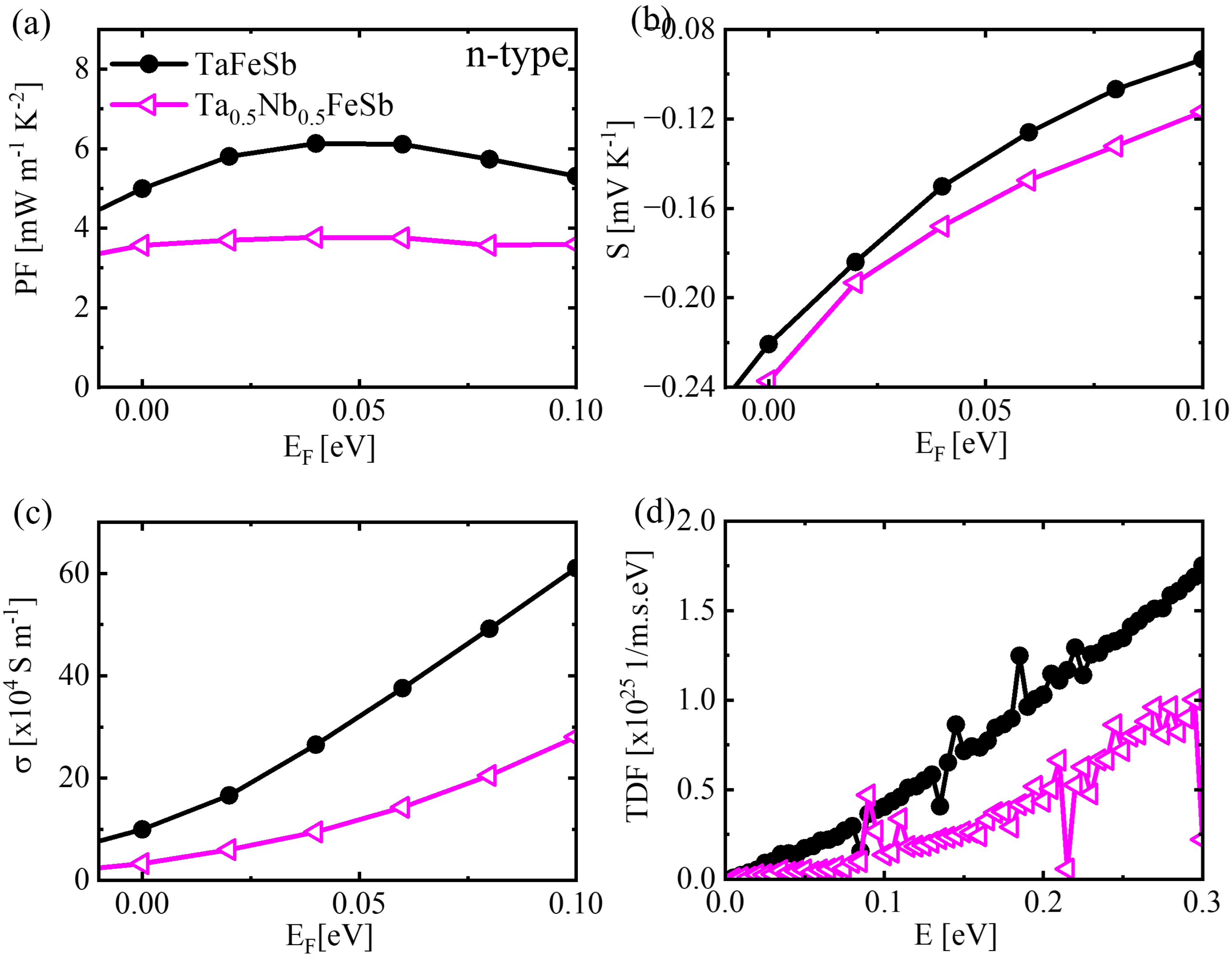

Figure S27: Comparison of: (a) Power factor, (c) Seebeck coefficient, (c) electrical conductivity, and (d) TDF, between the pristine TaFeSb and the $Ta_{0.5}Nb_{0.5}FeSb$ alloy.

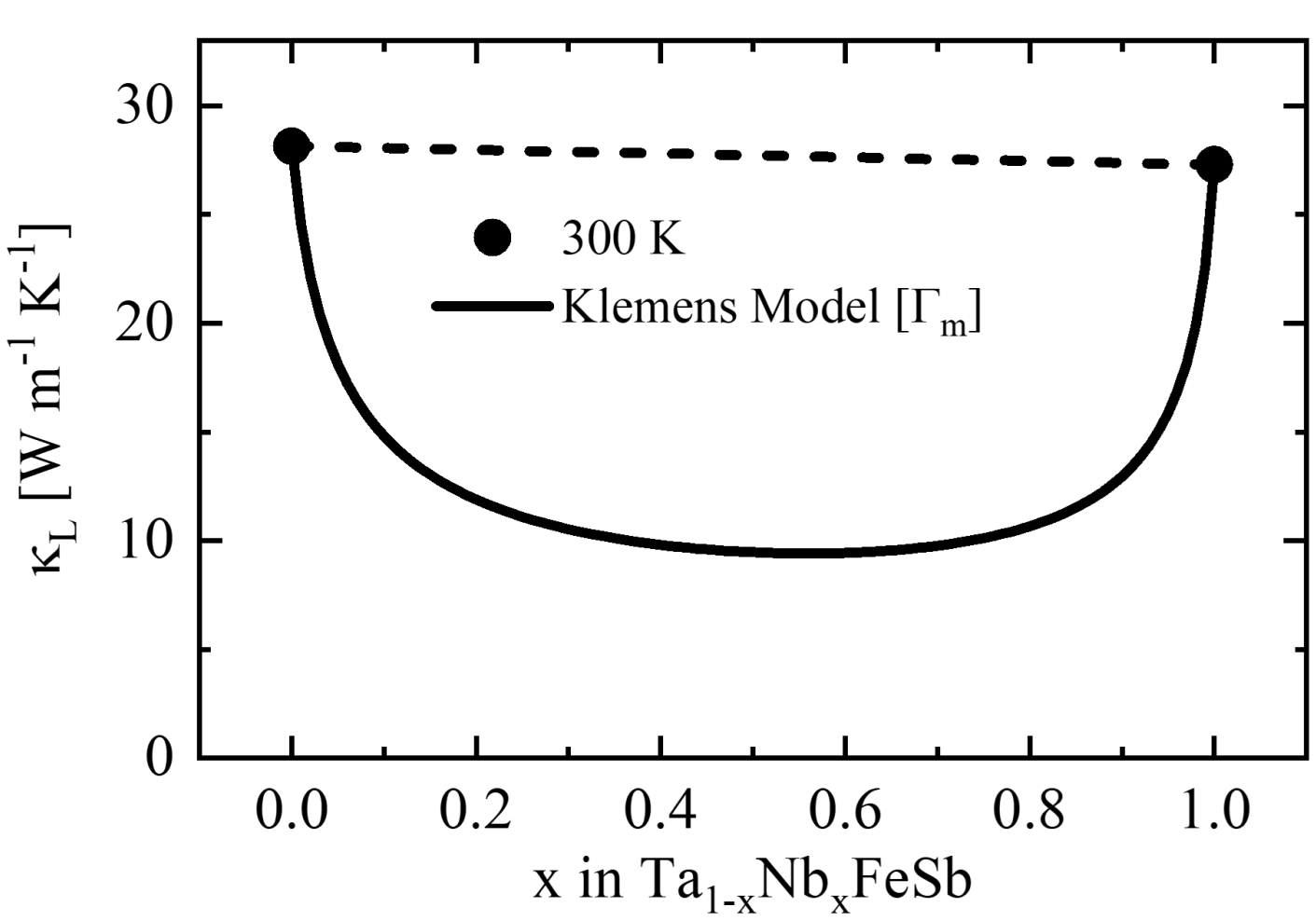

Figure S28: Lattice thermal conductivity as a function of x for $Ta_{1-x}Nb_xFeSb$ calculated using Klemens model.

Scattering treatment for VB of TaFeSb:

The situation for VB is unique, as it has two 2 bands at the same valley (L-point). In the pristine unit cell BZ, $\langle I^2_{kk'} \rangle$ is calculated to be 0.5 for both intra- and inter-band transitions [1, 2]. However, when a supercell of 2×2×2 is created, the 2 bands at the L-point gets folded onto a same Γ-point as shown in Figure S29a,b (L valley is 4 fold degenerate). Now, the situation is complex and simplification is difficult. IIS and POP intra-band scattering can be implemented with $\langle I^2_{kk'} \rangle = 0.5$. But inter-band scattering has to be implemented on these bands pair-wise, i.e. from each band transitions into only one other band transition is allowed with $\langle I^2_{kk'} \rangle = 0.5$, whereas all other inter-band transitions are weak (as in the pristine material - these valleys are farther from each other) and should not be considered. With 8 bands mixed together, it is impossible to identify and implement such pair-wise scattering. The reason is fundamental to DFT calculation and how band numbers are assigned based on energy. So, instead of implementing pairwise inter-band scattering we simplify the treatment using $\langle I^2_{kk'} \rangle = 1$ (for intra-band) and 0 (for inter-band) for all bands (i.e. each bands scatters fully into one band - its own band, rather than with half strength in two bands). We first tested this simplification on the pristine system itself and found to observe negligible error as shown in Figure S29c. We first tested this simplification on the pristine system itself and found to observe negligible error as shown in Figure S29c. With this assumption the PF calculated on 2×2×2 supercell is shown in Figure S29c, which leads to a 23 % error, due to unavoidable mixing of the 8 bands at same Γ-point. So far we could not find any other transformation matrix to avoid band mixing in this case, as we did for the for XNiSn system by choosing the orthorhombic supercell. With Ti/Zr/Hf alloying the PF reduces as shown in Figure S29d. Since the absolute value of the PF has an error of 23 %, we have chosen to show the relative change in the PF with Ti/Zr/Hf alloying in the main text, rather than absolute numbers. In order to further validate our results, we have now included results from a simple parabolic band model considering all 8 bands with individual effective masses listed in Table 4 of SI and their band separation listed at the bottom of Figure 9 (main text). Figure S31 shows a similar change in the PF with Ti/Zr/HF substitution at the Ta site, as when calculated using supercell.

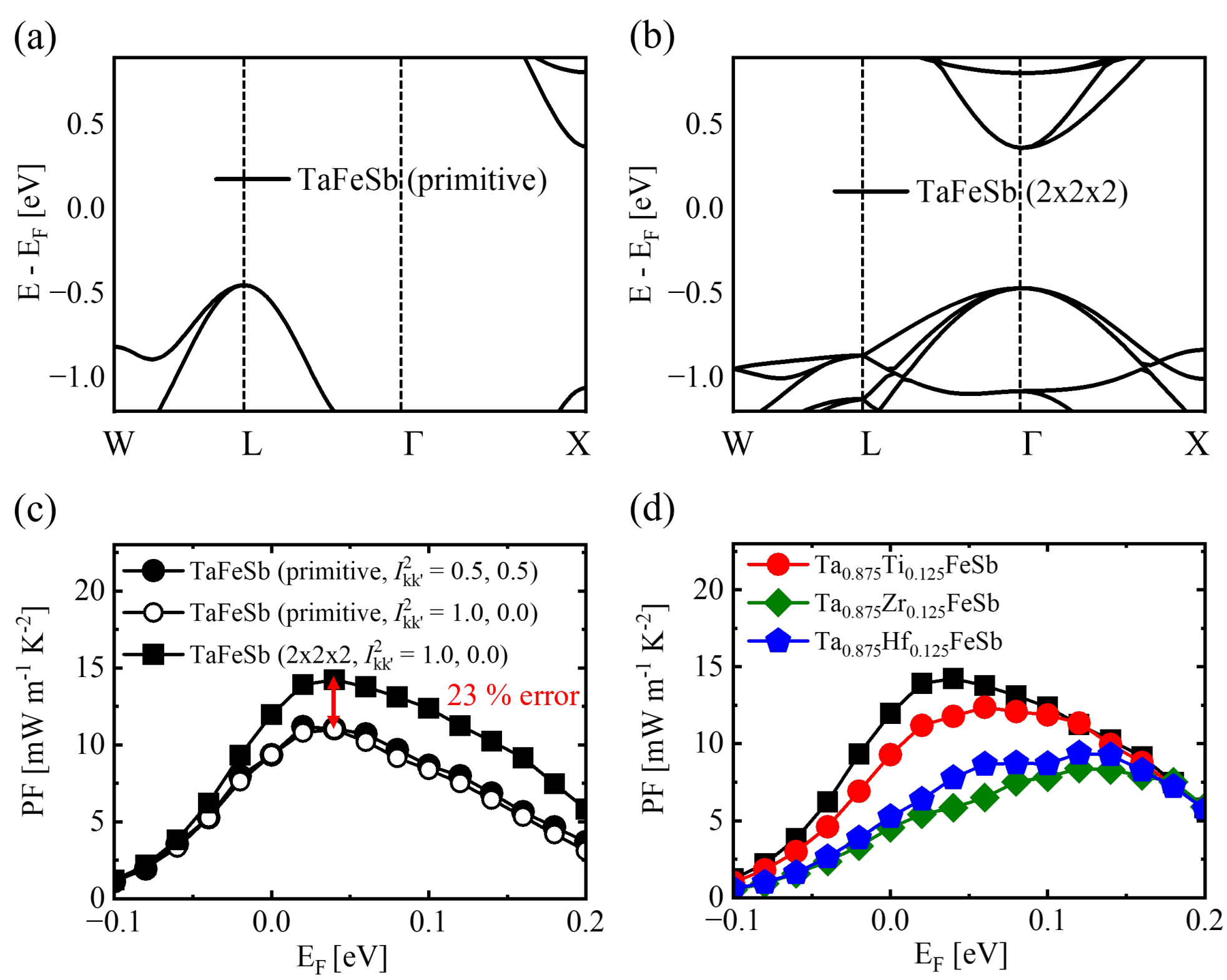

Figure S29: Band structure of (a) primitive TaFeSb, (b) 2×2×2 supercell of TaFesb, (c) Calculated PF using pristine and supercell structure, and (d) PF as a function of Fermi energy for Ti (red), Zr (green), and Hf (blue) doped TaFeSb.

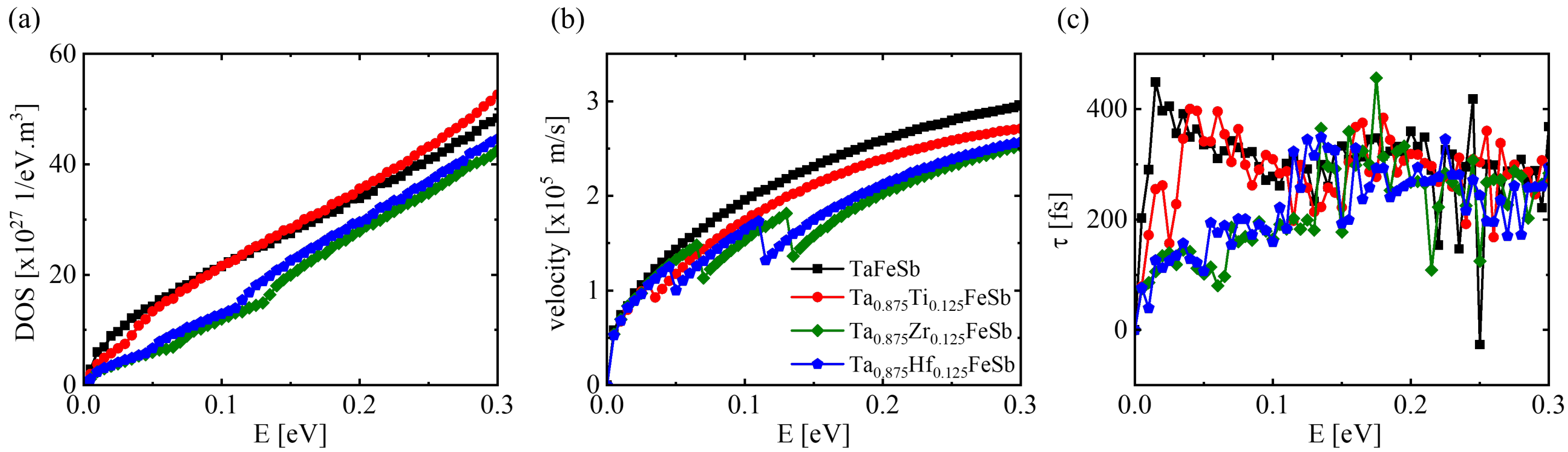

Figure S30: Comparison of: (a) density of states, (b) band velocities, and (c) scattering times at $E_\mathrm{F} = 0$ for the pristine TaFeSb, and the $Ta_{0.875}Ti_{0.125}FeSb$, $Ta_{0.875}Zr_{0.125}FeSb$, and $Ta_{0.875}Hf_{0.125}FeSb$ alloys.

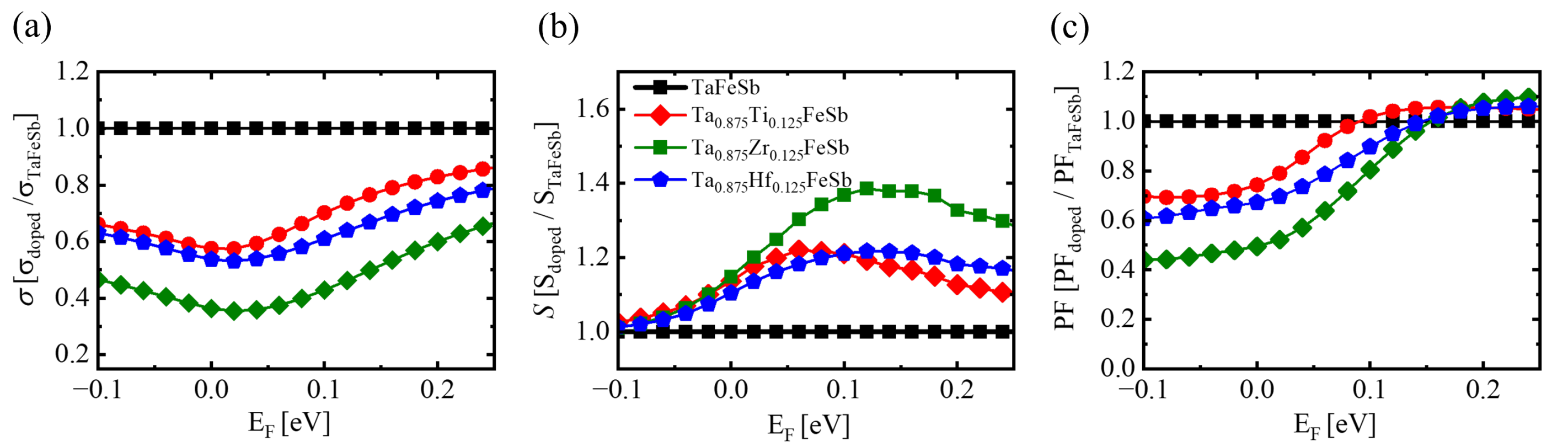

Figure S31: Relative change of: (a) electrical conductivity, (b) Seebeck coefficient, and (c) PF as a function of Fermi energy for Ti (red), Zr (green), and Hf (blue) doped TaFesb with respect to pristine TaFeSb (black), calculated using parabolic band model.

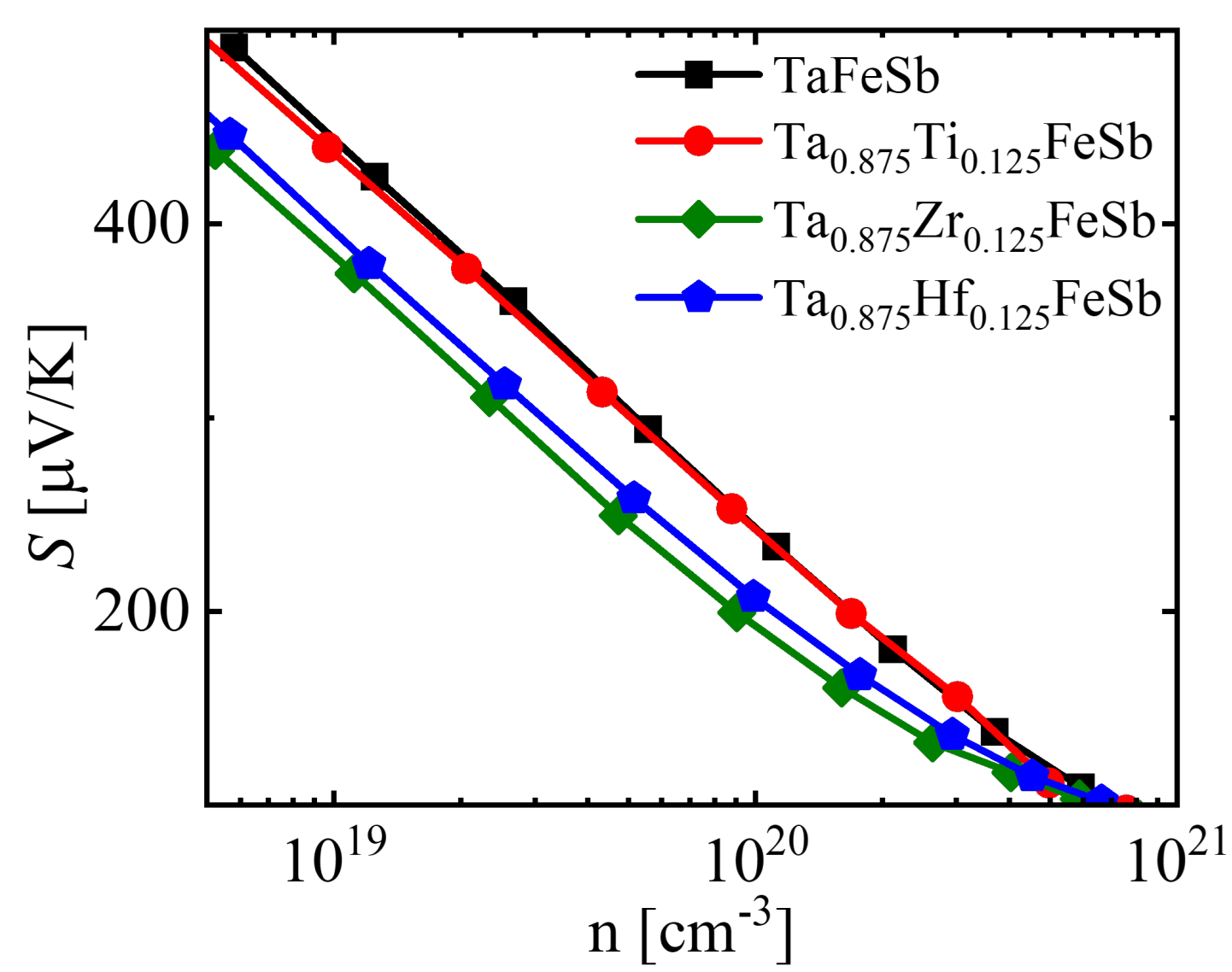

Figure S32: Comparison of absolute Seebeck coefficients versus carrier concentration for the pristine TaFeSb, and $Ta_{0.875}Ti_{0.125}FeSb$, $Ta_{0.875}Zr_{0.125}FeSb$, and $Ta_{0.875}Hf_{0.125}FeSb$ alloys.

References

[1] R. Dutt, B. Sahni, Y. Zhao, Y. Go, S. E. A. Akhtar, A. Kumar, S. Kukreti, P. Graziosi, Z. Li, N. Neophytou, *Journal of Materials Chemistry A* **2026**.

[2] B. Sahni, Y. Zhao, Z. Li, R. Dutt, P. Graziosi, N. Neophytou, *Materials Horizons* **2025**, *12*, 23 10255.

[3] J. Bardeen, W. Shockley, *Physical review* **1950**, *80*, 1 72.

[4] S. Y. Savrasov, D. Y. Savrasov, O. Andersen, *Physical review letters* **1994**, *72*, 3 372.

[5] A. Y. Liu, A. A. Quong, *Physical Review B* **1996**, *53*, 12 R7575.

[6] F. Khan, P. Allen, *Physical Review B* **1984**, *29*, 6 3341.

[7] M. Lundstrom, *Measurement Science and Technology* **2002**, *13*, 2 230.

[8] M. Born, K. Huang, *Dynamical theory of crystal lattices*, Oxford university press, **1996**.

[9] H. Fröhlich, *Advances in Physics* **1954**, *3*, 11 325.

[10] J. Park, J.-J. Zhou, V. A. Jhalani, C. E. Dreyer, M. Bernardi, *Physical Review B* **2020**, *102*, 12 125203.

[11] S. Poncé, E. R. Margine, C. Verdi, F. Giustino, *Computer Physics Communications* **2016**, *209* 116.

[12] J.-J. Zhou, J. Park, I.-T. Lu, I. Maliyov, X. Tong, M. Bernardi, *Computer Physics Communications* **2021**, *264* 107970.

[13] T. Bazhirov, J. Noffsinger, M. L. Cohen, *Physical Review B—Condensed Matter and Materials Physics* **2010**, *82*, 18 184509.

[14] Z. Li, P. Graziosi, N. Neophytou, *npj Computational Materials* **2024**, *10*, 1 8.

[15] S.-Y. Cheng, K.-T. Chen, S. Chang, *Thin Solid Films* **2015**, *584* 135.

[16] D. C. Look, D. K. Lorance, J. R. Sizelove, C. E. Stutz, K. R. Evans, D. W. Whitson, *Journal of Applied Physics* **1992**, *71*, 1 260.

[17] L. Bellaiche, D. Vanderbilt, *Physical Review B* **2000**, *61*, 12 7877.

[18] A. M. Ganose, J. Park, A. Faghaninia, R. Woods-Robinson, K. A. Persson, A. Jain, *Nature communications* **2021**, *12*, 1 2222.

[19] S.-H. Wei, A. Zunger, *Physical Review B* **1999**, *60*, 8 5404.

[20] M. Cardona, N. E. Christensen, *Physical Review B* **1987**, *35*, 12 6182.

[21] Y. Jin, X. Wang, M. Yao, D. Qiu, D. J. Singh, J. Xi, J. Yang, L. Xi, *npj Computational Materials* **2023**, *9*, 1 190.

[22] Q. Jiang, R. Wan, Z. Zhang, Y. Lei, G. Tian, *Physics Letters A* **2022**, *424* 127839.

[23] A. Togo, L. Chaput, I. Tanaka, *Phys. Rev. B* **2015**, *91* 094306.

[24] A. Togo, L. Chaput, T. Tadano, I. Tanaka, *J. Phys. Condens. Matter* **2023**, *35*, 35 353001.

[25] P. Klemens, *Physical review* **1960**, *119*, 2 507.

[26] R. Gurunathan, R. Hanus, G. J. Snyder, *Materials Horizons* **2020**, *7*, 6 1452.

[27] D. Rabin, D. Fuks, Y. Gelbstein, *Physical Chemistry Chemical Physics* **2022**, *25*, 1 520.

[28] J. Yang, G. Meisner, L. Chen, *Applied physics letters* **2004**, *85*, 7 1140.

[29] M. Zhou, L. Chen, W. Zhang, C. Feng, *Journal of applied physics* **2005**, *98*, 1.

[30] H. Geng, H. Zhang, *Journal of Applied Physics* **2014**, *116*, 3.

[31] K. Yadav, S. Singh, O. Muthuswamy, T. Takeuchi, K. Mukherjee, *Journal of Physics: Condensed Matter* **2022**, *34*, 3 035702.

[32] A. Kumar, D. K. Kedia, P. Ghosh, S. Singh, *ACS Applied Energy Materials* **2023**, *6*, 20 10694.

[33] S. Sze, J. Irvin, *Solid-State Electronics* **1968**, *11*, 6 599.

[34] C. Jacoboni, C. Canali, G. Ottaviani, A. A. Quaranta, *Solid-State Electronics* **1977**, *20*, 2 77.

[35] M. Sotoodeh, A. Khalid, A. Rezazadeh, *Journal of applied physics* **2000**, *87*, 6 2890.

[36] P. Graziosi, Z. Li, N. Neophytou, *Computer Physics Communications* **2023**, *287* 108670.

[37] L. Romano, E. Napolitani, V. Privitera, S. Scalese, A. Terrasi, S. Mirabella, M. Grimaldi, *Materials Science and Engineering: B* **2003**, *102*, 1 49, e-MRS 2002 Symposium E: Advanced Characterisation of Semiconductors.

[38] C. B. Vining, W. Laskow, J. O. Hanson, R. R. Van der Beck, P. D. Gorsuch, *Journal of applied physics* **1991**, *69*, 8 4333.

[39] M. Steele, F. Rosi, *Journal of Applied Physics* **1958**, *29*, 11 1517.

[40] Q. Xu, J. Zhou, T.-H. Liu, G. Chen, *Physical Review Applied* **2021**, *16*, 6 064052.

[41] S. Foster, N. Neophytou, *Journal of Electronic Materials* **2019**, *48*, 4 1889.

[42] S. Foster, N. Neophytou, *Computational Materials Science* **2019**, *164* 91.

[43] P. Graziosi, N. Neophytou, *The Journal of Physical Chemistry C* **2020**, *124*, 34 18462.

[44] Y. Liu, H. Xie, C. Fu, G. J. Snyder, X. Zhao, T. Zhu, *Journal of Materials Chemistry A* **2015**, *3*, 45 22716.

[45] S. Chen, K. C. Lukas, W. Liu, C. P. Opeil, G. Chen, Z. Ren, *Advanced Energy Materials* **2013**, *3*, 9 1210.

[46] N. S. Chauhan, S. Bathula, A. Vishwakarma, R. Bhardwaj, K. K. Johari, B. Gahtori, M. Saravanan, A. Dhar, *Journal of Physics and Chemistry of Solids* **2018**, *123* 105.

[47] G. Mesaritis, I. Ioannou, A. Delimitis, E. Hatzikraniotis, Y. Gelbstein, T. Kyratsi, *Journal of Physics and Chemistry of Solids* **2022**, *167* 110735.

[48] S.-W. Kim, Y. Kimura, Y. Mishima, *Intermetallics* **2007**, *15*, 3 349.

[49] M. Gürth, G. Rogl, V. Romaka, A. Grytsiv, E. Bauer, P. Rogl, *Acta Materialia* **2016**, *104* 210.

[50] A. Kumar, S. Vishak, P. Ghosh, S. Singh, *Small* **2026**, *22*, 37 e73765.
